# Group-specific discriminant analysis enhances detection of sex differences in brain functional network lateralization

**DOI:** 10.1093/gigascience/giaf082

**Published:** 2025-08-30

**Authors:** Shuo Zhou, Junhao Luo, Yaya Jiang, Haolin Wang, Haiping Lu, Gaolang Gong

**Affiliations:** School of Computer Science, University of Sheffield, S1 4DP, Sheffield, UK; Centre for Machine Intelligence, University of Sheffield, S1 3JD, Sheffield, UK; Neuroscience Institute, University of Sheffield, S10 2TN, Sheffield, UK; State Key Laboratory of Cognitive Neuroscience and Learning & IDG/McGovern Institute for Brain Research, Beijing Normal University, 100875, Beijing, China; Shenzhen CyberAray Network Technology Co., Ltd, 518038, Shenzhen, China; Harbin Institute of Technology (Shenzhen), 518055, Shenzhen, China; State Key Laboratory of Cognitive Neuroscience and Learning & IDG/McGovern Institute for Brain Research, Beijing Normal University, 100875, Beijing, China; Artificial Intelligence and Language Cognition Laboratory, Beijing International Studies University, 100024, Beijing, China; School of Computer Science, University of Sheffield, S1 4DP, Sheffield, UK; Centre for Machine Intelligence, University of Sheffield, S1 3JD, Sheffield, UK; School of Computer Science, University of Sheffield, S1 4DP, Sheffield, UK; Centre for Machine Intelligence, University of Sheffield, S1 3JD, Sheffield, UK; Neuroscience Institute, University of Sheffield, S10 2TN, Sheffield, UK; State Key Laboratory of Cognitive Neuroscience and Learning & IDG/McGovern Institute for Brain Research, Beijing Normal University, 100875, Beijing, China; Beijing Key Laboratory of Brain Imaging and Connectomics, Beijing Normal University, 100875, Beijing, China; Chinese Institute for Brain Research, 102206, Beijing, China

**Keywords:** group-specific analysis, sex-specific lateralization, brain functional network, dual-classification, group-specific discriminant analysis

## Abstract

**Background:**

Lateralization is the asymmetry in function and cognition between the brain hemispheres, with notable sex differences. Conventional neuroscience studies on lateralization use univariate statistical comparisons between male and female groups, with limited and ineffective validation for group specificity. This article proposes to model sex differences in brain functional network lateralization as a dual-classification problem: first-order classification of left versus right hemispheres and second-order classification of male versus female models. To capture sex-specific patterns, we developed an interpretable group-specific discriminant analysis (GSDA) for first-order classification, followed by logistic regression for second-order classification.

**Findings:**

Evaluations on 2 large-scale neuroimaging datasets show GSDA’s effectiveness in learning sex-specific patterns, significantly improving model group specificity over baseline methods. Major sex differences were identified in the strength of lateralization and interaction patterns within and between lobes.

**Conclusions:**

The GSDA-based analysis challenges the conventional approach to investigating group-specific lateralization and indicates that previous findings on sex-specific lateralization will need revisits and revalidation. This method is generic and can be adapted for other group-specific analyses, such as treatment-specific or disease-specific studies.

Key Points

- Conventional multivariate and univariate methods identified common but not specific lateralization patterns through within-group analysis.
- Our group-specific discriminant analysis (GSDA)–based method identified sex-specific lateralization patterns, validated through cross-validation and shown to be distinct from those identified by conventional methods.
- Nearly half of the specifically lateralized functional connections are shared by both males and females, with sex differences observed in the strength of lateralization.
- Stronger positive interlobe interactions are more left-lateralized in male brain networks, whereas stronger positive intralobe interactions are more right-lateralized in female brain networks.

## Background

Human brains are functionally asymmetric [1, 2]. These differences between left and right brain hemispheres are believed to reflect a complex interplay of evolutionary, hereditary, developmental, experiential, and pathological influences [3–6]. One important understanding is that multiple factors influence human brain lateralization [7], with sex being one of the most representative [8–11]. A popular hypothesis is that males typically have a more asymmetric brain organization, with the left hemisphere specialized for verbal processing and the right for spatial processing. In contrast, females tend to have a more “bilateral” brain organization, where both hemispheres are involved in verbal processing. The origin of such sex differences in functional lateralization has been attributed to neurobiological mechanisms involving genetic and hormonal factors [7, 12]. Genetically, X-inactivation in females leads to cortical mosaicism and functional flexibility [13], and Y-linked genes in males promote asymmetric development [14]. Hormonally, testosterone is associated with promoting right hemisphere dominance through delayed maturation of the left hemisphere [15], while estrogen enhances interhemispheric connectivity [16–20]. Moreover, recent brain magnetic resonance imaging (MRI) studies [21–23] have shown that males tend to have smaller corpus callosums and larger amygdalae, which may limit interhemispheric communication. In contrast, females typically have larger corpus callosums that enhance integration between hemispheres. These findings potentially explain the more lateralized brain organization in males and the more bilateral organization in females.

Neurobiological sex differences and brain lateralization have also been observed in several psychiatric disorders, as reported in multiple brain MRI studies [24–27]. For example, depression, anxiety, schizophrenia, and autism spectrum disorder exhibit notable sex differences in incidence rates and clinical manifestations [24, 25]. Additionally, several studies on brain MRIs have demonstrated that brain lateralization abnormalities are linked with conditions such as major depressive disorder (MDD) [26] and schizophrenia [27]. Understanding these sex-specific and lateralized brain alterations may contribute to personalized diagnosis, prognosis, treatment, and further uncovering the pathogenesis of these diseases.

Measuring brain functional lateralization is valuable but challenging [28]. Direct approaches such as selectively modulating or suppressing cortical activities and circuits in a single hemisphere [29] often pose a risk of inflicting harm on the human brains [30]. Over the past 2 decades, functional neuroimaging techniques have been widely used in neuroscience, offering a powerful and noninvasive approach for investigating brain lateralization [31, 32]. One popular technique is analyzing functional connectivity (FC), also known as brain networks or connectomes [33]. This is usually derived from resting-state functional MRI (rs-fMRI) time series and considered an intrinsic “fingerprint” of each individual’s brain [34–36]. A previous study [10] reported sex differences in the lateralization of resting-state networks, with more right-lateralized visual and default-mode network components for males and females, respectively. Additionally, males and females have also demonstrated significant differences in homotopic functional connectivity of various regions [37].

Studies on brain lateralization have largely focused on modeling asymmetry effects region by region via univariate analysis [7]. These lateralized brain regions are usually measured using the laterality index (LI) [4, 6, 38, 39] or identified through statistical tests comparing homologous regions [40]. Previous analyses identifying sex-specific brain lateralization have typically adopted within-group univariate methods [22, 41–43]. For example, to understand male-specific lateralization, analyses are performed separately on male and female data, labeling features that significantly differ from female data as “male-specific.” There are 2 main limitations to this approach. First, univariate approaches generally lack robust, data-driven validation, meaning models derived from these analyses cannot be tested on unseen data samples. Specifically, for identifying sex-specific lateralization patterns, univariate frameworks cannot provide effective mechanism for validating the generalizability and specificity of models obtained separately from male and female samples, raising questions about the reliability of such results and findings. Second, univariate analyses may not be able to capture complex interactions among multiple neuroimaging features, potentially overlooking critical multivariate patterns underlying sex differences in lateralization. Additionally, the substantial anatomical and functional similarities between male and female brains, combined with typically small statistical effect sizes, further complicate the reliable detection of genuine sex differences in lateralization [44]. Consequently, subtle sex-specific lateralization patterns may be overshadowed by broad similarities.

**Figure 1:**
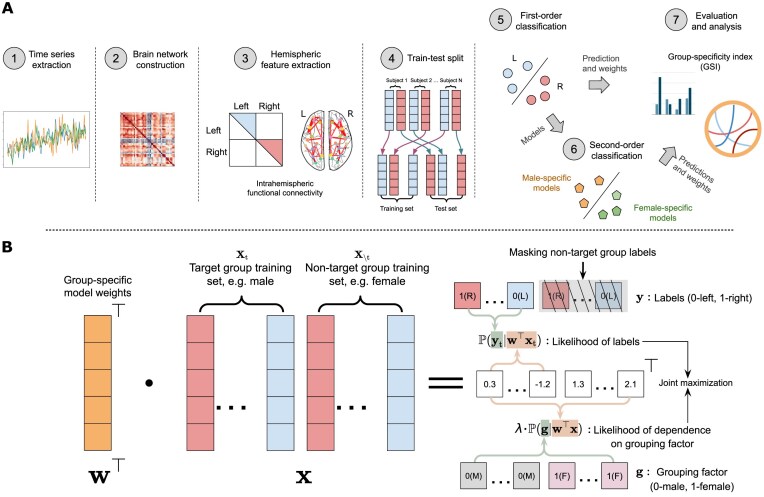
The proposed workflow for detecting sex-specific brain lateralization via GSDA. (A) Overview of the proposed classification workflow. ①–③: Hemispheric features are extracted from the intrahemispheric brain functional network, constructed from resting-state functional MRI time series. ④⑤: First-order classification learns the differences between the 2 hemispheres, where we propose a GSDA classifier to classify left versus right hemispheres for a target group. ⑥: Second-order classification trains a standard logistic regression for classifying the male- versus female-specific models obtained from the first-order classification, to identify the weights that significantly contribute to the sex-specific predictions. ⑦: Prediction results evaluation and model weight interpretation. (B) GSDA with the logistic loss (GSDA-Logit) for the first-order classification. This model jointly maximizes the likelihood of labels for the target group (with nontarget group labels masked out) and the grouping factor dependence for both the target and nontarget groups, where $^\top$ denotes the transpose of vectors, $\mathbf {x}$ denotes the input training samples, $\mathbf {x}_{\mathrm{t}}$ denotes target group training samples, $\mathbf {x}_{\setminus \mathrm{t}}$ denotes nontarget group samples, and a hyperparameter $\lambda \ge 0$ controls the grouping factor dependence. A larger $\lambda$ corresponds to a higher dependence. When $\lambda =0$, GSDA-Logit degenerates to a standard logistic regression for the target group data.

Here, we address the challenges of detecting and validating sex differences in brain lateralization by framing the problem as a machine learning classification task, making the following key methodological contributions: First, we propose a dual-classification workflow to identify, validate, and interpret multivariate patterns of sex-specific lateralization. This consists of a first-order classification of left versus right brain hemispheres and a second-order classification of male- versus female-specific models. The resulting model weights represent lateralization strength and sex difference significance, respectively. Figure 1A presents the whole workflow. Second, we propose a novel group-specific discriminant analysis (GSDA) algorithm (Fig. 1B) to learn group (sex)–specific models in the first-order classification. Third, we leverage cross-validation to statistically evaluate the learned lateralization patterns by assessing model accuracy on male and female test samples. Furthermore, we propose a group specificity index (GSI) to measure the group specificity of the learned models.

Our final contribution provides interpretation for the experimental results using the intrahemispheric connections extracted from rs-fMRI data of 2 large-scale public neuroimaging repositories, the Human Connectome Project (HCP) [45] and the Brain Genomics Superstruct Project (GSP) [46]. A significant GSI improvement over the baselines demonstrates the effectiveness of GSDA in learning group-specific models. Further interpretation of the dual-classification model weights reveals consistent sex differences in lateralization across datasets: (i) about half of the sex-specific lateralized connections are shared between male and female brain functional network, with differences in the strength of lateralization, and (ii) stronger positive interlobe interactions are more left-lateralized in the male brain networks, while stronger positive intralobe interactions are more right-lateralized in the female brain networks.

## Results

### Diverged test accuracy on male and female sets

**Figure 2:**
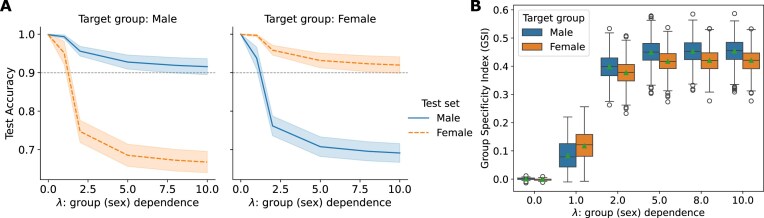
Diverged test accuracy and improved GSI of left versus right brain classification on male and female sets from the HCP [45] using the proposed GSDA with the logistic loss (GSDA-Logit) with respect to the hyperparameter $\lambda$, which controls the grouping factor (sex) dependence. There were 1,000 random training–testing partitions for the experiment, where each subject randomly contributed 1 hemisphere for training and the other for testing, resulting in 50% brain hemispheres being selected as training samples. (A) The test accuracy on male and female sets increasingly diverges with the increase of dependence on sex ($\lambda$). The average test accuracy is represented by solid or dashed lines, with standard deviations shown as error bands, computed across 1,000 random training–testing partitions. (B) The GSI calculated from the results in Fig. 2A increases with $\lambda$. The horizontal lines in each box represent the 25th percentile, median, and 75th percentile of the GSI over the 1,000 test sets, respectively, from bottom to top, and the green triangles represent the mean. The average GSI approaches 0 when $\lambda =0$, indicating that models learned without group dependence regularization captured common instead of specific patterns, despite being trained on only male (or female) data.

Figure 2 depicts the performance of GSDA in classifying left versus right brain hemispheres on the HCP data [45], across a varied range of values for hyperparameter $\lambda$. A larger $\lambda$ indicates a higher grouping factor (sex) dependence. When the target group is male (the left of Fig. 2A), the labels for the left and right hemispheres of the female training data were masked. Therefore, the training female samples were only involved in the grouping factor dependence regularization. In this scenario, the average accuracy obtained on the male test samples (the blue solid line) stays higher than that on the female test samples (the orange dashed line). The increase of $\lambda$ leads to an increased gap between the test accuracy on target and nontarget test sets. In particular, this discrepancy widens significantly within the range $0<\lambda \le 5$ and stabilizes to a 20% gap for $\lambda > 5$ (Fig. 2A). These observations remain consistent in results with 2 different cross-validation strategies for the HCP data (Supplementary Fig. S1A, C) and the GSP data (Supplementary Fig. S2A, C). A theoretical interpretation is provided in the Methods section to validate this divergence.

The group specificity of models obtained by GSDA increases with a larger $\lambda$, as reflected by our proposed metric, the GSI, which is presented as a boxplot in Fig. 2B. When $0<\lambda \le 5$, the GSI for both male- and female-specific GSDA models increases with the increase of $\lambda$. When $\lambda \ge 5$, the GSI maintains at around 0.4. Based on both accuracy and GSI results, $\lambda =5$ is an “elbow” point in the experiment across different datasets and cross-validation strategies, which can be considered an optimal value for the trade-off between classification accuracy, group specificity, and model complexity (the hyperparameter for the $\ell _2$ regularization in GSDA was fixed to 0.1, so the larger the $\lambda$, the lower the relative importance of the $\ell _2$ regularization). Hence, in the rest of this article, we will use $\lambda = 5$ for GSDA as the main sex-specific model to present the results and findings.

In contrast, the GSI steadily approaches zero without the grouping factor dependence regularization. At $\lambda =0$, where GSDA degenerates to a standard logistic regression trained only on the target group hemispheres, the accuracy is nearly 100% for both male and female test samples (Fig. 2A, Table 1, Supplementary Figs. S1A, C and S2A, C). This performance is similar to the multivariate control baseline, which uses standard logistic regression trained on mixed male and female hemispheres. From Table 1, the control models achieved an accuracy of 99.99% $\pm$ 0.04% for male and 99.92% $\pm$ 0.13% for female HCP test samples, as well as 99.94% $\pm$ 0.07% for male and 99.99% $\pm$ 0.01% for female GSP test samples. Additionally, because of the same property and similar performance compared to the standard logistic regression (multivariate control baselines), we will view GSDA with $\lambda =0$ as an additional multivariate baseline.

**Table 1:** First-order classification (left versus right brain hemispheres) accuracy on male and female test sets from the HCP [45] and Brain Genomics Superstruct Project (GSP) [46]. Group-specific models (GSDA with $\lambda =5$) are compared with 3 multivariate baselines: (i) standard logistic regression trained on a mixture of male and female training data, (ii) GSDA with $\lambda =0$ (equivalent to standard logistic regression) trained on male data only, and (iii) GSDA with $\lambda =0$ trained on female data only. $\lambda =5$ is an optimal value for GSDA on the data, as determined by the accuracy and GSI in Fig. 2. The baselines achieved similar accuracy on male and female test sets, indicating a lack of group specificity. Conversely, the group-specific models maintained accuracy on the target test set but showed a significant gap with the lower accuracy on the nontarget test set.

Classification method (target group)	Average test accuracy (%) and gap ($|\text{accuracy difference}|$)					
	HCP male	HCP female	HCP gap	GSP male	GSP female	GSP gap
Logistic regression (male + female)	99.99 $\pm$ 0.04	99.96 $\pm$ 0.10	0.03	99.93 $\pm$ 0.13	99.96 $\pm$ 0.10	0.05
GSDA ($\lambda =0$, male)	99.87 $\pm$ 0.16	99.85 $\pm$ 0.17	0.02	99.93 $\pm$ 0.08	99.99 $\pm$ 0.01	0.06
GSDA ($\lambda =0$, female)	99.93 $\pm$ 0.12	99.99 $\pm$ 0.04	0.06	99.97 $\pm$ 0.05	99.95 $\pm$ 0.07	0.02
GSDA ($\lambda =5$, male)	92.75 $\pm$ 1.83	68.52 $\pm$ 2.88	24.23	91.85 $\pm$ 1.77	71.28 $\pm$ 2.13	20.57
GSDA ($\lambda =5$, female)	70.76 $\pm$ 2.56	93.16 $\pm$ 1.89	22.40	74.70 $\pm$ 2.22	92.81 $\pm$ 1.35	18.11

### GSDA-based models learned distinct weights

**Figure 3:**
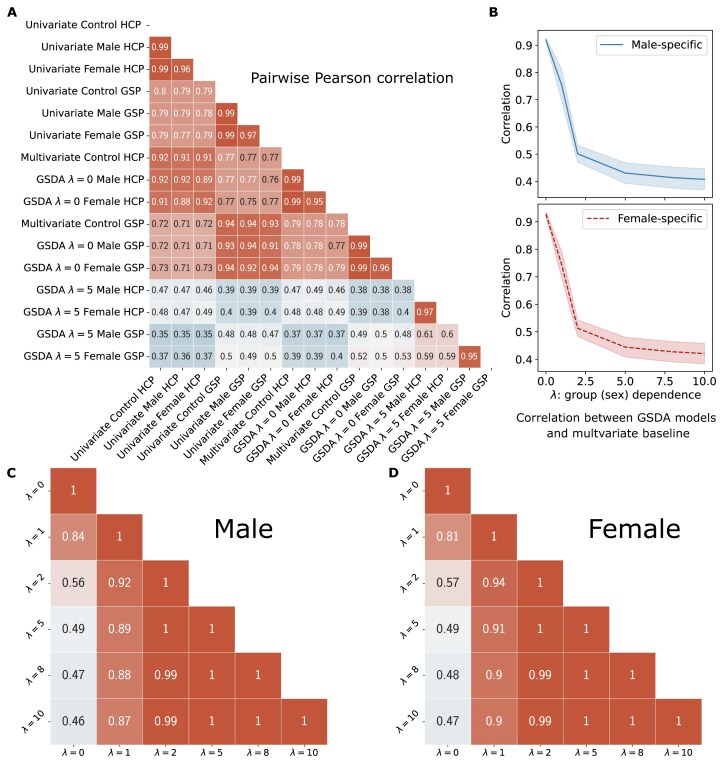
Pearson correlation between model weights. (A) Pairwise correlation between weights of 16 models, including multivariate models from Table 1 and univariate models, labeled along the $x$- and $y$-axis. Two clusters can be observed here: multivariate and univariate baselines versus GSDA with $\lambda =5$. (B) Correlation between GSDA and multivariate control models (trained on mixed male and female data) on the HCP data. As $\lambda$ increases, the GSDA models become less correlated with the control models. (C, D) Average pairwise correlation for (C) male-specific and (D) female-specific GSDA models trained on HCP data, with respect to $\lambda$. The weights of sex-specific models remain stable (correlation $\ge 0.99$) for $\lambda \ge 2$.

Beyond classification performance similarity, the weights of multivariate baselines (control and GSDA with $\lambda =0$) are also highly correlated. As shown in Fig. 3A, the average Pearson correlation coefficients between multivariate baselines are 0.99 for analyses conducted within either HCP or GSP data. Similarly, in univariate analyses based on the $t$-test of paired left and right connections, the $t$-values of within-group analysis showed a 0.99 correlation with the $t$-values derived from mixed male and female samples (univariate control). Among these multivariate and univariate baselines, the correlation for any arbitrary pair exceeds 0.91 for within-dataset results and 0.7 for cross-dataset results. These correlations represent large ($\ge 0.8$) and medium ($0.5-0.8$) effects, respectively, according to the thresholds for interpreting the effect size of Pearson’s correlation [47, 48]. This high correlation suggests that the lateralization modeled by multivariate or univariate baselines is common to both males and females, regardless of whether the analysis is conducted with exclusively male or female data, or with mixed data. This corresponds to the top red triangular cluster in Fig. 3A.

In contrast, our sex-specific models (with a higher GSI) show lower correlations with the univariate and multivariate baseline models. This corresponds to the blue rectangular cluster at the bottom of Fig. 3A, where a majority of coefficients fall within the range of 0.35 to 0.5 (below the thresholds of medium effects [47, 48]). Increasing the value of $\lambda$ leads to a decreasing correlation between the control and GSDA models ($\lambda >0$), for both results from HCP (Fig. 3B and first columns of Fig. 3C, D) and GSP (Supplementary Fig. S3A, B and first columns of Supplementary Fig. S3C, D). Moreover, the weights of sex-specific models are stable. As shown in Fig. 3C, D, the average correlation of any pair for GSDA with $\lambda \ge 2$ is 0.99 or above.

### Frontal lobe shows most sex-specific lateralized connections

To identify sex-specific lateralized connections among the 7,503 intrahemispheric connections, we performed a second-order classification. This involved training standard logistic regression models to distinguish between male- and female-specific models learned from the first-order classification, using 80% of the first-order models for training and 20% for testing. The test accuracy for second-order classification consistently achieved nearly 100% over 1,000 random splits. This indicates that the sex differences in the first-order GSDA model weights are generalizable from the training set to the test set.

**Figure 4:**
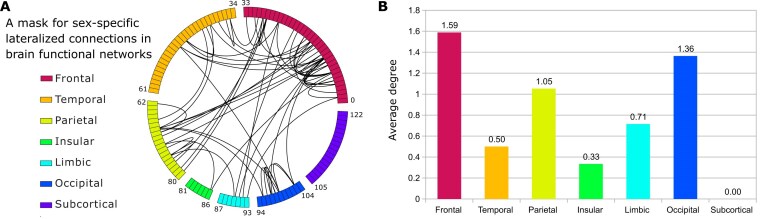
Sex-specific lateralized functional connection mask derived from GSDA-based dual classification. (A) A chord-based mask for identifying sex-specific lateralized connections was derived in 2 steps: (i) averaging weights across 1,000 second-order models from different random splits for HCP and GSP data, respectively, and (ii) identifying overlaps between the top 5% largest average weights from HCP and those from GSP. The circle represents a brain hemisphere, and each cell on the rim represents a region of interest (ROI) within the half brain. The 7 colors indicate 7 functional parcellations defined in the Brainnetome Atlas (BNA) [50]. The numbers on the rim are the start and end ROI IDs of the lobe in the BNA, where the 123 ROIs are labeled from 0 to 122. (B) The average lateralization degree [49] for each of the 7 BNA lobes. It is calculated as the average number of chords per ROI, based on the chords and ROIs in Fig. 4A. The frontal lobe shows the largest degree of lateralization.

Based on the weights from these second-order classification models, we derived a mask that characterizes sex differences in the lateralized connections. We first averaged the weights across 1,000 second-order models from different random splits for the HCP and GSP datasets, respectively. Then, we identified the overlap between the top 5% of the largest average weights (by magnitude) from HCP and those from GSP. The resulting map is represented by the chords in Fig. 4A. The threshold of 5% was chosen because the second-order logistic regression classifiers were trained with $\ell _2$ regularization, which can be interpreted as a Gaussian prior (normal distribution) on model weights, with 5% being a commonly used statistical significance level for a Gaussian distribution. By calculating the average degree [49] (Fig. 4B) of connections for each lobe within this mask, we can learn that sex differences in first-order weights are associated with the frontal, parietal, and occipital lobes, where the average degrees exceed 1. The frontal lobe shows the largest average degree, indicating significant sex differences.

**Figure 5:**
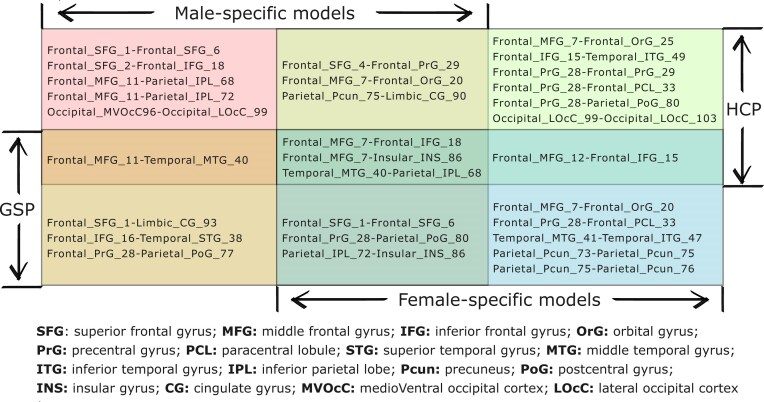
Thirty unique sex-specific lateralized connections learned from HCP and GSP. Each connection is represented as “ROI-ROI,” and each ROI is represented as “Lobe_Gyrus_ROI ID,” where the lobe and gyrus are defined in BNA. Twenty-two of the 30 connections are associated with the frontal lobe. Nine connections are shared between males and females (middle column), with the differences between male and female models being in the strength of lateralization (Fig. 6A–D). Twenty-one connections are “exclusive” to 1 group (left and right columns), with the differences between male and female models being in the patterns of inter-/intralobe interactions (Fig. 6E–F, Fig. 7).

**Figure 6:**
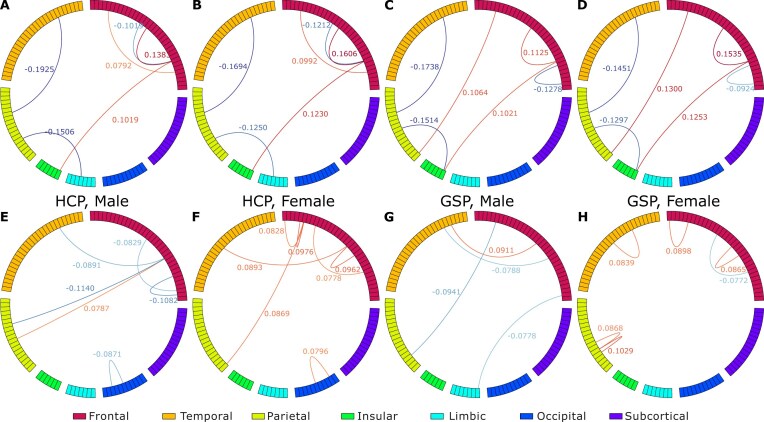
Sex-specific lateralized connections with first-order GSDA model ($\lambda =5$) weights. The connections were identified by applying the mask in Fig. 4A to the top 5% weights (by magnitude) from 4 models specific to males of HCP (A, E), females of HCP (B, F), males of GSP (C, G), and females of GSP (D, H). Each of the 4 models was obtained by averaging the corresponding 1,000 first-order models. The sex-specific lateralized connections consist of shared connections between male and female models (A–D) and the group (sex) “exclusive” connections (E–H). The weights of these shared connections show consistent sex differences. In female-specific models, the weights for connections involving the frontal lobe tend to be larger than those in male-specific models, especially for positive weights. Conversely, in male-specific models, the weights for connections to other lobes are generally larger than those in female-specific models. The “exclusive” connections in male-specific models are mostly interlobe and negative, whereas in female-specific models, they are mostly intralobe and positive. Statistics about these connections can be found in Fig. 7.

We then applied this mask to the top 5% weights of 4 first-order classification models: HCP male-specific, HCP female-specific, GSP male-specific, and GSP female-specific. The obtained lateralized connections with sex differences are shown in Fig. 5, Fig. 6A–H, and Supplementary Fig. S4A–D. The weights of these 4 models were obtained by taking the average of the corresponding 1,000 models learned from first-order classification with different random splits. In total, 47 lateralized connections with repetition were identified, of which 30 connections are unique. Among these 47 connections, the middle frontal gyrus (MFG) was the most frequently involved region, suggesting it may serve as a hub. Specifically, 17 out of the 47 connections were associated with the MFG in both male and female samples across both datasets.

### Sex-specific lateralization: shared and “exclusive” connections

For each of the 4 sex-specific models, half of the identified lateralized connections are shared between male and female brain networks (Fig. 5) on average: for HCP, 6 of 12 for the male-specific model and 6 of 13 for the female-specific model; for GSP, 6 of 10 for the male-specific model and 6 of 12 for the female-specific model. Among the 30 unique sex-specific lateralized connections identified across datasets, 9 (nearly one-third) are shared between males and females. To illustrate these findings, we have separated the shared connections (Fig. 6A–D) and “exclusive” connections (Fig. 6E to H).

For the shared lateralized connections (Fig. 6A, B for HCP and Fig. 6C, D for GSP), we observed sex differences in the magnitude of first-order weights (i.e., the strength of lateralization). Specifically, for the female-specific models, the magnitudes of the first-order weights corresponding to the connections associated with the frontal lobe are generally larger compared to those for male-specific models, particularly those of the positive weights. In the male-specific models, the magnitudes of the first-order weights for connections related to other lobes are larger than those in the female-specific models.

**Figure 7:**
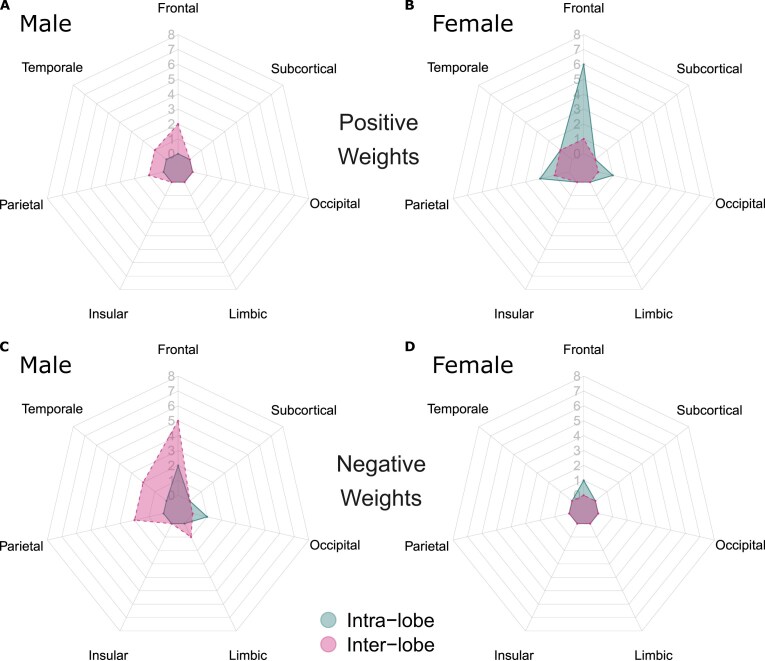
Count of the group “exclusive” lateralized connections for HCP and GSP (Fig. 6E–H), categorized by associated lobes, inter- or intralobe, and signs of the first-order weights. The connections were identified by (A) the male-specific models with positive first-order weights, (B) the female-specific models with positive first-order weights, (C) the male-specific models with negative first-order weights, and (D) the female-specific models with negative first-order weights. In male brain networks, 7 of 10 “exclusive” connection counts are interlobe, with 71.4% of the interlobe connections having negative first-order weights. In female brain networks, 11 of 13 “exclusive” connection counts are intralobe, with 91.7% of the intralobe connections having positive first-order weights.

For the “exclusive” connections (Fig. 6E–H), the male-specific models contain more interlobe lateralized connections (Fig. 7A), with over 70% of corresponding weights being negative, shown by the blue chords in Fig. 6E, G. Female-specific models, on the other hand, contain more intralobe lateralized connections (Fig. 7B), with over 90% of weights being positive, indicated by the red chords in Fig. 6F, H. Notably, these patterns of inter- and intralobe lateralization for males and females are consistent across joint or separate analyses of both HCP and GSP data (Supplementary Fig. S5), demonstrating the stability and reliability of these findings.

## Discussion

### Cross-validation–based learning challenges conventional statistical approach for investigating specific lateralization

Traditional neuroscience studies commonly assume that results from within-group analyses are specific to the group being studied [41–43]. While using only male or female data to explore sex-specific characteristics may seem intuitive, our cross-validation results challenge this assumption. For example, as shown in Fig. 2A, standard logistic regression models trained exclusively on data from a single target group (male or female) achieved nearly identical performance on test sets from both the target and nontarget groups. Thus, these baseline models’ performance appears insensitive to sex-based sampling. According to statistical learning theory [51], the similarity in generalization errors indicates that these models capture general patterns applicable to both males and females, rather than being sex-specific. This finding implies that statistical modeling can learn common patterns even using data from a specific group, contradicting the conventional assumption in group-specific analysis. This conclusion holds at least in our study on left versus right brain classification using the HCP and GSP datasets.

In contrast, the performance of our sex-specific models (GSDA with $\lambda >0$) is sensitive to sex-based sampling. The classification results (Fig. 2A, Supplementary Figs. S1A, C and S2A, C) reveal that the generalization error for the target group test sets is significantly lower than that for the nontarget group test sets. This indicates a stronger specificity to sex compared to the multivariate baseline models, as reflected by our GSI results. The differences observed in test performance highlight the importance of cross-validation in validating the group specificity of statistical analysis results.

While univariate analysis results are not directly applicable to unseen samples for testing, the strong correlation between univariate and multivariate baselines offers valuable insights. For example, the correlation of the within-group univariate $t$-test results with univariate control models (mixed) and multivariate baselines exceeds 0.99 and 0.91, respectively. This suggests that the lateralization patterns from the $t$-values of our within-group univariate analyses are likely common to both males and females. Consequently, previous conclusions from such within-group analyses should be revisited and revalidated. Moreover, although multivariate methods are theoretically better at capturing interactions between features than univariate methods, the observed similarity in results suggests that multivariate approaches may not always reveal patterns beyond those identified by univariate analyses.

### Sex-specific lateralized regions and connections across datasets

The mask resulting from the second-order classification revealed sex differences in connections across lobes, including the frontal, temporal, parietal, insular, limbic, and occipital lobes, where the functional differences between males and females were observed in previous studies [10, 11, 52–54]. Among the sex-specific lateralized connections, MTG-IPL, MFG–inferior frontal gyrus (IFG), and MFG-INS are shared in both male- and female-specific models across the 2 datasets.

*From the perspective of the gyrus*, which engages in various cognitive functions, the lateralized regions include the MTG [55] (sound recognition and language processing), MFG [56] (literacy and numeracy), IPL [57, 58] (spatial attention, multimodal sensory integration, and oculomotor control), IFG [59] (speech and language processing), and INS [60] (various sensorimotor processing and risk–reward behavior). These regions show lateralization and sex differences in certain functions, including speech processing, language, and spatial attention [10, 12, 52]. The MFG, a hub region in this study, is a core component of the multiple demand system [61] and presents hemispheric specialization, with the left MFG primarily supporting literacy development, while the right MFG is vital for numeracy [56]. The MTG showed lateralization in activated volumes for both males and females during language tasks, while the lateralization of IFG was only observed in males [62]. Our study reports different weights of connections related to these 2 regions. This suggests that the lateralization of a region’s external connections can reflect the lateralization of its functional activation. The “activity flow” theory in neuroscience has linked the connections and functional activation [63], proposing that the seed-based connection-weighted sums of the activation of other regions can predict functional activation of the seed region. Our results suggest *a correlation between the lateralization of functional connectivity and activation*, although further quantitative analysis is required to investigate the specifics of this relationship.

*From the perspective of connections*, our experimental results identified 3 sex-specific lateralized connections shared by males and females across datasets: MTG-IPL, MFG-IFG, and MFG-INS, aligning with established functional and structural mechanisms underlying sex differences in cognitive processing. The MTG-IPL connection is associated with language and picture-naming tasks, highlighting its role in integrating sensory and semantic information [64]. Studies have demonstrated notable plasticity in this connection, alongside sex differences in picture naming speed [65] and reliably left-lateralized evoked activations during picture-naming tasks [66]. Our GSDA framework captured these sex and hemispheric differences, suggesting that lateralization in picture naming might be driven by related connectivity, such as MTG-IPL. Variations in lateralization may be attributed to differences in the adaptive neurobiological functions of regions such as the orbitofrontal cortex (OFC), known to modulate language processing and potentially influence connectivity patterns [67].

Regarding the MFG-IFG connection, proficiency in processing artificial grammar rules has been positively associated with functional connectivity between the left IFG and left MFG, highlighting sex-related variations in these cognitive functions [62, 68, 69]. Consistent with these findings, our framework identified MFG-IFG as a sex-specific lateralized connection. Moreover, this connection is linked with processing concessive and causal relationships, with differential effective connectivity patterns potentially modulated by sex-specific functional gradients within the inferior frontal gyrus (IFG) [70, 71]. Structural and functional variations within the dorsal attention network (DAN), particularly involving MTG, IPL, and the superior parietal lobule (SPL), further illustrate how these neural connections may adapt differently between males and females [72]. Additionally, meta-analytic evidence indicates lateralization differences within IFG subdivisions, with the left IFG supporting classical language networks and the right IFG engaged in broader cognitive control [73].

The MFG-INS connection is associated with mild traumatic brain injury (mTBI) [74]. Our experimental findings underscore the need for personalized diagnostic and intervention strategies that consider both hemispheric and sex differences to enhance the effectiveness of mTBI treatment.

Collectively, these results suggest that sex-specific lateralization emerges from intricate interactions among structural connectivity gradients, functional network dynamics, and adaptive neurobiological mechanisms.

### Sex differences: lateralization strength and lobe-level interactions

*Sex differences in the strengths of shared lateralized connections*: As reported in the Results section, the first-order weights corresponding to these shared connections show consistent sex differences (Fig. 6A–D). In our labeling strategy, “left” was labeled as 0 and “right” as 1. Therefore, a positive first-order weight indicates that stronger positive interactions (FC value approaching 1) between 2 regions of interest (ROIs) suggest a higher probability of right lateralization. Conversely, a more negative interaction (FC value approaching −1) indicates that stronger negative interactions between 2 ROIs suggest a higher probability of left lateralization. The opposite interpretation applies to the negative first-order weights. Therefore, we can interpret the sex differences in the first-order weights for the shared connections as follows: *positive interactions involving the frontal lobes are more right-lateralized in females than in males, whereas positive interactions involving the temporal, parietal, insular, and limbic lobes are more left-lateralized in males than in females*. This observation of difference in strength of lateralization aligns with the findings on the lateralization patterns of right- and left-handed individuals [75] and supports neuroscience discoveries of shared functional network mechanisms across males and females [43, 76].

*Sex differences in inter-/intralobe interaction patterns are identified by the “exclusive” lateralized connections*, particularly within the frontal lobe (Fig. 6E–H), a key region for language processing [77]. Using the same approach as above for interpreting first-order weights, we can summarize that *males have a stronger left lateralization in positive interlobe interactions, while females have a stronger right lateralization in positive intraobe interactions*. This divergence may result from the evolutionary pressure for lateralization, which optimizes functional organization and reduces redundancy among brain regions [78]. Interlobe connections, characterized by long-range wiring, are metabolically costly [6, 79–81], while the shorter-range intralobe connections are more energy-efficient. These intrafrontal connections may enhance language abilities in females. Conversely, male interlobe connections may be driven by the need to engage more extensive functional areas for complex visuospatial tasks. This divergence may contribute to sex differences in cognitive abilities, with males typically excelling in rightward visuospatial tasks and females in leftward verbal tasks [3, 82].

The observed sex differences in intra- and interlobe lateralized connectivity may be attributed to a combination of genetic, hormonal, and structural factors [7, 12, 53, 83, 84], which were discussed in the Background section. Estrogen in females enhances local synaptic plasticity and dendritic arborization, particularly in frontal regions, favoring specialized intralobe networks, while testosterone in males promotes long-range axonal growth and interlobe integration [13, 53, 83, 84]. Genetic factors, such as X-chromosome-linked genes, may further refine female-pattern intralobe connectivity through synapse regulation, whereas sex-specific epigenetic and neurodevelopmental pathways could prioritize cross-regional wiring in males [84, 85]. Developmental synaptic pruning also differs: females retain more intralobe connections due to slower or later pruning, preserving modular processing, while males undergo earlier pruning to streamline interlobe efficiency. Structurally, higher gray matter density in female frontal lobes supports localized processing, whereas males exhibit greater long-range white matter tracts (e.g., superior longitudinal fasciculus) for interlobe communication [53, 79, 80, 83, 84]. Evolutionary pressures may have reinforced these patterns, with intralobe specialization in females aligning with social and detail-oriented tasks, and interlobe integration in males supporting spatial-motor coordination. These mechanisms, while probabilistic, interact with environmental and experiential factors, contributing to sex-specific cognitive strengths and vulnerabilities [86, 87].

### Limitations

Our classification models were trained and tested within each dataset. To preserve meaningful features and explore whether consistent patterns could be identified across datasets, we did not apply harmonization. While our approach maintained the integrity of the original features, future research incorporating harmonized data and cross-dataset generalization could provide additional validation to further enhance the robustness and reliability of our findings.

## Potential implications

Our study focused on sex as a grouping factor and utilized brain hemisphere labels to identify sex-specific lateralized patterns within human brain functional networks. The results demonstrate efficacy and stability in identifying and validating sex differences in lateralization. Importantly, the scope of this general predictive framework extends beyond its current application. One future direction for our proposed dual-classification workflow involves predicting group-specific prognosis and treatment outcomes (e.g., changes in clinical, cognitive, or behavioral ratings) in psychiatric disorders, considering essential covariates such as age and sex [88, 89]. Previous studies, such as Chopra et al. [88], have aimed to minimize the impact of these covariates to enhance generalizability in predictive modeling. In contrast, our dual-classification approach provides a complementary method for identifying biomarkers or predicting treatment responses specific to particular covariates, thus supporting precision medicine initiatives. Furthermore, our dual-classification framework extends beyond sex-specific analyses and holds potential for uncovering subtype-specific neuroimaging biomarkers, offering valuable insights into personalized prognosis and treatment strategies across various psychiatric disorders [90]. Moreover, the GSDA algorithm can be adapted to combinations of grouping factors, such as sex and age groups. While this work primarily focused on classification, our method is adaptable to regression tasks, such as predicting behavioral cognitive scores or drug dosages.

## Methods

### Dual-classification with GSDA

We propose a dual-classification framework with 2 primary objectives: learning group-specific models and identifying group-specific discriminant weights. For the first objective, specifically the classification of left versus right brain hemispheres, we train a linear classifier on the training data and then validate its performance on the test data. We refer to this process as the **first-order classification**. The weights derived from the model are called the **first-order weights**. Then we perform a second round of classification to identify the weights that show significant differences between group-specific models. Here, we train a linear classifier to differentiate between male- and female-specific models. This stage is called the **second-order classification**, and the associated weights are referred to as the **second-order weights**. This process is illustrated in stages ⑤ and ⑥ of Fig. 1A.

The first-order classification builds a (group-specific) prediction function. This function predicts whether an unseen brain hemisphere is left or right, based on a feature vector. These vectors represent the left or right human brain hemispheres and are extracted from the training neuroimaging data. The resulting prediction accuracy serves as a quantitative measure of the extent to which the learned lateralization patterns are generalized among the brain networks within the test set. The learned model weights can be interpreted as indicators of the significance or extent of differences between the corresponding connections of the left and right brain hemispheres.

The second-order classification is designed to identify weights that show significant differences between the male- and female-specific first-order models. In this stage, a linear classification model is trained on the first-order model weights to predict whether an unseen model is male- or female-specific. The features with larger weights in the second-order classification are considered to represent the stronger sex differences.

To learn group-specific models for the first-order classification, we propose a GSDA algorithm.

#### Problem formulation of GSDA

Let $(\mathbf {x}_i, y_i, \mathbf {g}_i)$ represent the $i$th sample, where $\mathbf {x}_i \in \mathcal {X} \subseteq \mathbb {R}^p$ denotes an input data vector, $y_i \in \mathcal {Y}$ denotes an output variable (label), and $\mathbf {g}_i \in \mathcal {G} \subseteq \mathbb {R}^q$ represents a covariate vector for the grouping factor(s). Here, $i \in [1, m]$, with $m$ being the total number of samples. $\mathcal {X}$, $\mathcal {Y}$, and $\mathcal {G}$ are the feature spaces of the input data, output label, and grouping factor, respectively, with $p$ and $q$ as the corresponding feature dimensions for the input data $\mathbf {x}_i$ and grouping factor $\mathbf {g}_i$. In the context of this article, $\mathbf {x}_i$ is a feature vector that represents a brain hemisphere, $y_i$ indicates whether $\mathbf {x}_i$ is the left or right hemisphere, and $\mathbf {g}_i$ is a binary (0 and 1) indicator representing whether $\mathbf {x}_i$ is from a male or female subject (e.g., $g_i = 0$ for male and $g_i = 1$ for female). Assuming $\mathbf {x}_0 = 1$, considering $\mathbf {w}_0$ as the bias term, and denoting $\mathbf {w} \in \mathbb {R}^{p+1}$ as the vector of weights (coefficients) to be learned, with the target group represented as subscript $_\mathrm{t}$, we formulate the objective of learning group-specific models as follows:


(1) \begin{eqnarray*}
\underset{\mathbf w}{\text{arg max}} \frac{1}{m_\mathrm{t}}\sum _{i=1}^{m_\mathrm{t}} \mathbb {P}(y_i|\mathbf {x}_i, \mathbf {w}) + \frac{\lambda }{m}\sum _{j=1}^{m} \big |\mathbb {P}(\mathbf {g}_j, \mathbf {w}^\top \mathbf {x}_j) - \mathbb {P}(\mathbf {g}_j)\mathbb {P}(\mathbf {w}^\top \mathbf {x}_j) \big |,
\end{eqnarray*}

where $m_\mathrm{t}$ denotes the number of training samples from the target group, and $\lambda \ge 0$ is the hyperparameter that quantifies the importance of grouping factor(s) dependence. Based on Equation (1), we formulate a general GSDA framework as


(2) \begin{eqnarray*}
\underset{\mathbf w}{\text{arg min}} L(\mathbf {X}_{\mathrm{t}}^\top \mathbf {w},\mathbf {y}_{\mathrm{t}}) + \alpha \Vert \mathbf {w}\Vert _K^2 - \lambda \underbrace{\rho (\mathbf {X}^\top \mathbf {w}, \mathbf {G})}_{\text{Group dependence}},
\end{eqnarray*}

where $L(\cdot , \cdot )$ denotes a classification or regression loss function, such as least square, logistic, or hinge; $\alpha \ge 0$ is the hyperparameter used for weight regularization; $\Vert \cdot \Vert _K^2$ denotes either an $\ell _1$ or $\ell _2$ regularization, with $K=1$ or 2, respectively; $\mathbf {X}_{\mathrm{t}}$ denotes the target group’s training samples; $\mathbf {X}$ denotes all training samples that consist of both target and nontarget group samples; and $\rho (\cdot , \cdot )$ is a statistical dependence measure. In this work, we employed the Hilbert–Schmidt independence criterion (HSIC) [91], a convex and smooth dependence measure. Given 2 sets $\mathbf {X}=\lbrace \mathbf {x}_1, \mathbf {x}_2, \dots , \mathbf {x}_m\rbrace$ and $\mathbf {Y}=\lbrace \mathbf {y}_1, \mathbf {y}_2,\dots , \mathbf {y}_m\rbrace$, both with size $m$, HSIC computes the statistical dependence between tests, whether $\mathbf {X}$ and $\mathbf {Y}$, via


(3) \begin{eqnarray*}
\rho _h(\mathbf {X, Y}) = \frac{1}{(m-1)^{2}}\text{tr}(\mathbf {KHLH}),
\end{eqnarray*}

where $\mathbf {K, H, L} \in \mathbb {R}^{m\times m}$, $\mathbf {K}_{i,j}:= k_x(\mathbf {x}_i, \mathbf {x}_j)$, $\mathbf {L}_{i,j}:= k_y(\mathbf {y}_i, \mathbf {y}_j)$, $k_x(\cdot , \cdot )$, and $k_y(\cdot , \cdot )$ are 2 kernel functions, such as linear, polynomial, or radial basis function (RBF); $\mathbf {H} = \mathbf {I} -\frac{1}{m}\mathbf {11^\top }$ is the centering matrix; $\mathbf {I}$ is an identity matrix; and $\text{tr}\left(\cdot \right)$ is the trace function. HSIC $\rho (\mathbf {X, Y})\ge 0$, and it is zero if and only if the 2 sets of variables $\mathbf {X}$ and $\mathbf {Y}$ are independent, that is, $\mathbb {P}(\mathbf {x, y}) = \mathbb {P}(\mathbf {x})\mathbb {P}(\mathbf {y})$. A higher HSIC value suggests stronger statistical dependence.

#### GSDA with logistic loss and maximum likelihood estimation

To maximize the likelihood of the target group labels and the grouping factor(s) dependence as specified in Eq. (1), we adopt maximum likelihood estimation for optimizing the model weights $\mathbf {w}$. Here, we develop a novel algorithm, GSDA with logistic loss (GSDA-Logit), as a variant of logistic regression for group-dependent learning. Let $\mathbb {P}(\mathbf {y}_{\mathrm{t}}|\mathbf {X}_{\mathrm{t}}, \mathbf {w})$ denote the likelihood of target labels $\mathbf {y}_{\mathrm{t}}$ given the model and target group data $\mathbf {X}_{\mathrm{t}}$; $\mathbb {P}(\mathbf {w})$ be the prior probability of weights, assumed to follow a normal distribution $\mathcal {N}(0, \sigma ^2)$; and $\mathbb {P}(\rho (\mathbf {X}^\top \mathbf {w}, \mathbf {G}))$ be the likelihood of grouping factor dependence. The overall likelihood $\mathcal {L}(\mathbf {w})$ to be maximized is as follows:


(4) \begin{eqnarray*}
\begin{aligned} \mathcal {L}(\mathbf {w}) & =\mathbb {P}(\mathbf {y}_{\mathrm{t}}|\mathbf {X}_{\mathrm{t}},\mathbf {w})\mathbb {P}(\mathbf {w})\mathbb {P}(\rho (\mathbf {X}^\top \mathbf {w}, \mathbf {G})) \\
&= \left(\prod _{i=1}^{m_{\mathrm{t}}} S(\mathbf {w}^\top \mathbf {x}_i)^{y_i} \left(1 - S(\mathbf {w}^\top \mathbf {x}_i)\right)^{(1 - y_i)} \right) \\
&\quad \times \frac{1}{\sqrt{2\pi \sigma }} \exp \left(\!-\frac{\mathbf {w}^\top \mathbf {w}}{2\sigma ^2}\right) S\left(\rho _h(\mathbf {w}^\top \mathbf {X}, \mathbf {G})\right), \end{aligned}
\end{eqnarray*}

where $S(\cdot )$ denotes the logistic (or sigmoid) function, and $\mathbb {P}(\mathbf {w})$ can be interpreted as the $\ell _2$ regularization for $\mathbf {w}$. Given that $\mathbf {w^\top X}$ produces a row vector, Equation (3) can be reformulated as simplified HSIC [92]:


(5) \begin{eqnarray*}
\begin{aligned} \rho _{sh}(\mathbf {w^\top X}, \mathbf {G}) &= \text{tr}((\mathbf {w^\top X})^\top (\mathbf {w^\top X})\mathbf {HL}\mathbf {H}) \\
&= \mathbf {w}^\top \mathbf {XHLHX}^\top \mathbf {w}, \end{aligned}
\end{eqnarray*}

where $\mathbf {L} = \mathbf {G^\top G}$. By replacing $\rho _h(\mathbf {w^\top X}, \mathbf {G})$ with the simplified HSIC $\rho _{sh}(\mathbf {w^\top X}, \mathbf {G})$, the likelihood can be rewritten as


(6) \begin{eqnarray*}
\begin{aligned} \mathcal {L}(\mathbf {w}) &= \left(\prod _{i=1}^{m_{\mathrm{t}}} S(\mathbf {w}^\top \mathbf {x}_i)^{y_i} \left(1 - S(\mathbf {w}^\top \mathbf {x}_i)\right)^{(1 - y_i)}\right) \\
&\quad \times \frac{1}{\sqrt{2\pi \sigma }} \exp \left(- \frac{\mathbf {w}^\top \mathbf {w}}{2\sigma ^2}\right) S\left(\rho _{sh}(\mathbf {w}^\top \mathbf {X}, \mathbf {G})\right). \end{aligned}
\end{eqnarray*}

The likelihood in Equation (6) can be maximized using the same optimization steps for a standard logistic regression (i.e., computing the gradient of the negative log-likelihood). Let $\alpha = \frac{1}{\sigma ^2}$ and $\lambda$ denote the 2 hyperparameters that control the importance of the $\ell _2$ regularization and grouping factor dependence regularization, respectively. Let $\mathcal {J}(\mathbf {w})$ denote the negative logarithm of the likelihood. Taking the gradient of $\mathcal {J}(\mathbf {w})$ with respect to $\mathbf {w}$, we obtain


(7) \begin{eqnarray*}
\begin{aligned} \nabla \mathcal {J}(\mathbf {w}) =\; & \mathbf {X}_{\mathrm{t}}(S(\mathbf {X}^\top _{\mathrm{t}}\mathbf {w}) - \mathbf {y}_{\mathrm{t}}) + \alpha \mathbf {w} \\
& + \lambda (S(\rho _{sh}(\mathbf {w^\top X}, \mathbf {G})) - 1)\mathbf {XHLHX^\top w}. \end{aligned}
\end{eqnarray*}

Finally, $\mathbf {w}$ can be optimized iteratively via


(8) \begin{eqnarray*}
\mathbf {w}^{k+1} = \mathbf {w}^k - \eta \nabla \mathcal {J}(\mathbf {w}^k),
\end{eqnarray*}

where $k$ denotes the $k$th iteration, and $\eta$ is the learning rate (step size). Algorithm 1 is the pseudocode for GSDA-Logit. In addition to standard gradient descent optimization, we have implemented the LBFGS algorithm [93] for faster optimization.

**Algorithm 1:**
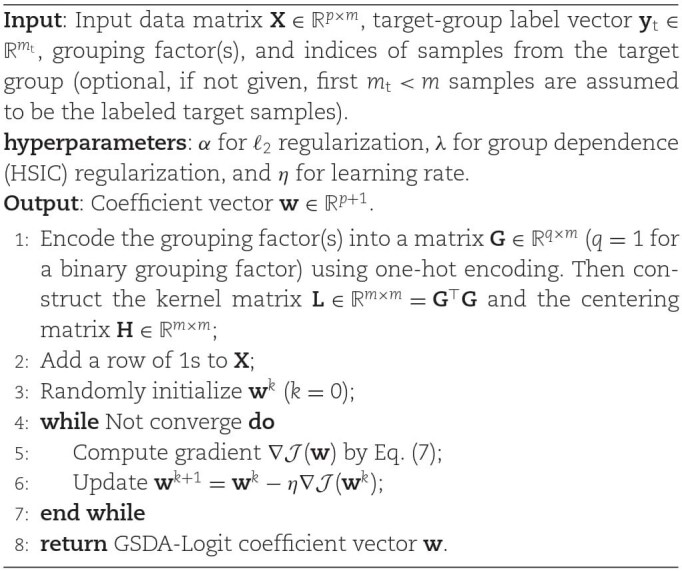
Group-Specific Discriminant Analysis with logistic loss (GSDA-Logit)

#### Theoretical interpretation for diverged test accuracy between groups

Here, we provide a theoretical analysis to interpret the accuracy divergence in Fig. 2A of a GSDA model on target group and nontarget group data. Let $h \in \mathcal {H}$ be a hypothesis for predicting label $y$, where $\mathcal {H}$ is the hypothesis space. In the context of linear models with logistic loss in this article, a hypothesis $h(\mathbf {x})$ is defined as


(9) \begin{eqnarray*}
h(\mathbf {x}) = \left\lbrace \begin{array}{@{}l@{\quad }l@{}}1 \text{, if } S(\mathbf {w^\top x}) \ge 0.5,\\
0 \text{, otherwise.} \end{array}\right.
\end{eqnarray*}

Let the target group data be drawn from the distribution $\mathcal {D}_{\mathrm{t}}$; then, by a standard application of Vapnik–Chervonenkis (VC) theory [51], the bound on the generalization error for target group data is


(10) \begin{eqnarray*}
\epsilon _{\mathrm{t}}(h) \le \hat{\epsilon }_{\mathrm{t}}(h) + \mathrm{O}\bigg (\sqrt{\frac{d}{m}\ln \frac{m}{d}}\bigg ),
\end{eqnarray*}

where $\epsilon _{\mathrm{t}}(h)$ denotes the generalization error for $h$ on the target group data, $\hat{\epsilon }_{\mathrm{t}}(h)$ is the empirical generalization error for $h$ on the target group training examples, $d$ represents the VC dimension [51] of the hypothesis space $\mathcal {H}$, and $\mathrm{O}(\cdot )$ denotes computational complexity. According to the domain adaptation theory [94], the upper bound on the generalization error for samples from the nontarget group(s) is


(11) \begin{eqnarray*}
\epsilon _{\mathrm{\setminus t}}(h) \le \hat{\epsilon }_{\mathrm{t}}(h) + \mathrm{O}\bigg (\sqrt{\frac{d}{m}\ln \frac{m}{d}}\bigg ) + d_{\mathcal {H}}(\mathcal {D}_{\mathrm{t}}, \mathcal {D}_{\mathrm{\setminus t}}) + \Omega ,
\end{eqnarray*}

where $\mathcal {D}_{\mathrm{\setminus t}}$ represents the distribution for nontarget group data, and $\Omega = \epsilon _{\mathrm{t}}(h^{*}) + \epsilon _{\mathrm{\setminus t}}(h^{*})$, with $h^{*} = \text{arg min }_{h \in \mathcal {H}}\epsilon _{\mathrm{t}}(h) + \epsilon _{\mathrm{\setminus t}}(h)$ being the *ideal joint hypothesis* for target group and nontarget group data. $\Omega$ is a constant for fixed data and can be zero if $h^{*}$ can accurately predict any sample from both $\mathcal {D}_{\mathrm{t}}$ and $\mathcal {D}_{\mathrm{\setminus t}}$. $d_{\mathcal {H}}(\mathcal {D}_{\mathrm{t}}, \mathcal {D}_{\mathrm{\setminus t}})$ is the $\mathcal {H}$-divergence, which measures the divergence between the target and nontarget data distributions by


(12) \begin{eqnarray*}
d_{\mathcal {H}}(\mathcal {D}_{\mathrm{t}}, \mathcal {D}_{\mathrm{\setminus t}}) = 2 \sup _{h \in \mathcal {H}}\big |\mathbb {P}_{\mathcal {D}_{\mathrm{t}}}[I(h)] - \mathbb {P}_{\mathcal {D}_{\mathrm{\setminus t}}}[I(h)]\big |,
\end{eqnarray*}

where $I(h)$ is an indicator function that $\mathbf {x} \in I(h) \Leftrightarrow h(\mathbf {x})=1$. Assume $g_i=1$ if $\mathbf {x}_i\in \mathcal {D}_{\mathrm{t}}$, and $g_i=0$ if $\mathbf {x}_i\in \mathcal {D}_{\mathrm{\setminus t}}$, the right-hand side of Equation (12) can be rewritten as


(13) \begin{eqnarray*}
\begin{aligned} \big | \mathbb {P}_{\mathcal {D}_{\mathrm{t}}}[I(h)]\! -\! \mathbb {P}_{\mathcal {D}_{\mathrm{\setminus t}}}[I(h)] \big | & \!= \!\big | \mathbb {P}(h(\mathbf {x}) \! = 1\! \mid \! g \!= \!1) \!-\! \mathbb {P}(h(\mathbf {x}) = 1 \!\mid \! g \!= \!0) \big |\\
& \!= \!\big | \mathbb {P}(S(\mathbf {X}_{\mathrm{t}}^\top \mathbf {w}) \!\ge \! 0.5) \! - \! \mathbb {P}(S(\mathbf {X}_{\mathrm{\setminus t}}^\top \mathbf {w})\! \ge \!0.5) \big |, \end{aligned}
\end{eqnarray*}

which can be viewed as the separability of transformed data $\mathbf {X}_{\mathrm{t}}^\top \mathbf {w}$ and $\mathbf {X}_{\mathrm{\setminus t}}^\top \mathbf {w}$ in the linear model context.

If we view the objective of maximizing dependence between $\mathbf {g}$ and $\mathbf {X}^\top \mathbf {w}$ in Equation (1) as maximizing the corresponding mutual information, GSDA minimizes the uncertainty about the group labels $\mathbf {g}$ given the transformed data $\mathbf {X}^\top \mathbf {w}$. This enhances the separability of transformed data $\mathbf {X}_{\mathrm{t}}^\top \mathbf {w}$ and $\mathbf {X}_{\mathrm{\setminus t}}^\top \mathbf {w}$. From this perspective, we interpret group dependence as a form of regularization on the hypothesis space $\mathcal {H}$, ensuring that for any $h \in \mathcal {H}$, the transformed target group and nontarget group data are separable. This results in a maximized $\mathcal {H}$-divergence in the generalization error bound for nontarget group data in Equation (11). We can see that the difference between the generalization error bounds for target group (Equation (10)) and nontarget group data (Equation (11)) is the $\mathcal {H}$-divergence term plus a constant. This directly translates to a lower generalization error bound or higher accuracy for target group data over nontarget group data in the GSDA framework. Consequently, a theoretical gap exists between the expected accuracy achieved by a GSDA model on target group and nontarget group data, with the expected accuracy for target group data being higher (i.e., GSDA models are more target-specific).

#### GSI for evaluating model group specificity

To measure the group specificity of discriminative models, we set the following criteria for a metric:

- Its value lies within $[0, 1]$.
- Its value equals 0 if the test accuracy for the target and nontarget groups is identical.
- When the test accuracy of the target and nontarget groups differs, the value of this metric should be proportional to (i) the absolute accuracy for the target group and (ii) the closeness of accuracy for the nontarget group to the random chance.
- Greater relative accuracy divergence between target and nontarget groups will result in a higher value of this metric.

To satisfy the above conditions, we propose a GSI for binary classification problems as follows:


(14) \begin{eqnarray*}
\mathrm{GSI} = 2\mathrm{BAT}(\mathrm{BAT} - 0.5 - |\mathrm{BANT} -0.5| ),
\end{eqnarray*}

where $\mathrm{BAT} \in [0.5, 1]$ and $\mathrm{BANT} \in [0, 1]$ represent the balanced accuracy of the target group and nontarget group data, respectively. Balanced accuracy is chosen to mitigate the impact of imbalanced samples. It is defined as $\mathrm{BA} = (\mathrm{TPR} + \mathrm{TNR}) / {2}$, where the true-positive rate $\mathrm{TPR} = \lbrace \text{number\,\, of\,\, true\,\, positives}\rbrace /\lbrace \text{number\,\, of\,\, total\,\, positives}\rbrace$ and the true-negative rate $\mathrm{TNR} = \lbrace \text{number of true negatives}\rbrace /$  $\lbrace \text{number of total negatives}\rbrace$. In the left versus right brain hemisphere classification problem, the numbers of left and right training examples are equal, making balanced accuracy equivalent to accuracy. The expression $|\mathrm{BANT} - 0.5|$ measures how close the accuracy of the nontarget group is to random chance (0.5), and $\mathrm{BAT} - 0.5 - |\mathrm{BANT} - 0.5|$ quantifies the relative accuracy divergence between the target and nontarget groups. Since we are interested in generalized lateralization patterns for the target group, models that perform worse than random chance on target test sets are not considered.

### Resting-state fMRI data and processing

We use resting-state fMRI data from the HCP [45] and the GSP [46] for brain hemisphere classification to study lateralization. Table 2 summarizes the demographic information of the subjects involved in our experiments across both datasets.

**Table 2:** Information of HCP and GSP datasets used for the experiments, where “M” denotes male and “F” denotes female for sex, “L” denotes left-handedness, “R” denotes right-handedness, “A” denotes ambidexterity for handedness, and “SD” denotes standard deviation.

Dataset	No. of subjects	Sex (M/F)	Handedness (L/R/A)	Average age (SD)			No. of sessions
				Total	Male	Female	
HCP [45]	960	445/515	85/875/0	28.7 (3.71)	27.9 (3.69)	29.4 (3.59)	2
GSP [46]	1,570	665/905	110/1,449/11	21.5 (2.89)	21.6 (3.04)	21.5 (2.78)	1

#### HCP

##### Acquisition

All MRI data were collected using the same 3T Siemens Skyra magnetic resonance machines at Washington University in St. Louis with a 32-channel head coil [95]. Specifically, rs-fMRI was acquired using a gradient-echo echo-planar imaging (GE-EPI) sequence with the following parameters: repetition time (TR) = 720 ms, echo time (TE) = 33.1 ms, flip angle (FA) = 52$^{\circ }$, bandwidth = 2,290 Hz/pixel, field of view (FOV) $= 208 \times 180 \text{ mm}^2$, matrix $= 104 \times 90$, voxel size $= 2 \times 2 \times 2 \text{ mm}^3$, multiband acceleration factor $= 8$, slices = 72, and total scan time of 1,200 frames = 14 minutes and 24 seconds [45]. During the scan, participants were asked to open their eyes and stare at a white cross on a screen with a black background. There were 2 rs-fMRI sessions (REST1 and REST2) acquired on 2 consecutive days, each including 2 runs with a left-to-right (LR) and a right-to-left (RL) phase encoding direction. The T1-weighted images were acquired by using a magnetized rapid gradient-echo imaging (MPRAGE) sequence with the following parameters: TR = 2,400 ms, TE = 2.14 ms, reversal time (TI) = 1,000 ms, FA = 8$^{\circ }$, FOV $= 224 \times 224 \text{ mm}^2$, voxel size 0.7 mm isotropic, and total scan time = 7 minutes and 40 seconds.

##### Preprocessing

We follow the same steps in [96] for HCP data preprocessing. The HCP minimal preprocessing pipeline (version 2.0) was utilized, including magnetic gradient distortion correction, EPI distortion correction, non–brain tissue removal, Montreal Neurological Institute (MNI) standard space registration, and intensity normalization. The resultant data were denoised using independent component analysis (ICA) with the FIX tool [97], which identifies and eliminates spatiotemporal signal components from nonneuronal or structural noise, with an emphasis on head movement. Subsequently, 5 postprocessing steps were applied to the minimally preprocessed data: (i) spatial smoothing with a 4-mm full width at half maximum (FWHM) kernel, twice the voxel resolution of HCP fMRI data; (ii) linear detrending to minimize the effects of low-frequency drift; (iii) regression of a suite of nuisance variables unrelated to neural signals, such as average signals from white matter (WM) and cerebrospinal fluid (CSF), as well as the whole brain (global signal, GS); (iv) bandpass filtering (0.01–0.1 Hz); and (v) scrubbing to control effects of transient movement across the time-series frames.

#### GSP

##### Acquisition

All imaging data were collected on matched 3T Tim Trio scanners (Siemens Healthcare) at Harvard University and Massachusetts General Hospital using the vendor-supplied 12-channel phased-array head coil [46]. Structural data included a high-resolution (1.2-mm isotropic) multiecho T1-weighted magnetization-prepared gradient-echo image. Functional imaging data were acquired using a GE-EPI sequence sensitive to blood oxygenation level-dependent (BOLD) contrast with the following parameters: TR = 3,000 ms, TE = 30 ms, FA = 85$^{\circ }$, voxel size $= 3 \times 3 \times 3 \text{ mm}^3$, slices = 47, and total scan time of 124 frames = 6 minutes and 12 seconds.

##### Preprocessing

Same as for HCP data preprocessing, we follow [96] to preprocess GSP data. SPM preprocessed all fMRI data [98] and GRETNA [99] toolkit, including the following steps: (i) removing the first 4 volumes to ensure that the magnetization is at steady state; (ii) slice-timing correction; (iii) realignment of all volumes to the first volume to reduce the effects of head motion; (iv) co-registration of GE-EPI data to the native, cropped, high-resolution structural image and then normalizing them to the MNI space through the Diffeomorphic Anatomical Registration Through Exponentiated Lie Algebra (DARTEL) algorithm; (v) spatial smoothing with a 6-$mm$ FWHM kernel, twice the voxel resolution of GSP fMRI data; (vi) linear detrending to minimize the effects of low-frequency drift; (vii) 6 head motion parameter regression, as well as the WM, CSF, and GS; and (viii) low-pass filtering ($<$0.08 Hz) [100].

#### Extracting intrahemispheric brain network

We use intrahemispheric brain network connectivity as features to represent brain hemispheres. Fig. 1A ①–③ illustrates the data-processing workflow for obtaining intrahemispheric connections from resting-state time series. Time sequences were extracted using the BNA [50], which divides the human brain into 246 regions (123 per hemisphere). Pearson correlation was computed to represent the connectivity between brain regions. Following Liang et al. [101], the correlation coefficients were transformed into $z$-scores using Fisher’s $z$ transform. For HCP data, we averaged $z$-scores across the RL and LR runs for each session. To extract half-brain features, we reordered the columns and rows of the connectivity matrix to produce two 123 $\times$ 123 matrices, representing the intrahemispheric networks for the 2 brain hemispheres of each subject. We then extracted the upper triangle of these matrices (illustrated as the red and blue areas in ③ of Fig. 1) to form two 7,503-dimensional feature vectors by BNA for the 2 hemispheres for experiments.

### Experimental setting

#### Multivariate classification algorithm setup

For all multivariate methods, the classification problem is binary: left brain hemispheres are labeled as 0, and right brain hemispheres are labeled as 1. For GSDA-Logit, sex is utilized as the grouping factor in the experiments, encoding males as 0 and females as 1. Given the binary nature of the grouping factor, the matrix $\mathbf {G}$ simplifies to a vector $\mathbf {g}$ in this experiment. For first-order classification with group-specific model training, we set the regularization parameter $\alpha =0.1$ and varied $\lambda \in [0, 1.0, 2.0, 5.0, 8.0, 10.0]$ for GSDA-Logit, where $\alpha$ controls $\ell _2$ regularization, and $\lambda$ regulates statistical dependence on grouping factors. When $\lambda =0$, GSDA-Logit degenerates to standard logistic regression, as it does not incorporate grouping factor dependence in optimizing model weights. To learn first-order multivariate control models and conduct second-order classification, we employed a logistic regression classifier implemented in scikit-learn [102] with default hyperparameters.

#### Cross-validation strategy

##### First-order classification setting

We implemented 2 cross-validation strategies for left versus right brain hemisphere classification:

1. Within each dataset, subjects were randomly divided into 2 equal groups (50% each). The training set consisted of left hemispheres from the first group and right hemispheres from the second group, while the remaining hemispheres (right hemispheres of the first group and left hemispheres of the second group) were used for testing. This setup ensured that no subject contributed both hemispheres to the training set, minimizing potential biases from intrasubject correlations, as illustrated in ④ of Fig. 1A. The corresponding results are reported in Fig. 2 and Supplementary Fig. S2A, B.
2. To further validate our findings, we employed an alternative strategy in which 20% of subjects were held out entirely as an additional unseen test set. The training examples were obtained by applying the same strategy above to the remaining 80% of subjects.

Each cross-validation strategy was repeated 1,000 times, generating 1,000 models per learning task. For the HCP dataset, which includes 2 scanning sessions per subject on different days, the session not used for training served as an additional test set.

##### Second-order classification setting

Using the first-order models learned for each task, we perform second-order classification through the following steps:

1. Define a classification problem of interest, for example, male-specific GSDA models trained on the HCP with $\alpha =0.1$, $\lambda =5$ versus female-specific GSDA models trained on the HCP with $\alpha =0.1$, $\lambda =5$, with 1,000 models for each group.
2. Split the 2,000 models into 80% training and 20% test sets by stratified random sampling.
3. Train a standard logistic regression classifier using the scikit-learn [102] implementation with the default setting on the training set and then evaluate the performance on the test set.
4. Repeat steps 2 and 3 with different random seeds for 1,000 splits of training and test sets.

### Generalist Repository

There are additional data files hosted in Zenodo archives:

https://doi.org/10.5281/zenodo.10050233 [103]

https://doi.org/10.5281/zenodo.10050234 [104]

### GitHub Repository

The software code is available from GitHub repository: https://github.com/shuo-zhou/GSDA-Lateralization [105].

A version of record snapshot of the GitHub repository has been archived in the Software Heritage Library [106] with the PID swh:1:snp:495f818df0e3c6d9ac1898b1cc14ec0ea396d98a.

## Additional Files

**Supplementary Fig. S1**. Left versus right brain classification results using GSDA-Logit on HCP data [45], employing 2 cross-validation strategies different from the one in Fig. 2. (A) Average test accuracy on the held-out session; for example, training was conducted on the 50% hemispheres, same as in Fig. 2A, from the REST1 session, and the test was performed on the data from the REST2 session. (B) GSI calculated from the test results shown in Supplementary Fig. S1A. (C) Average test accuracy on the held-out subjects’ data; for example, training was conducted on the 80% subjects’ data sampled from the REST1 session, and test was performed on the remaining 20% subjects’ data from REST1 and REST2. (D) GSI calculated from the test results shown in Supplementary Fig. S1C. The remaining detailed descriptions of the figures, along with the main observations, are the same as those in the caption of Fig. 2.

**Supplementary Fig. S2**. Experimental results of left versus right brain classification on male and female sets from the Brain Genomics Superstruct Project (GSP) [46] using GSDA-Logit with respect to the hyperparameter $\lambda$. (A) Average test accuracy on the held-out hemispheres, with a cross-validation strategy consistent with the one in Fig. 2A. (B) GSI calculated from the test results shown in Supplementary Fig. S2A. (C) Average test accuracy on the held-out subjects’ data, with a cross-validation strategy consistent with the one in Supplementary Fig. S1C. (D) GSI calculated from the test results shown in Supplementary Fig. S2C. The remaining detailed descriptions of the figures, along with the main observations, are the same as those in the caption of Fig. 2.

**Supplementary Fig. S3**. Pearson correlation coefficients between model weights learned from GSP data [46]. (A) Correlation between male-specific and multivariate control models. (B) Correlation between female-specific and multivariate control models. (C, D) Average pairwise correlation for (C) male-specific and (D) female-specific GSDA models. The main observations are consistent with those in Fig. 3.

**Supplementary Fig. S4**. Sex-specific lateralized connections identified by (A) male-specific models for HCP (Fig. 6A + Fig. 6E), (B) female-specific models for HCP (Fig. 6B + Fig. 6F), (C) male-specific models for GSP (Fig. 6C + Fig. 6G), and (D) female-specific models for GSP (Fig. 6D + Fig. 6G).

**Supplementary Fig. S5**. Count of the group “exclusive” lateralized connections for HCP and GSP (Fig. 6E–H) categorized by associated lobes, and inter- or intralobe. The connections are identified by (A) male-specific models for HCP, (B) female-specific models for HCP, (C) male-specific models for GSP, and (D) female-specific models for GSP. (E) Sum of Supplementary Fig. S4A, C. (F) Sum of Supplementary Fig. S4B, D.

giaf082_Supplemental_File

giaf082_Authors_Response_To_Reviewer_Comments_Original_Submission

giaf082_GIGA-D-24-00330_Original_Submission

giaf082_GIGA-D-24-00330_Revision_1

giaf082_Reviewer_1_Report_Original_Submission Fei Li -- 12/13/2024

giaf082_Reviewer_2_Report_Original_Submission Guihua Jiang -- 12/27/2024

giaf082_Reviewer_2_Report_Revision_1 Guihua Jiang -- 4/13/2025

## Abbreviations

BNA: Brainnetome Atlas; BOLD: blood oxygenation level-dependent; CG: cingulate gyrus; DARTEL: Diffeomorphic Anatomical Registration Through Exponentiated Lie Algebra; FA: flip angle; FOV: field of view; GE-EPI: gradient-echo echo-planar imaging; GSDA: group-specific discriminant analysis; GSI: group specificity index; GSP: Brain Genomics Superstruct Project; HCP: Human Connectome Project; HSIC: Hilbert–Schmidt independence criterion; ICA: independent component analysis; IFG: inferior frontal gyrus; INS: insular gyrus; ITG: inferior temporal gyrus; IPL: inferior parietal lobe; OrG: orbital gyrus; PCL: paracentral lobule; Pcun: precuneus; PoG: postcentral gyrus; PrG: precentral gyrus; LOcC: lateral occipital cortex; MFG: middle frontal gyrus; MTG: middle temporal gyrus; MVOcC: medioventral occipital cortex; SFG: superior frontal gyrus; STG: superior temporal gyrus; TR: repetition time; TE: echo time; WM: white matter.

## Data Availability

All additional supporting data are available in the *GigaScience* repository, GigaDB [107].
