# Supplementary material for: Group-specific discriminant analysis enhances detection of sex differences in brain functional network lateralization
Source: Gigascience. 2025 Aug 30;14:giaf082. doi: 10.1093/gigascience/giaf082 (PMC12398281; doi:10.1093/gigascience/giaf082)
Supplement: giaf082_Supplemental_File [file giaf082_supplemental_file.pdf]

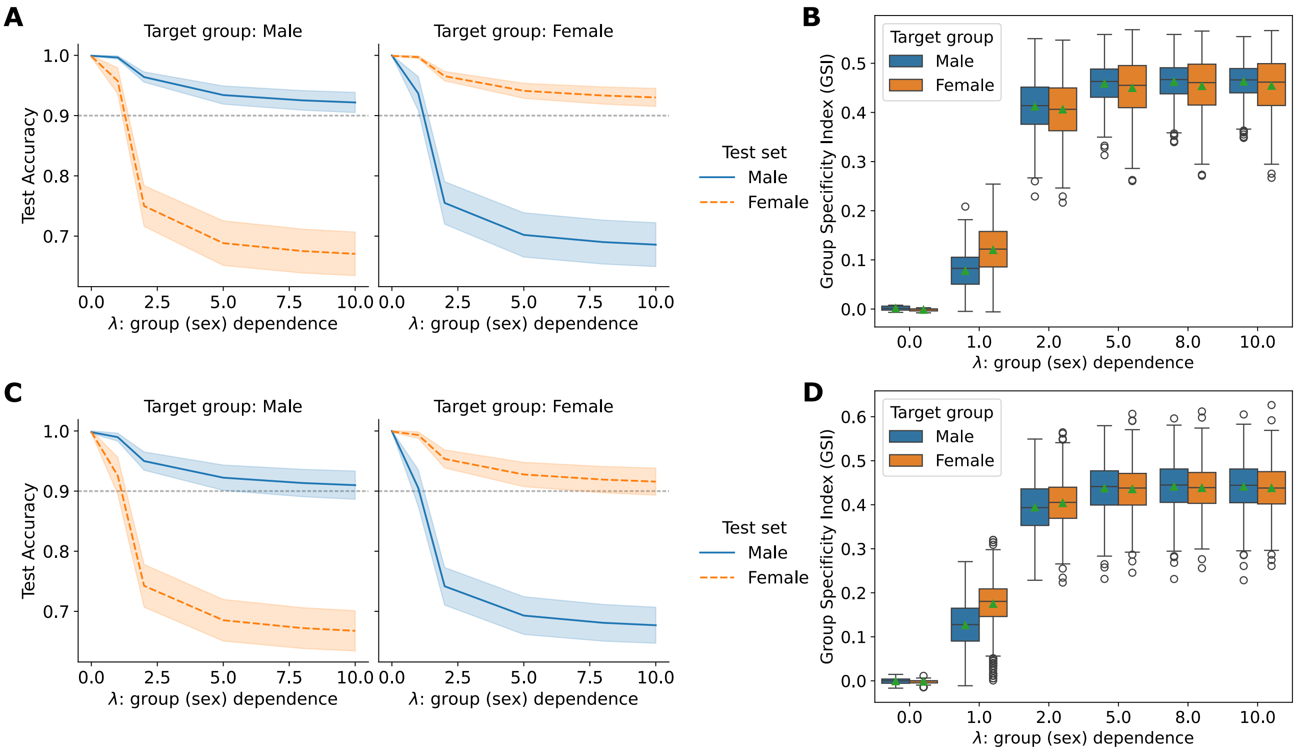

Supplementary Fig. S 1. Left vs. right brain classification results using GSDA-Logit on HCP data [45], employing two cross-validation strategies different from the one in Fig. 2. **(A)** Average test accuracy on the held-out session; for example, training was conducted on the 50% hemispheres same as in Fig. 2A from the REST1 session, and the test was performed on the data from the REST2 session. **(B)** GSI calculated from the test results shown in Supplementary Fig. S1A. **(C)** Average test accuracy on the held-out subjects' data; for example, training was conducted on the 80% subjects' data sampled from the REST1 session, and test was performed on the remaining 20% subjects' data from REST1 and REST2. **(D)** GSI calculated from the test results shown in Supplementary Fig. S1C. The remaining detailed descriptions of the figures, along with the main observations, are the same as those in the caption of Fig. 2.

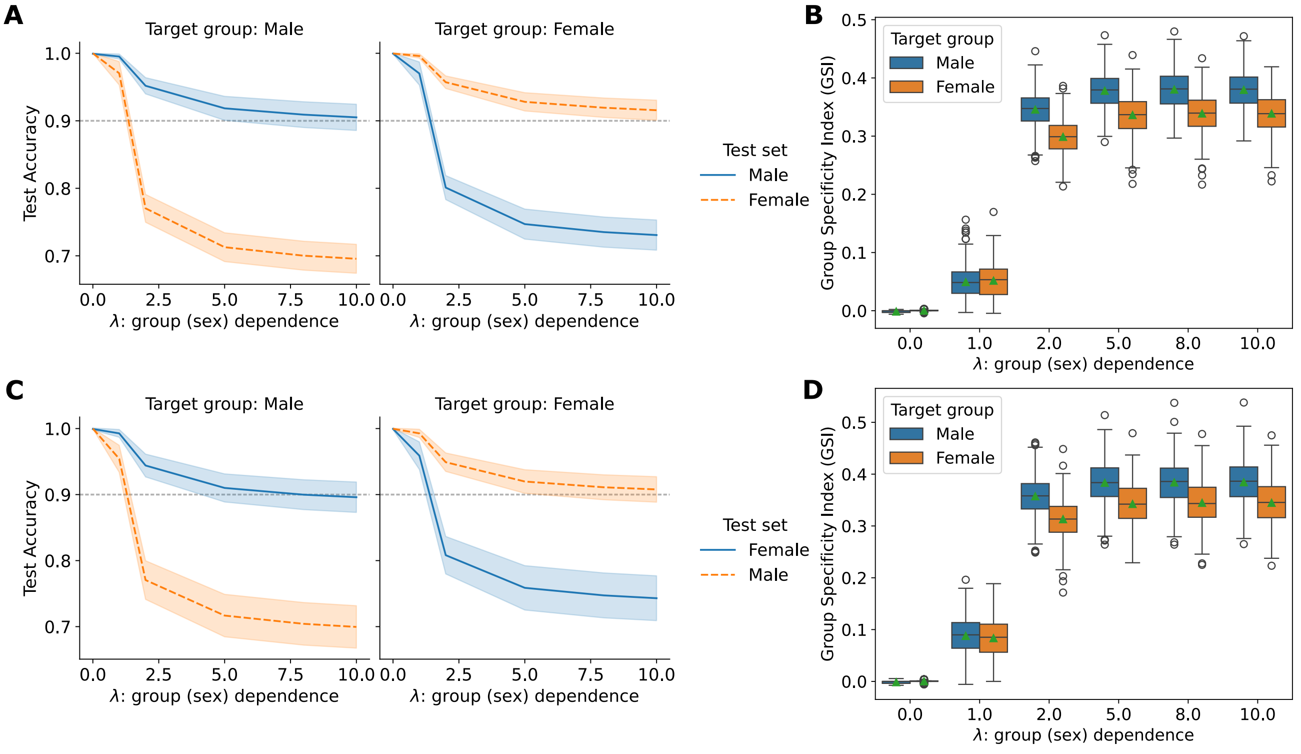

Supplementary Fig. S 2. Experimental results of left vs. right brain classification on male and female sets from the Brain Genomics Superstruct Project (GSP) [46] using GSDA-Logit with respect to the hyperparameter λ. **(A)** Average test accuracy on the held-out hemispheres, with a cross-validation strategy consistent with the one in Fig. 2A. **(B)** GSI calculated from the test results shown in Supplementary Fig. S2A. **(C)** Average test accuracy on the held-out subjects' data, with a cross-validation strategy consistent with the one in Supplementary Fig. S1C. **(D)** GSI calculated from the test results shown in Supplementary Fig. S2C. The remaining detailed descriptions of the figures, along with the main observations, are the same as those in the caption of Fig. 2.

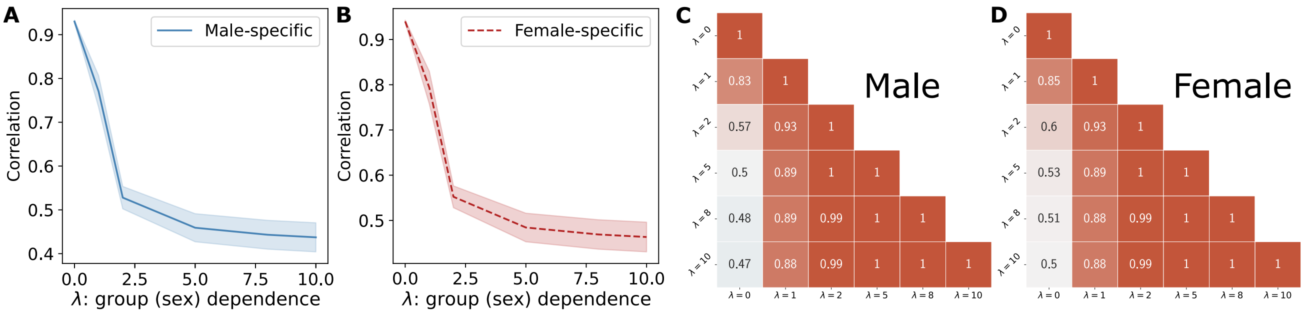

Supplementary Fig. S 3. Pearson correlation coefficients between model weights learned from GSP data [46]. (**A**) Correlation between male-specific and multivariate control models. (**B**) Correlation between female-specific and multivariate control models. (**C**) and (**D**) Average pairwise correlation for (**C**) male-specific and (**D**) female-specific GSDA models. The main observations are consistent with those in Fig. 3.

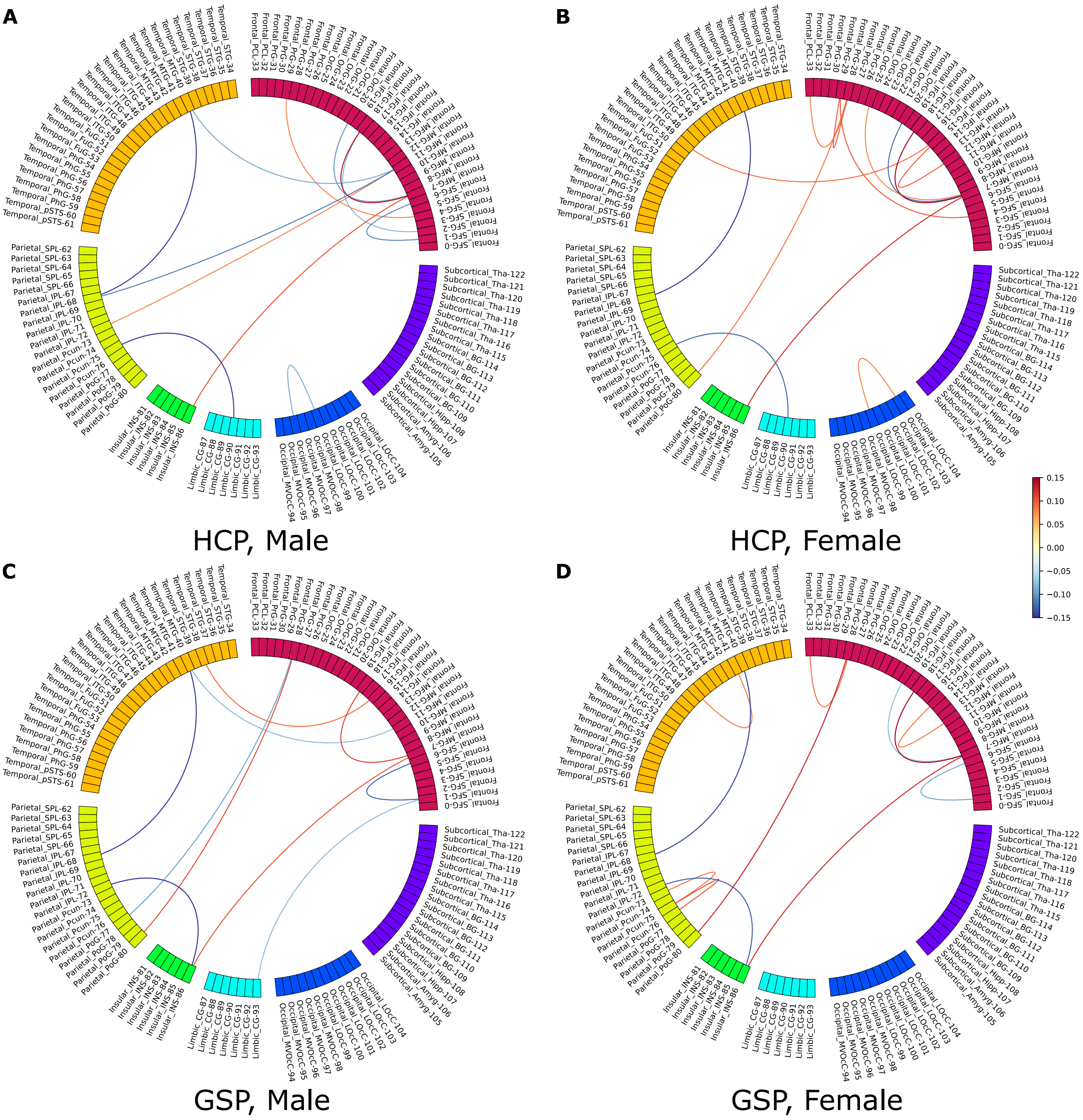

Supplementary Fig. S 4. Sex-specific lateralized connections identified by (**A**) male-specific models for HCP (Fig. 6A + Fig. 6E), (**B**) female-specific models for HCP (Fig. 6B + Fig. 6F), (**C**) male-specific models for GSP (Fig. 6C + Fig. 6G), and (**D**) female-specific models for GSP (Fig. 6D + Fig. 6G).

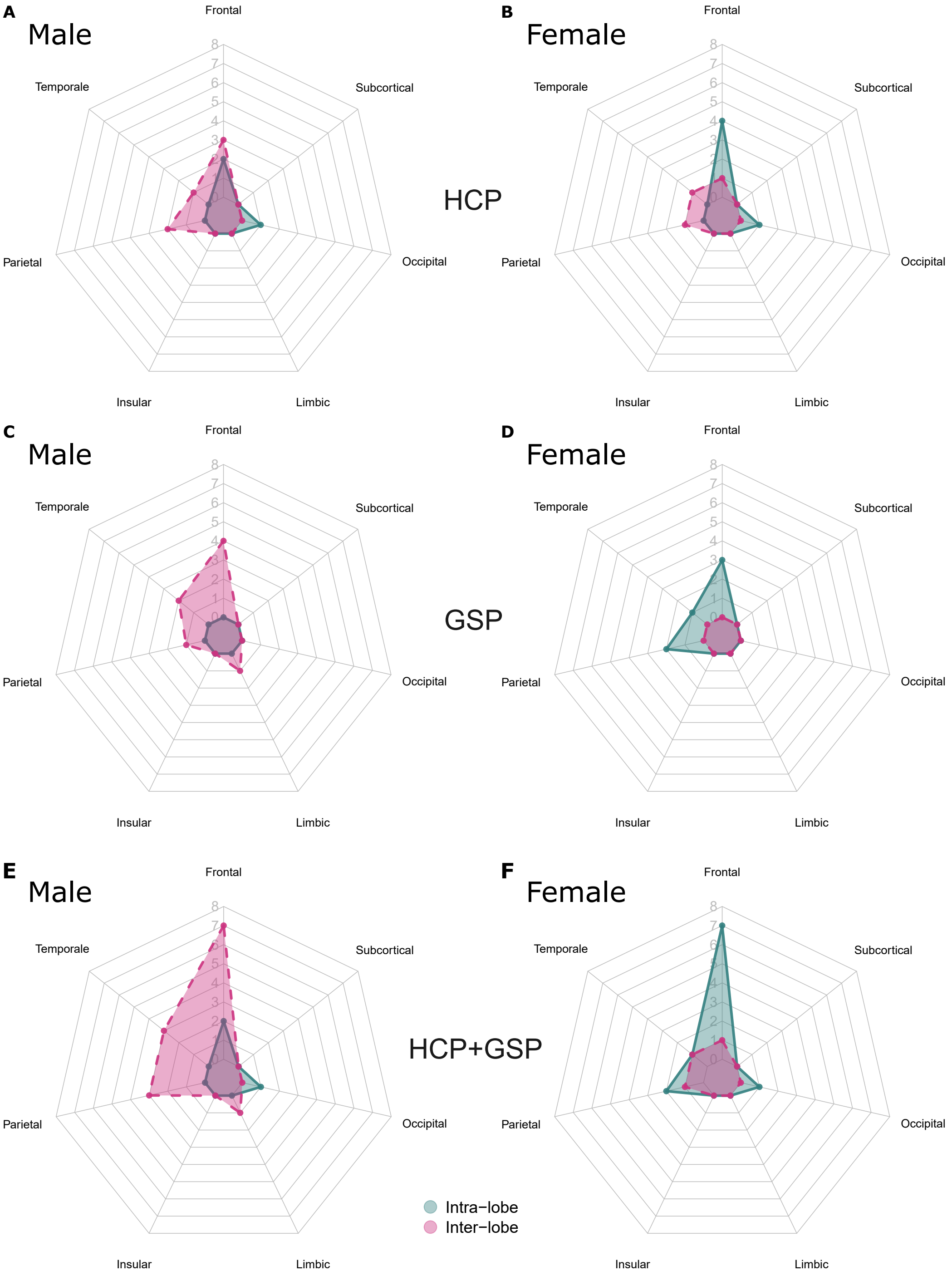

Supplementary Fig. S 5. Count of the group "exclusive" lateralized connections for HCP and GSP (Fig. 6, E to H) categorized by associated lobes, and inter- or intra-lobe. The connections are identified by **(A)** male-specific models for HCP, **(B)** female-specific models for HCP, **(C)** male-specific models for GSP, **(D)** female-specific models for GSP. **(E)** Sum of Supplementary Fig. S4, A and C. **(F)** Sum of Supplementary Fig. S4, B and D.
