# Supplementary material for: Group-specific discriminant analysis enhances detection of sex differences in brain functional network lateralization
Source: Gigascience. 2025 Aug 30;14:giaf082. doi: 10.1093/gigascience/giaf082 (PMC12398281; doi:10.1093/gigascience/giaf082)
Supplement: giaf082_GIGA-D-24-00330_Revision_1 [file giaf082_giga-d-24-00330_revision_1.pdf]

GigaScience

Group-specific discriminant analysis enhances detection of sex differences in brain functional network lateralization

--Manuscript Draft--

Manuscript Number:	GIGA-D-24-00330R1
Full Title:	Group-specific discriminant analysis enhances detection of sex differences in brain functional network lateralization
Article Type:	Research
Funding Information:	National Natural Science Foundation of China (T2325006) — Prof Gaolang Gong<br>National Natural Science Foundation of China (82021004) — Prof Gaolang Gong<br>Ministry of Science and Technology of the People's Republic of China (2021ZD0200500) — Prof Gaolang Gong<br>Ministry of Science and Technology of the People's Republic of China (2021ZD0201701) — Prof Gaolang Gong<br>Fundamental Research Funds for Central Universities of the Central South University (2233200020) — Prof Gaolang Gong
Abstract:	Background: Lateralization is the asymmetry in function and cognition between the brain hemispheres, with notable sex differences. Conventional neuroscience studies on lateralization use univariate statistical comparisons between male and female groups, with limited and ineffective validation for group specificity. This article proposes to model sex differences in brain functional network lateralization as a dual-classification problem: first-order classification of left vs. right hemispheres and second-order classification of male vs. female models. To capture sex-specific patterns, we develop an interpretable Group-Specific Discriminant Analysis (GSDA) for first-order classification, followed by logistic regression for second-order classification.<br>Findings: Evaluations on two large-scale neuroimaging datasets show GSDA's effectiveness in learning sex-specific patterns, significantly improving model group specificity over baseline methods. Major sex differences were identified in the strength of lateralization and interaction patterns within and between lobes.<br>Conclusions: The GSDA-based analysis challenges the conventional approach to investigating group-specific lateralization and indicates that previous findings on sex-specific lateralization will need revisits and revalidation. This method is generic and can be adapted for other group-specific analyses, such as treatment-specific or disease-specific studies.
Corresponding Author:	Shuo Zhou, Ph.D.<br>The University of Sheffield<br>Sheffield, South Yorkshire UNITED KINGDOM
Corresponding Author Secondary Information:	
Corresponding Author's Institution:	The University of Sheffield
Corresponding Author's Secondary Institution:	
First Author:	Shuo Zhou, Ph.D.
First Author Secondary Information:	
Order of Authors:	Shuo Zhou, Ph.D.<br>Junhao Luo<br>Yaya Jiang<br>Haolin Wang

Powered by Editorial Manager® and ProduXion Manager® from Aries Systems Corporation

	Haiping Lu
	Gaolang Gong
**Order of Authors Secondary Information:**	
**Response to Reviewers:**	Please see the supplementary material titled "GIGA-D-24-00330 response to reviewers" for our detailed and formatted responses.
**Additional Information:**	
**Question**	**Response**
Are you submitting this manuscript to a special series or article collection?	No
**Experimental design and statistics**<br><br>Full details of the experimental design and statistical methods used should be given in the Methods section, as detailed in our Minimum Standards Reporting Checklist. Information essential to interpreting the data presented should be made available in the figure legends.<br><br>Have you included all the information requested in your manuscript?	Yes
**Resources**<br><br>A description of all resources used, including antibodies, cell lines, animals and software tools, with enough information to allow them to be uniquely identified, should be included in the Methods section. Authors are strongly encouraged to cite Research Resource Identifiers (RRIDs) for antibodies, model organisms and tools, where possible.<br><br>Have you included the information requested as detailed in our Minimum Standards Reporting Checklist?	Yes
**Availability of data and materials**<br><br>All datasets and code on which the conclusions of the paper rely must be either included in your submission or deposited in publicly available repositories	Yes

(where available and ethically appropriate), referencing such data using a unique identifier in the references and in the “Availability of Data and Materials” section of your manuscript.<br><br>Have you have met the above requirement as detailed in our Minimum Standards Reporting Checklist?	

Manuscript

Click here to access/download;Manuscript;GIGA-D-24-00330.pdf

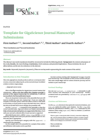

GigaScience, 2025, 1–18

doi: xx.xxxx/xxxx
Manuscript in Preparation
Research

RESEARCH

Group-specific discriminant analysis enhances detection of sex differences in brain functional network lateralization

Shuo Zhou [0000-0002-8069-2814][1,2,3,†], Junhao Luo [0000-0002-3207-1597][4,7,8,†], Yaya Jiang [0000-0001-7011-4175][4,9], Haolin Wang [0000-0002-1352-8387][1,2], Haiping Lu [0000-0002-0349-2181][1,2,3,*] and Gaolang Gong [0000-0001-5788-022X][4,5,6,*]

[1]School of Computer Science, University of Sheffield, Sheffield, UK and [2]Centre for Machine Intelligence, University of Sheffield, Sheffield, UK and [3]Neuroscience Institute, University of Sheffield, Sheffield, UK and [4]State Key Laboratory of Cognitive Neuroscience and Learning & IDG/McGovern Institute for Brain Research, Beijing Normal University, Beijing, China and [5]Beijing Key Laboratory of Brain Imaging and Connectomics, Beijing Normal University, Beijing, China and [6]Chinese Institute for Brain Research, Beijing, China and [7]Shenzhen CyberAray Network Technology Co., Ltd, Shenzhen, China and [8]Harbin Institute of Technology, Shenzhen, Shenzhen, China and [9]Artificial Intelligence and Language Cognition Laboratory, Beijing International Studies University, Beijing, China

[*]h.lu@sheffield.ac.uk; gaolang.gong@bnu.edu.cn
[†]Contributed equally.

Abstract

Background: Lateralization is the asymmetry in function and cognition between the brain hemispheres, with notable sex differences. Conventional neuroscience studies on lateralization use univariate statistical comparisons between male and female groups, with limited and ineffective validation for group specificity. This article proposes to model sex differences in brain functional network lateralization as a dual-classification problem: first-order classification of left vs. right hemispheres and second-order classification of male vs. female models. To capture sex-specific patterns, we develop an interpretable Group-Specific Discriminant Analysis (GSDA) for first-order classification, followed by logistic regression for second-order classification.
Findings: Evaluations on two large-scale neuroimaging datasets show GSDA's effectiveness in learning sex-specific patterns, significantly improving model group specificity over baseline methods. Major sex differences were identified in the strength of lateralization and interaction patterns within and between lobes.
Conclusions: The GSDA-based analysis challenges the conventional approach to investigating group-specific lateralization and indicates that previous findings on sex-specific lateralization will need revisits and revalidation. This method is generic and can be adapted for other group-specific analyses, such as treatment-specific or disease-specific studies.

Key words: Group-specific analysis; Sex-specific lateralization; Brain functional network; Dual-classification; Group-specific discriminant analysis

Background

Human brains are functionally asymmetric [1, 2]. These differences between left and right brain hemispheres are believed to reflect a complex interplay of evolutionary, hereditary, developmental, experiential, and pathological influences [3, 4, 5, 6]. One important understanding is that multiple factors influence human brain lateralization [7], with sex being one of the most representative [8, 9, 10, 11]. A popular hypothesis is that males typically have a more asymmetric brain organization, with the left hemisphere specialized for verbal processing and the right for spatial processing. In contrast, females tend to have a more "bilateral" brain organization, where both hemispheres are involved in verbal processing. The origin of such sex differences in functional lateralization has been attributed to neurobiological mechanisms involving genetic and hormonal factors [7, 12]. Genetically, X-inactivation in females leads to cortical mosaicism and functional flexibility [13], and Y-linked genes in males promote asymmetric development [14]. Hormonally, Testosterone is associated with promoting right-hemisphere dominance through delayed maturation of the left hemisphere [15], while estrogen enhances interhemispheric connectivity

Compiled on: June 17, 2025.
Draft manuscript prepared by the author.

Key Points

- Conventional multivariate and univariate methods identified common but not specific lateralization patterns through within-group analysis.
- Our Group-Specific Discriminant Analysis (GSDA) based method identified sex-specific lateralization patterns, validated through cross-validation and shown to be distinct from those identified by conventional methods.
- Nearly half of the specifically lateralized functional connections are shared by both males and females, with sex differences observed in the strength of lateralization.
- Stronger positive inter-lobe interactions are more left-lateralized in male brain networks, whereas stronger positive intra-lobe interactions are more right-lateralized in female brain networks.

[16, 17, 18, 19, 20]. Moreover, recent brain magnetic resonance imaging (MRI) studies [21, 22, 23] have shown that males tend to have smaller corpus callosums and larger amygdalae, which may limit interhemispheric communication. In contrast, females typically have larger corpus callosums that enhance integration between hemispheres. These findings potentially explain the more lateralized brain organization in males and the more bilateral organization in females.

Neurobiological sex differences and brain lateralization have also been observed in several psychiatric disorders, as reported in multiple brain MRI studies [24, 25, 26, 27]. For example, depression, anxiety, schizophrenia, and autism spectrum disorder exhibit notable sex differences in incidence rates and clinical manifestations [24, 25]. Additionally, several studies on brain MRIs have demonstrated that brain lateralization abnormalities are linked with conditions such as major depressive disorder (MDD) [26] and schizophrenia [27]. Understanding these sex-specific and lateralized brain alterations may contribute to personalized diagnosis, prognosis, treatment, and further uncovering the pathogenesis of these diseases.

Measuring brain functional lateralization is valuable but challenging [28]. Direct approaches such as selectively modulating or suppressing cortical activities and circuits in a single hemisphere [29] often pose a risk of inflicting harm on the human brains [30]. Over the last two decades, functional neuroimaging techniques have been widely used in neuroscience, offering a powerful and non-invasive approach for investigating brain lateralization [31, 32]. One popular technique is analyzing functional connectivity (FC), also known as brain networks or connectomes [33]. This is usually derived from resting-state functional MRI (rs-fMRI) time series and considered an intrinsic "fingerprint" of each individual's brain [34, 35, 36]. A previous study [10] reported sex differences in the lateralization of resting-state networks, with more right-lateralized visual and default-mode network components for males and females, respectively. Additionally, males and females have also demonstrated significant differences in homotopic functional connectivity of various regions [37].

Studies on brain lateralization have largely focused on modeling asymmetry effects region-by-region via univariate analysis [7]. These lateralized brain regions are usually measured using the laterality index (LI) [4, 6, 38, 39], or identified through statistical tests comparing homologous regions [40]. Previous analyses identifying sex-specific brain lateralization have typically adopted within-group univariate methods [22, 41, 42, 43]. For example, to understand male-specific lateralization, analyses are performed separately on male and female data, labeling features that significantly differ from female data as "male-specific". There are two main limitations for this approach. First, univariate approaches generally lack robust, data-driven validation, meaning models derived from these analyses cannot be tested on unseen data samples. Specifically, for identifying sex-specific lateralization patterns, univariate frameworks cannot provide effective mechanism for validating the generalizability and specificity of models obtained separately from male and female samples, raising questions about the reliability of such results and findings. Second, univariate analyses may not be able to capture complex interactions among multiple neuroimaging features, potentially overlooking critical multivariate patterns underlying sex differences in lateralization. Additionally, the substantial anatomical and functional similarities between male and female brains, combined with typically small statistical effect sizes, further complicate the reliable detection of genuine sex differences in lateralization [44]. Consequently, subtle sex-specific lateralization patterns may be overshadowed by broad similarities.

Here, we address the challenges of detecting and validating sex differences in brain lateralization by framing the problem as a machine learning classification task, making the following key methodological contributions: *First*, we propose a dual-classification workflow to identify, validate, and interpret multivariate patterns of sex-specific lateralization. This consists of a first-order classification of left vs. right brain hemispheres and a second-order classification of male- vs. female-specific models. The resulting model weights represent lateralization strength and sex difference significance, respectively. Figure 1A presents the whole workflow. *Second*, we propose a novel group-specific discriminant analysis (GSDA) algorithm (Fig. 1B) to learn group (sex)-specific models in the first-order classification. *Third*, we leverage cross-validation to statistically evaluate the learned lateralization patterns by assessing model accuracy on male and female test samples. Furthermore, we propose a Group Specificity Index (GSI) to measure the group specificity of the learned models.

Our final contribution provides interpretation for the experimental results using the intrahemispheric connections extracted from rs-fMRI data of two large-scale public neuroimaging repositories, the Human Connectome Project (HCP) [45] and the Brain Genomics Superstruct Project (GSP) [46]. A significant GSI improvement over the baselines demonstrates the effectiveness of GSDA in learning group-specific models. Further interpretation of the dual-classification model weights reveals consistent sex differences in lateralization across datasets: 1) *about half of the sex-specific lateralized connections are shared between male and female brain functional network, with differences in the strength of lateralization*, 2) *stronger positive inter-lobe interactions are more left-lateralized in the male brain networks, while stronger positive intra-lobe interactions are more right-lateralized in the female brain networks*.

Results

Diverged test accuracy on male and female sets

Figure 2 depicts the performance of group-specific discriminant analysis (GSDA) in classifying left vs. right brain hemispheres on the HCP data [45], across a varied range of values for hyperparameter λ. A larger λ indicates a higher grouping factor (sex) dependence. When the target group is male (the left of Fig. 2A), the labels for the left and right hemispheres of the female training data were masked. Therefore, the training female samples were only involved in the grouping factor dependence regularization. In this scenario, the average accuracy obtained on the male test samples (the blue solid line) stays higher than that on the female test samples (the orange dashed line). The increase of λ leads to an increased gap between the test accuracy on target and non-target test sets. In particular, this discrepancy widens significantly within the range $0 < \lambda \leq 5$ and stabilizes to a 20% gap for $\lambda > 5$ (Fig. 2A). These observations remain consistent in results with two different cross-validation strategies for the HCP data (Supplementary Fig. S1, A and C) and the GSP data (Supplementary Fig. S2, A and C). A theoretical interpretation is provided in the **Methods** section to validate this divergence.

The group specificity of models obtained by GSDA increases with a larger λ, as reflected by our proposed metric, the Group Specificity Index (GSI), which is presented as a box plot in Fig. 2B. When $0 < \lambda \leq 5$, the GSI for both male- and female-specific GSDA models increases with the increase of λ. When $\lambda \geq 5$, the GSI maintains at around 0.4. Based on both accuracy and GSI results, $\lambda = 5$ is an "elbow" point in the experiment across different datasets and cross-validation strategies, which can be considered an optimal value for the trade-off between classification accuracy, group specificity, and model

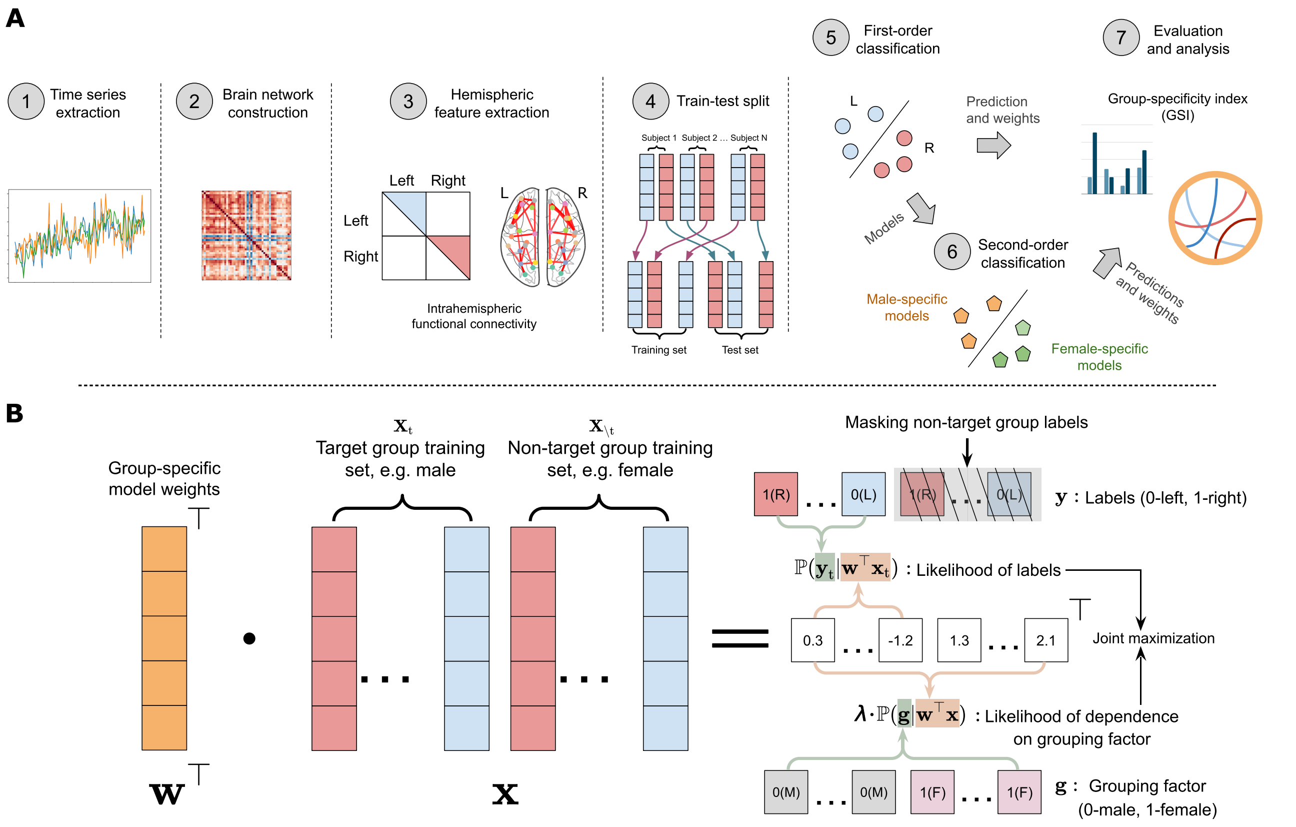

Figure 1. The proposed workflow for detecting sex-specific brain lateralization via Group-Specific Discriminant Analysis (GSDA). **(A)** Overview of the proposed classification workflow. ①–③: Hemispheric features are extracted from the intrahemispheric brain functional network, constructed from resting-state functional MRI time series. ④⑤: First-order classification learns the differences between the two hemispheres, where we propose a group-specific discriminant analysis (GSDA) classifier to classify left vs. right hemispheres for a target group. ⑥: Second-order classification trains a standard logistic regression for classifying the male- vs. female-specific models obtained from the first-order classification, to identify the weights that significantly contribute to the sex-specific predictions. ⑦: Prediction results evaluation and model weight interpretation. **(B)** GSDA with the logistic loss (GSDA-Logit) for the first-order classification. This model jointly maximizes the likelihood of labels for the target group (with non-target-group labels masked out) and the grouping factor dependence for both the target and non-target groups, where $^\top$ denotes the transpose of vectors, $\mathbf{x}$ denotes the input training samples, $\mathbf{x}_t$ denotes target group training samples, $\mathbf{x}_{\backslash t}$ denotes non-target group samples, and a hyperparameter $\lambda \geq 0$ controls the grouping factor dependence. A larger λ corresponds to a higher dependence. When $\lambda = 0$, GSDA-Logit degenerates to a standard logistic regression for the target-group data.

complexity (the hyperparameter for the ℓ_2 regularization in GSDA was fixed to 0.1, so the larger λ, the lower relative importance of the ℓ_2 regularization). Hence, in the rest of this article, we will use $\lambda = 5$ for GSDA as the main sex-specific model to present the results and findings.

In contrast, the GSI steadily approaches zero without the grouping factor dependence regularization. At $\lambda = 0$, where GSDA degenerates to a standard logistic regression trained only on the target-group hemispheres, the accuracy is nearly 100% for both male and female test samples (Fig. 2A, Table 1, Supplementary Fig. S1, A and C, and Supplementary Fig. S2, A and C). This performance is similar to the multivariate control baseline, which uses standard logistic regression trained on mixed male and female hemispheres. From Table 1, the control models achieved an accuracy of 99.99 $\pm$ 0.04% for male and 99.92 $\pm$ 0.13% for female HCP test samples, and 99.94 $\pm$ 0.07% for male and 99.99 $\pm$ 0.01% for female GSP test samples. Additionally, because of the same property and similar performance compared to the standard logistic regression (multivariate control baselines), we will view GSDA with $\lambda = 0$ as an additional multivariate baseline.

GSDA-based models learned distinct weights

Beyond classification performance similarity, the weights of multivariate baselines (control and GSDA with $\lambda = 0$) are also highly correlated. As shown in Fig. 3A, the average Pearson correlation coefficients between multivariate baselines are 0.99 for analyses conducted within either HCP or GSP data. Similarly, in univariate analyses based on the t-test of paired left and right connections, the t-values of within-group analysis showed a 0.99 correlation with the t-values derived from mixed male and female samples (univariate control). Among these multivariate and univariate baselines, the correlation for any arbitrary pair exceeds 0.91 for within-dataset results and 0.7 for cross-dataset results. These correlations represent large (≥ 0.8) and medium ($0.5 - 0.8$) effects, respectively, according to the thresholds for interpreting the effect size of Pearson's correlation [47, 48]. *This high correlation suggests that the lateralization modeled by multivariate or univariate baselines is common to both males and females, regardless of whether the analysis is conducted with exclusively male or female data, or with mixed data.* This corresponds to the top red triangular cluster in Fig. 3A.

In contrast, our sex-specific models (with a higher GSI) show lower correlations with the univariate and multivariate baseline models. This corresponds to the blue rectangular cluster at the bottom of Fig. 3A, where a majority of coefficients fall within the range of 0.35 to 0.5 (below the thresholds of medium effects [47, 48]). Increasing the value of λ leads to a decreasing correlation between the control and GSDA models ($\lambda > 0$), for both results from HCP (Fig. 3B, and first columns of Fig. 3, C and D) and GSP (Supplementary Fig. S3, A and B, and first columns of Supplementary Fig. S3, C and D). Moreover, the weights of sex-specific models are stable. As shown in Fig. 3, C and D, the average correlation of any pair for GSDA with $\lambda \geq 2$ is 0.99 or above.

Frontal lobe shows most sex-specific lateralized connections

To identify sex-specific lateralized connections among the 7,503 intrahemispheric connections, we performed a second-order classification. This involved training standard logistic regression models to distinguish between male- and female-specific models learned from the first-order classification, using 80% of the first-order models for training and 20% for testing. The test accuracy for second-order classification consistently achieved nearly 100% over 1,000 random splits. This indicates that the sex differences in the first-order GSDA

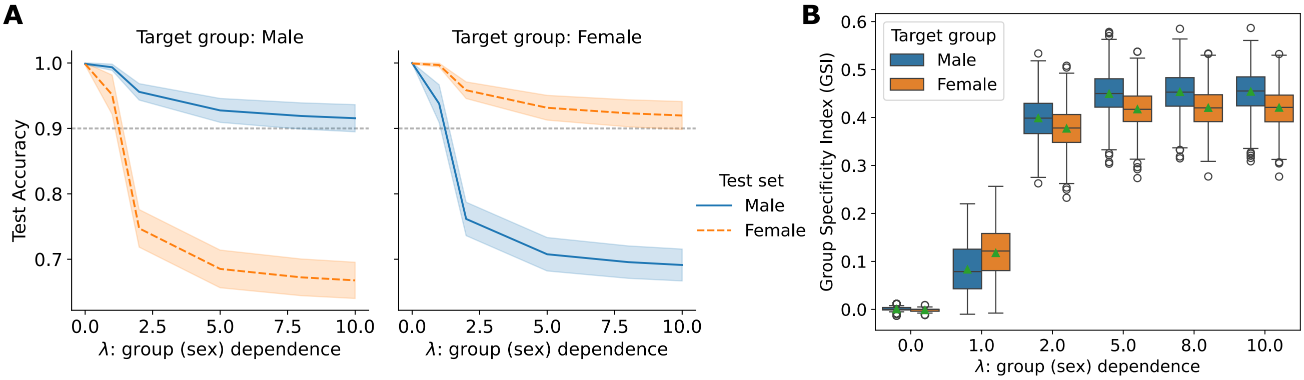

Figure 2. Diverged test accuracy and improved Group Specificity Index (GSI) of left vs. right brain classification on male and female sets from the Human Connectome Project (HCP) [45] using the proposed Group-Specific Discriminant Analysis with the logistic loss (GSDA-Logit) with respect to the hyperparameter λ, which controls the grouping factor (sex) dependence. There were 1,000 random training-testing partitions for the experiment, where each subject randomly contributed one hemisphere for training and the other for testing, resulting in 50% brain hemispheres being selected as training samples. **(A)** The test accuracy on male and female sets increasingly diverges with the increase of dependence on sex (λ). The average test accuracy is represented by solid or dashed lines, with standard deviations shown as error bands, computed across 1,000 random training-testing partitions. **(B)** The GSI calculated from the results in Fig. 2A increases with λ. The horizontal lines in each box represent the 25th percentile, median, and 75th percentile of the GSI over the 1,000 test sets, respectively, from bottom to top, and the green triangles represent the mean. The average GSI approaches 0 when $\lambda = 0$, indicating that models learned without group dependence regularization captured common instead of specific patterns, despite being trained on only male (or female) data.

Table 1. First-order classification (left vs. right brain hemispheres) accuracy on male and female test sets from the HCP [45] & Brain Genomics Superstruct Project (GSP) [46]. Group-specific models (GSDA with $\lambda = 5$) are compared with three multivariate baselines: (1) standard logistic regression trained on a mixture of male and female training data, (2) GSDA with $\lambda = 0$ (equivalent to standard logistic regression) trained on male data only, and (3) GSDA with $\lambda = 0$ trained on female data only. $\lambda = 5$ is an optimal value for GSDA on the data as determined by the accuracy and GSI in Fig. 2. The baselines achieved similar accuracy on male and female test sets, indicating a lack of group specificity. Conversely, the group-specific models maintained accuracy on the target test set but showed a significant gap with the lower accuracy on the non-target test set.

Classification method (target group)	Average test accuracy (%) and gap (|accuracy difference|)					
	HCP male	HCP female	HCP gap	GSP male	GSP female	GSP gap
Logistic regression (male + female)	99.99 ± 0.04	99.96 ± 0.10	0.03	99.93 ± 0.13	99.96 ± 0.10	0.05
GSDA ($\lambda = 0$, male)	99.87 ± 0.16	99.85 ± 0.17	0.02	99.93 ± 0.08	99.99 ± 0.01	0.06
GSDA ($\lambda = 0$, female)	99.93 ± 0.12	99.99 ± 0.04	0.06	99.97 ± 0.05	99.95 ± 0.07	0.02
GSDA ($\lambda = 5$, male)	92.75 ± 1.83	68.52 ± 2.88	24.23	91.85 ± 1.77	71.28 ± 2.13	20.57
GSDA ($\lambda = 5$, female)	70.76 ± 2.56	93.16 ± 1.89	22.40	74.70 ± 2.22	92.81 ± 1.35	18.11

model weights are generalizable from the training set to the test set.

Based on the weights from these second-order classification models, we derived a mask that characterizes sex differences in the lateralized connections. We first averaged the weights across 1,000 second-order models from different random splits for the HCP and GSP datasets, respectively. Then, we identified the overlap between the top 5% of the largest average weights (by magnitude) from HCP and those from GSP. The resulting map is represented by the chords in Fig. 4A. The threshold of 5% was chosen because the second-order logistic regression classifiers were trained with ℓ_2 regularization, which can be interpreted as a Gaussian prior (normal distribution) on model weights, with 5% being a commonly used statistical significance level for a Gaussian distribution. By calculating the average degree [50] (Fig. 4B) of connections for each lobe within this mask, we can learn that sex differences in first-order weights are associated with the frontal, parietal, and occipital lobes, where the average degrees exceed 1. The frontal lobe shows the largest average degree, indicating significant sex differences.

We then applied this mask to the top 5% weights of four first-order classification models: HCP male-specific, HCP female-specific, GSP male-specific, and GSP female-specific. The obtained lateralized connections with sex differences are shown in Fig. 5, Fig. 6, A to H, and Supplementary Fig. S4, A to D. The weights of these four models were obtained by taking the average of the corresponding 1,000 models learned from first-order classification with different random splits. In total, 47 lateralized connections with repetition were identified, of which 30 connections are unique. Among these 47 connections, the middle frontal gyrus (MFG) was the most frequently involved region, suggesting it may serve as a hub. Specifically, 17 out of the 47 connections were associated with the MFG in both male and female samples across both datasets.

Sex-specific lateralization: shared and "exclusive" connections

For each of the four sex-specific models, half of the identified lateralized connections are shared between male and female brain networks (Fig. 5) on average: on HCP, 6 out of 12 for the male-specific model and 6 out of 13 for the female-specific model; on GSP, 6 out of 10 for the male-specific model and 6 out of 12 for the female-specific model. Among the 30 unique sex-specific lateralized connections identified across datasets, 9 (nearly one-third) are shared between males and females. To illustrate these findings, we have separated the shared connections (Fig. 6, A to D) and "exclusive" connections (Fig. 6, E to H).

For the shared lateralized connections (Fig. 6, A and B for HCP and Fig. 6, C and D for GSP), we observed sex differences in the magnitude of first-order weights, i.e., the strength of lateralization. Specifically, for the female-specific models, the magnitudes of the first-order weights corresponding to the connections associated with the frontal lobe are generally larger compared to those for male-specific models, particularly those of the positive weights. In the male-specific models, the magnitudes of the first-order weights for connections related to other lobes are larger than those in the female-specific models.

For the "exclusive" connections (Fig. 6, E to H), the male-specific models contain more inter-lobe lateralized connections (Fig. 7A), with over 70% of corresponding weights being negative, shown by the blue chords in Fig. 6, E and G. Female-specific models, on the other hand, contain more intra-lobe lateralized connections (Fig. 7B), with over 90% of weights being positive, indicated by the red chords in Fig. 6, F and H. Notably, these patterns of inter- and intra-lobe lateralization for males and females are consistent across joint or separate analyses of both HCP and GSP data (Supplementary Fig. S5), demonstrating the stability and reliability of these findings.

Discussion

Cross-validation-based learning challenges conventional statistical approach for investigating specific lateralization

Traditional neuroscience studies commonly assume that results from within-group analyses are specific to the group being studied [42, 41, 43]. While

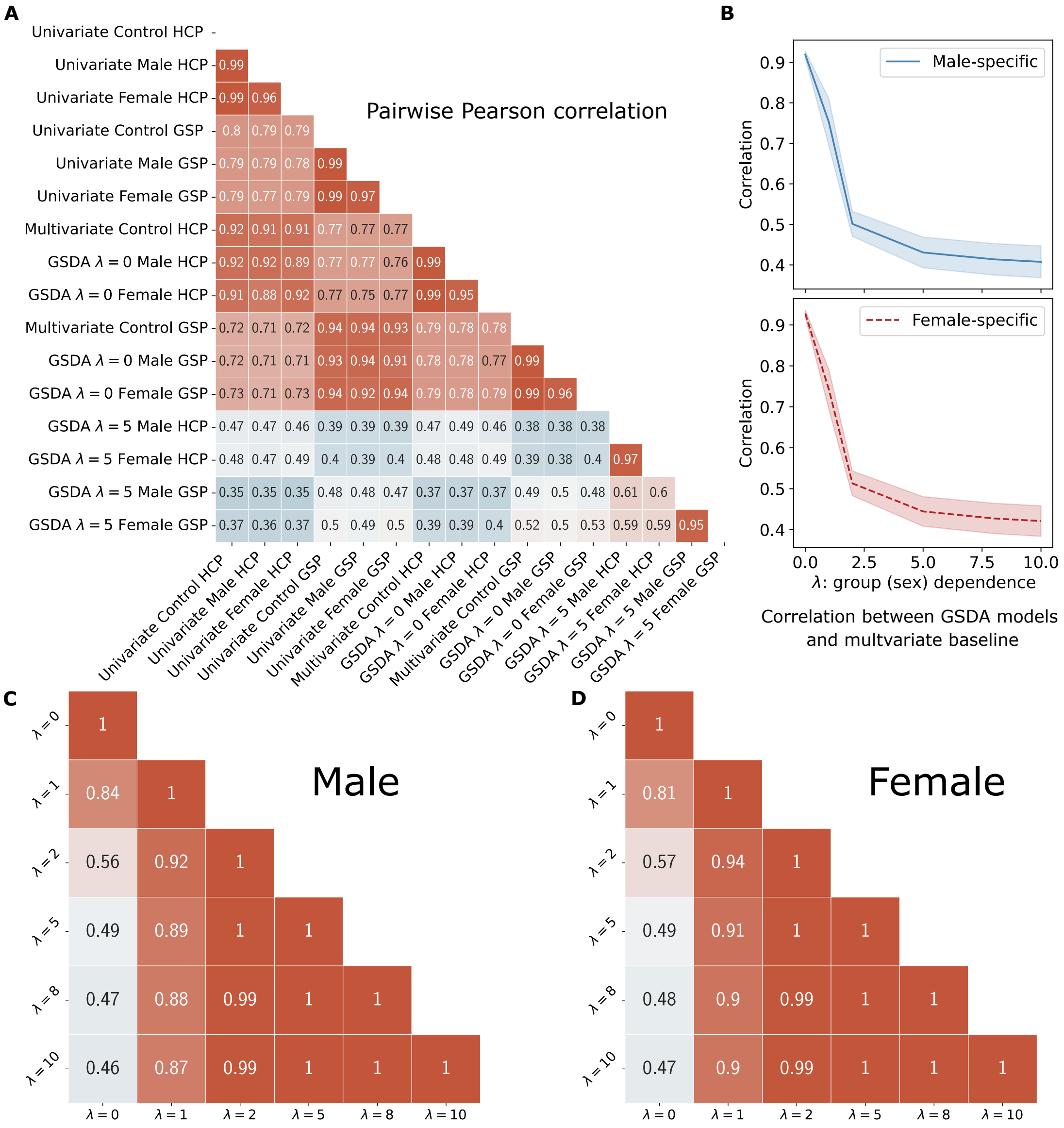

Figure 3. Pearson correlation between model weights. **(A)** Pairwise correlation between weights of 16 models, including multivariate models from Table 1 and univariate models, labeled along the x- and y-axis. Two clusters can be observed here: multivariate and univariate baselines versus GSDA with $\lambda = 5$. **(B)** Correlation between GSDA and multivariate control models (trained on mixed male and female data) on the HCP data. As λ increases, the GSDA models become less correlated with the control models. **(C), (D)** Average pairwise correlation for **(C)** male-specific and **(D)** female-specific GSDA models trained on HCP data, with respect to λ. The weights of sex-specific models remain stable (correlation ≥ 0.99) for $\lambda \geq 2$.

using only male or female data to explore sex-specific characteristics may seem intuitive, our cross-validation results challenge this assumption. For example, as shown in Fig. 2A, standard logistic regression models trained exclusively on data from a single target group (male or female) achieved nearly identical performance on test sets from both the target and non-target groups. Thus, these baseline models' performance appears insensitive to sex-based sampling. According to statistical learning theory [51], the similarity in generalization errors indicates that these models capture general patterns applicable to both males and females, rather than being sex-specific. This finding implies that statistical modeling can learn common patterns even using data from a specific group, contradicting the conventional assumption in group-specific analysis. This conclusion holds at least in our study on left vs. right brain classification using the HCP and GSP datasets.

In contrast, the performance of our sex-specific models (GSDA with $\lambda > 0$) is sensitive to sex-based sampling. The classification results (Fig. 2A, Supplementary Fig. S1, A and C, and Supplementary Fig. S2, A and C) reveal that the generalization error for the target-group test sets is significantly lower than that for the non-target-group test sets. This indicates a stronger specificity to sex compared to the multivariate baseline models, as reflected by our group-specificity index (GSI) results. The differences observed in test performance highlight the importance of cross-validation in validating the group specificity of statistical analysis results.

While univariate analysis results are not directly applicable to unseen samples for testing, the strong correlation between univariate and multivariate baselines offers valuable insights. For example, the correlation of the within-group univariate t-test results with univariate control models (mixed) and multivariate baselines exceeds 0.99 and 0.91, respectively. This suggests that the lateralization patterns from the t-values of our within-group univariate analyses are likely common to both males and females. Consequently, previous conclusions from such within-group analyses should be revisited and revalidated. Moreover, although multivariate methods are theoretically better at capturing interactions between features than univariate methods, the observed similarity in results suggests that multivariate approaches may not always reveal patterns beyond those identified by univariate analyses.

Sex-specific lateralized regions and connections across datasets

The mask resulting from the second-order classification revealed sex differences in connections across lobes, including the frontal, temporal, parietal, insular,

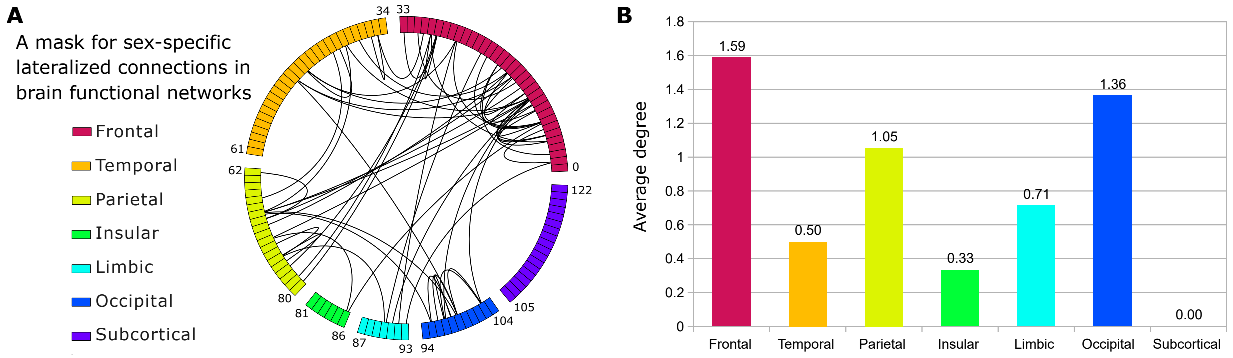

Figure 4. Sex-specific lateralized functional connection mask derived from GSDA-based dual-classification. **(A)** A chord-based mask for identifying sex-specific lateralized connections was derived in two steps: 1) averaging weights across 1,000 second-order models from different random splits for HCP and GSP data, respectively; 2) identifying overlaps between the top 5% largest average weights from HCP and those from GSP. The circle represents a brain hemisphere, and each cell on the rim represents a region of interest (ROI) within the half brain. The seven colors indicate seven functional parcellations defined in Brainnetome atlas (BNA) [49]. The numbers on the rim are the start and end ROI IDs of the lobe in the BNA atlas, where the 123 ROIs are labeled from 0 to 122. **(B)** The average lateralization degree [50] for each of the seven BNA lobes. It is calculated as the average number of chords per ROI, based on the chords and ROIs in Fig. 4A. The frontal lobe shows the largest degree of lateralization.

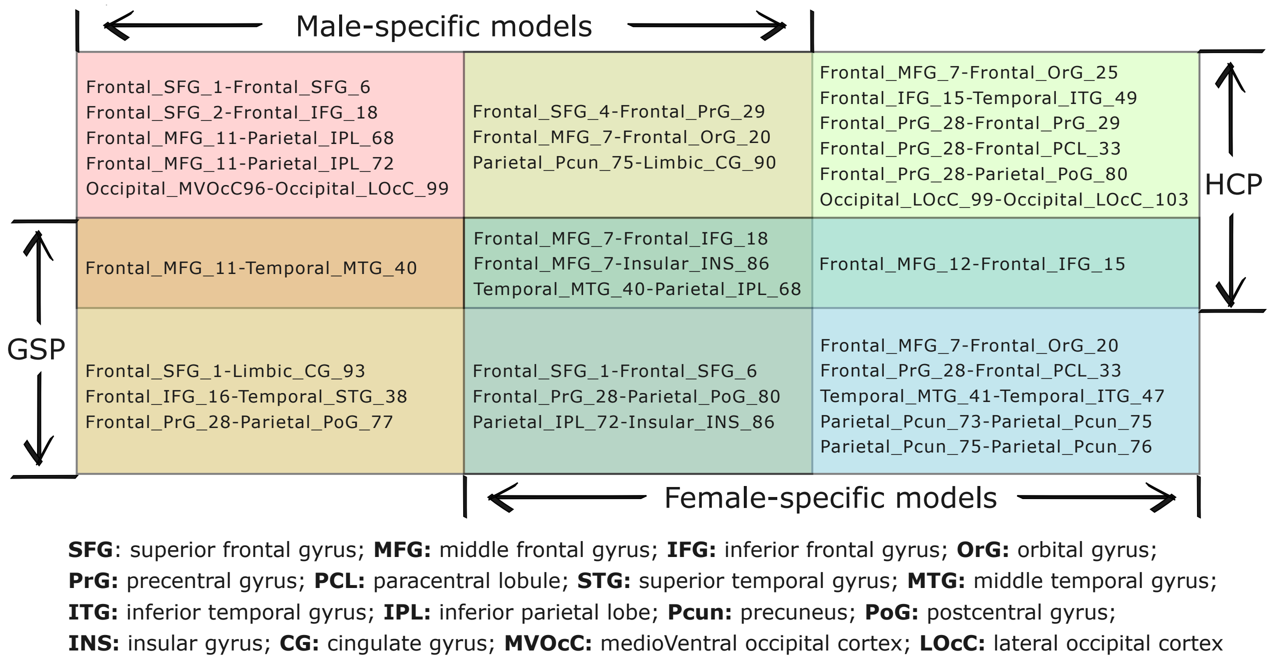

Figure 5. 30 unique sex-specific lateralized connections learned from HCP and GSP. Each connection is represented as "ROI-ROI", and each ROI is represented as "Lobe_Gyrus_ROI ID", where the lobe and gyrus are defined in BNA. 22 out of the 30 connections are associated with the frontal lobe. Nine connections are shared between males and females (middle column) with the differences between male and female models being in the strength of lateralization (Fig. 6, A to D). 21 connections are "exclusive" to one group (left and right column) with the differences between male and female models being in the patterns of inter-/intra-lobe interactions (Fig. 6, E to F, and Fig. 7).

limbic, and occipital lobes, where the functional differences between males and females were observed in previous studies [10, 52, 11, 53, 54]. Among the sex-specific lateralized connections, MTG-IPL, MFG-IFG, and MFG-INS are shared in both male- and female-specific models across the two datasets.

From the perspective of gyrus, which engages in various cognitive functions, the lateralized regions include the MTG[55] (sound recognition and language processing), MFG [56] (literacy and numeracy), IPL [57, 58] (spatial attention, multimodal sensory integration, and oculomotor control), IFG[59] (speech and language processing), and INS [60] (various sensorimotor processing and risk-reward behavior). These regions show lateralization and sex differences in certain functions including speech processing, language, and spatial attention [12, 10, 52]. The MFG, a hub region in this study, is a core component of the multiple demand system [61], and presents hemispheric specialization, with the left MFG primarily supporting literacy development, while the right MFG is vital for numeracy [56]. The MTG showed lateralization in activated volumes for both males and females during language tasks, while the lateralization of IFG was only observed in males [62]. Our study reports different weights of connections related to these two regions. This suggests that the lateralization of a region's external connections can reflect the lateralization of its functional activation. The "activity flow" theory in neuroscience has linked the connections and functional activation [63], proposing that the seed-based connection-weighted sums of the activation of other regions can predict functional activation of the seed region. Our results suggest *a correlation between the lateralization of functional connectivity and activation*, although further quantitative analysis is required to investigate the specifics of this relationship.

From the perspective of connections, our experimental results identified three sex-specific lateralized connections shared by males and females across datasets: MTG-IPL, MFG-IFG, and MFG-INS, aligning with established functional and structural mechanisms underlying sex differences in cognitive processing. The MTG-IPL connection is associated with language and picture-naming tasks, highlighting its role in integrating sensory and semantic information [64]. Studies have demonstrated notable plasticity in this connection, alongside sex differences in picture naming speed [65] and reliably left-lateralized evoked activations during picture-naming tasks [66]. Our GSDA framework captured these sex and hemispheric differences, suggesting that lateralization in picture naming might be driven by related connectivity, such as MTG-IPL. Variations in lateralization may be attributed to differences in the adaptive neurobiological functions of regions such as the orbitofrontal cortex (OFC), known to modulate

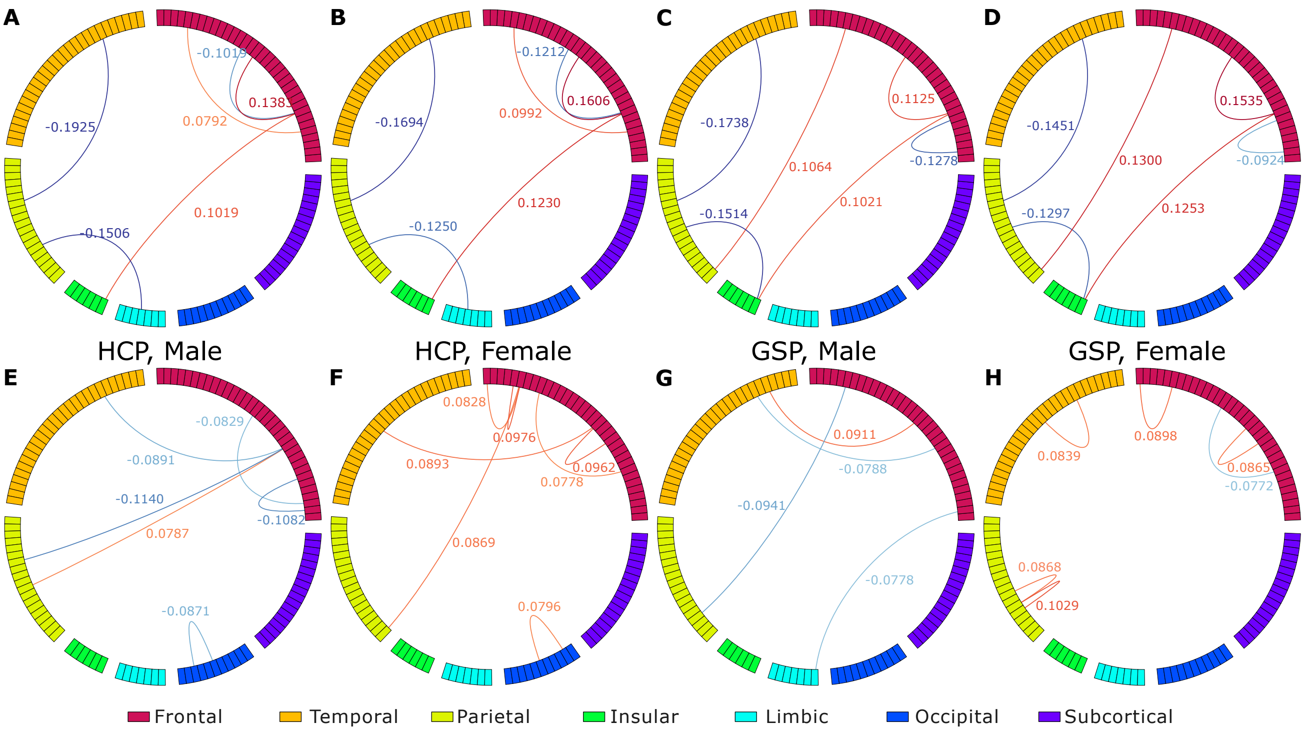

Figure 6. Sex-specific lateralized connections with first-order GSDA model (λ = 5) weights. The connections were identified by applying the mask in Fig. 4A to the top 5% weights (by magnitude) from four models specific to males of HCP ((**A**) and (**E**)), females of HCP ((**B**) and (**F**)), males of GSP ((**C**) and (**G**)), and females of GSP ((**D**) and (**H**)). Each of the four models was obtained by averaging the corresponding 1,000 first-order models. The sex-specific lateralized connections consist of shared connections between male and female models ((**A**)-(**D**)), and the group (sex) "exclusive" connections ((**E**)-(**H**)). The weights of these shared connections show consistent sex differences. In female-specific models, the weights for connections involving the frontal lobe tend to be larger than those in male-specific models, especially for positive weights. Conversely, in male-specific models, the weights for connections to other lobes are generally larger than those in female-specific models. The "exclusive" connections in male-specific models are mostly inter-lobe and negative, whereas in female-specific models, they are mostly intra-lobe and positive. Statistics about these connections can be found in Fig. 7.

language processing and potentially influence connectivity patterns [67].

Regarding the MFG-IFG connection, proficiency in processing artificial grammar rules has been positively associated with functional connectivity between the left IFG and left MFG, highlighting sex-related variations in these cognitive functions [62, 68, 69]. Consistent with these findings, our framework identified MFG-IFG as a sex-specific lateralized connection. Moreover, this connection is linked with processing concessive and causal relationships, with differential effective connectivity patterns potentially modulated by sex-specific functional gradients within the inferior frontal gyrus (IFG) [70, 71]. Structural and functional variations within the dorsal attention network (DAN), particularly involving MTG, IPL, and the superior parietal lobule (SPL), further illustrate how these neural connections may adapt differently between males and females [72]. Additionally, meta-analytic evidence indicates lateralization differences within IFG subdivisions, with left IFG supporting classical language networks, and right IFG engaged in broader cognitive control [73].

The MFG-INS connection is associated with mild traumatic brain injury (mTBI) [74]. Our experimental findings underscore the need for personalized diagnostic and intervention strategies that consider both hemispheric and sex differences to enhance the effectiveness of mTBI treatment.

Collectively, these results suggest that sex-specific lateralization emerges from intricate interactions among structural connectivity gradients, functional network dynamics, and adaptive neurobiological mechanisms.

Sex differences: lateralization strength and lobe-level interactions

Sex differences in the strengths of shared lateralized connections: As reported in the results section, the first-order weights corresponding to these shared connections show consistent sex differences (Fig. 6, A to D). In our labeling strategy, "left" was labeled as 0 and "right" as 1. Therefore, a positive first-order weight indicates that stronger positive interactions (FC value approaching 1) between two ROIs suggest a higher probability of right lateralization. Conversely, a more negative interaction (FC value approaching -1) indicates that stronger negative interactions between two ROIs suggest a higher probability of left lateralization. The opposite interpretation applies to the negative first-order weights. Therefore, we can interpret the sex differences in the first-order weights for the shared connections as follows: *positive interactions involving the frontal lobes are more right-lateralized in females than in males, whereas positive interactions involving the temporal, parietal, insular, and limbic lobes are more left-lateralized in males than in females*. This observation of difference in strength of lateralization aligns with the findings on the lateralization patterns of right- and left-handed individuals [75], and supports neuroscience discoveries of shared functional network mechanisms across males and females [76, 43].

Sex differences in inter-/intra-lobe interaction patterns are identified by the "exclusive" lateralized connections, particularly within the frontal lobe (Fig. 6, E to H), a key region for language processing [77]. Using the same approach as above for interpreting first-order weights, we can summarize that *males have a stronger left lateralization in positive inter-lobe interactions, while females have a stronger right lateralization in positive intra-lobe interactions*. This divergence may result from the evolutionary pressure for lateralization, which optimizes functional organization and reduces redundancy among brain regions [78]. Inter-lobe connections, characterized by long-range wiring, are metabolically costly [79, 80, 81, 6], while the shorter-range intra-lobe connections are more energy-efficient. These intra-frontal connections may enhance language abilities in females. Conversely, male inter-lobe connections may be driven by the need to engage more extensive functional areas for complex visuospatial tasks. This divergence may contribute to sex differences in cognitive abilities, with males typically excelling in rightward visuospatial tasks and females in leftward verbal tasks [3, 82].

The observed sex differences in intra- and inter-lobe lateralized connectivity may be attributed to a combination of genetic, hormonal, and structural factors [7, 12, 53, 83, 84], which were discussed in the Background section. Estrogen in females enhances local synaptic plasticity and dendritic arborization, particularly in frontal regions, favoring specialized intra-lobe networks, while testosterone in males promotes long-range axonal growth and inter-lobe integration [13, 53, 83, 84]. Genetic factors, such as X-chromosome-linked genes, may further refine female-pattern intra-lobe connectivity through synapse regulation, whereas sex-specific epigenetic and neurodevelopmental pathways could prioritize cross-regional wiring in males [84, 85]. Developmental synaptic

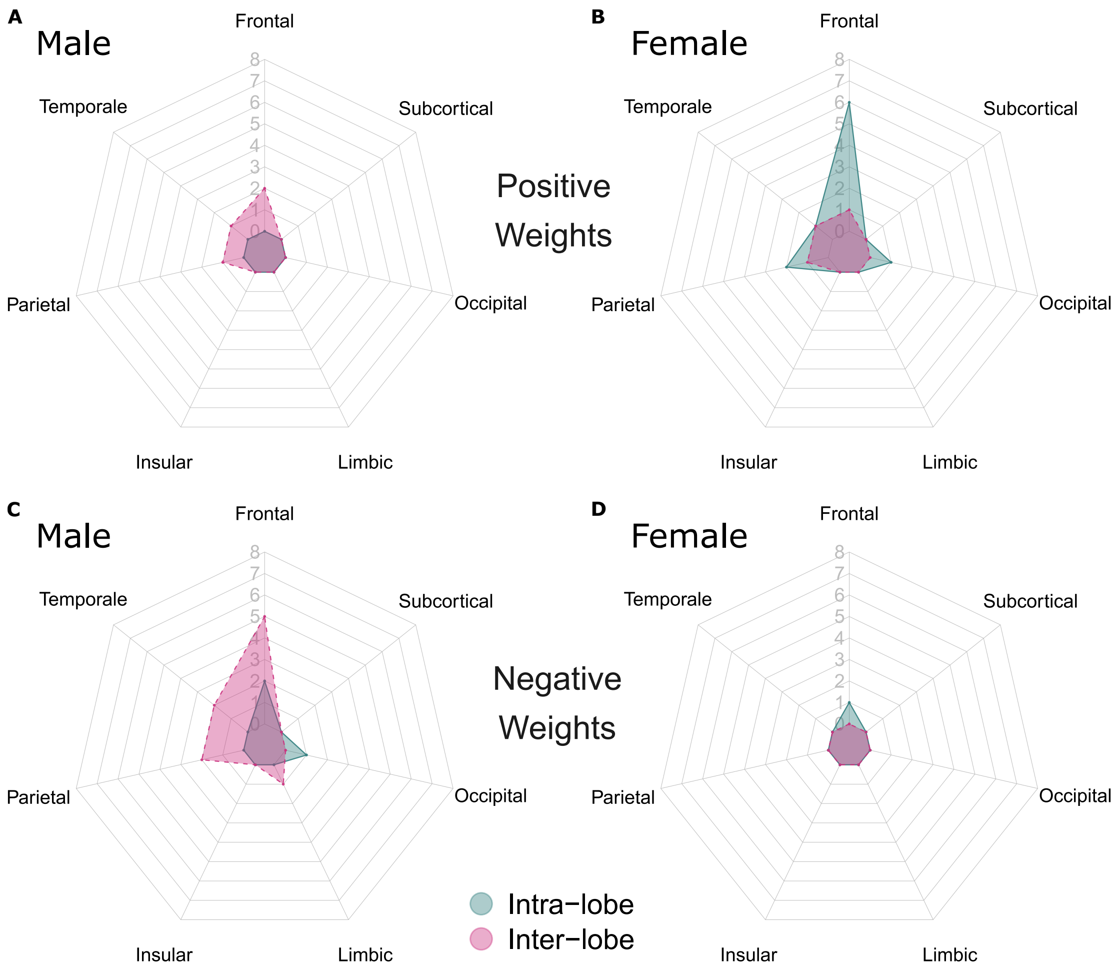

Figure 7. Count of the group "exclusive" lateralized connections for HCP and GSP (Fig. 6, E to H), categorized by associated lobes, inter- or intra-lobe, and signs of the first-order weights. The connections were identified by **(A)** the male-specific models with positive first-order weights; **(B)** the female-specific models with positive first-order weights; **(C)** the male-specific models with negative first-order weights; **(D)** the female-specific models with negative first-order weights. In male brain networks, 7 out of 10 "exclusive" connection counts are inter-lobe, with 71.4% of the inter-lobe connections having negative first-order weights. In female brain networks, 11 out of 13 "exclusive" connection counts are intra-lobe, with 91.7% of the intra-lobe connections having positive first-order weights.

pruning also differs: females retain more intra-lobe connections due to slower or later pruning, preserving modular processing, while males undergo earlier pruning to streamline inter-lobe efficiency. Structurally, higher gray matter density in female frontal lobes supports localized processing, whereas males exhibit greater long-range white matter tracts (e.g., superior longitudinal fasciculus) for inter-lobe communication [53, 79, 80, 83, 84]. Evolutionary pressures may have reinforced these patterns, with intra-lobe specialization in females aligning with social and detail-oriented tasks, and inter-lobe integration in males supporting spatial-motor coordination. These mechanisms, while probabilistic, interact with environmental and experiential factors, contributing to sex-specific cognitive strengths and vulnerabilities [86, 87].

Limitations

Our classification models were trained and tested within each dataset. To preserve meaningful features and explore whether consistent patterns could be identified across datasets, we did not apply harmonization. While our approach maintained the integrity of the original features, future research incorporating harmonized data and cross-dataset generalization could provide additional validation to further enhance the robustness and reliability of our findings.

Potential implications

Our study focused on sex as a grouping factor and utilized brain hemisphere labels to identify sex-specific lateralized patterns within human brain functional networks. The results demonstrate efficacy and stability in identifying and validating sex differences in lateralization. Importantly, the scope of this general predictive framework extends beyond its current application. One future direction for our proposed dual classification workflow involves predicting group-specific prognosis and treatment outcomes (e.g., changes in clinical, cognitive, or behavioral ratings) in psychiatric disorders, considering essential covariates such as age and sex [88, 89]. Previous studies, such as Chopra et al. (2024) [88], have aimed to minimize the impact of these covariates to enhance generalizability in predictive modeling. In contrast, our dual classification approach provides a complementary method for identifying biomarkers or predicting treatment responses specific to particular covariates, thus supporting precision medicine initiatives. Furthermore, our dual classification framework extends beyond sex-specific analyses and holds potential for uncovering subtype-specific neuroimaging biomarkers, offering valuable insights into personalized prognosis and treatment strategies across various psychiatric disorders [90]. Moreover, the GSDA algorithm can be adapted to combinations of grouping factors, such as sex and age groups. While this work primarily focused on classification, our method is adaptable to regression tasks, such as predicting behavioral cognitive scores or drug dosages.

Methods

Dual-classification with group-specific discriminant analysis (GSDA)

We propose a dual-classification framework with two primary objectives: learning group-specific models and identifying group-specific discriminant weights. For the first objective, specifically the classification of left vs. right brain hemi-

spheres, we train a linear classifier on the training data and then validate its performance on the test data. We refer to this process as the **first-order classification**. The weights derived from the model are called the **first-order weights**. Then we perform a second round of classification to identify the weights that show significant differences between group-specific models. Here, we train a linear classifier to differentiate between male- and female-specific models. This stage is called the **second-order classification**, and the associated weights are referred to as the **second-order weights**. This process is illustrated in stages ⑤ and ⑥ of Fig. 1A.

The first-order classification builds a (group-specific) prediction function. This function predicts whether an unseen brain hemisphere is left or right, based on a feature vector. These vectors represent the left or right human brain hemispheres and are extracted from the training neuroimaging data. The resulting prediction accuracy serves as a quantitative measure of the extent to which the learned lateralization patterns are generalized among the brain networks within the test set. The learned model weights can be interpreted as indicators of the significance or extent of differences between the corresponding connections of the left and right brain hemispheres.

The second-order classification is designed to identify weights that show significant differences between the male- and female-specific first-order models. In this stage, a linear classification model is trained on the first-order model weights to predict whether an unseen model is male- or female-specific. The features with larger weights in the second-order classification are considered to represent the stronger sex differences.

To learn group-specific models for the first-order classification, We propose a group-specific discriminant analysis (GSDA) algorithm

Problem formulation of GSDA

Let $(\mathbf{x}_i, y_i, \mathbf{g}_i)$ represent the ith sample, where $\mathbf{x}_i \in \mathcal{X} \subseteq \mathbb{R}^p$ denotes an input data vector, $y_i \in \mathcal{Y}$ denotes an output variable (label), and $\mathbf{g}_i \in \mathcal{G} \subseteq \mathbb{R}^q$ represents a covariate vector for the grouping factor(s). Here, $i \in [1, m]$, with m being the total number of samples. $\mathcal{X}$, $\mathcal{Y}$ and $\mathcal{G}$ are the feature spaces of the input data, output label, and grouping factor, respectively, with p and q as the corresponding feature dimensions for the input data $\mathbf{x}_i$ and grouping factor $\mathbf{g}_i$. In the context of this article, $\mathbf{x}_i$ is a feature vector that represents a brain hemisphere, y_i indicates whether $\mathbf{x}_i$ is the left or right hemisphere, and $\mathbf{g}_i$ is a binary (zero and one) indicator representing whether $\mathbf{x}_i$ is from a male or female subject (e.g. $g_i = 0$ for male and $g_i = 1$ for female). Assuming $\mathbf{x}_0 = 1$, considering $\mathbf{w}_0$ as the bias term, and denoting $\mathbf{w} \in \mathbb{R}^{p+1}$ as the vector of weights (coefficients) to be learned, with the target group represented as subscript $_\mathrm{t}$, we formulate the objective of learning group-specific models as follows:

$$\arg\max_{\mathbf{w}} \frac{1}{m_\mathrm{t}} \sum_{i=1}^{m_\mathrm{t}} \mathbb{P}(y_i|\mathbf{x}_i, \mathbf{w}) + \frac{\lambda}{m} \sum_{j=1}^{m} |\mathbb{P}(\mathbf{g}_j, \mathbf{w}^\top \mathbf{x}_j) - \mathbb{P}(\mathbf{g}_j)\mathbb{P}(\mathbf{w}^\top \mathbf{x}_j)|, \tag{1}$$

where m_t denotes the number of training samples from the target group, and $\lambda \geq 0$ is the hyperparameter that quantifies the importance of grouping factor(s) dependence. Based on Eq. (1), we formulate a general group-specific discriminant analysis (GSDA) framework as:

$$\arg\min_{\mathbf{w}} L(\mathbf{X}_\mathrm{t}^\top \mathbf{w}, \mathbf{y}_\mathrm{t}) + \alpha \|\mathbf{w}\|_K^2 - \lambda \underbrace{\rho(\mathbf{X}^\top \mathbf{w}, \mathbf{G})}_{\text{Group dependence}}, \tag{2}$$

where $L(\cdot, \cdot)$ denotes a classification or regression loss function, such as least square, logistic, or hinge, $\alpha \geq 0$ is the hyperparameter used for weight regularization, $\|\cdot\|_K^2$ denotes either an ℓ_1 or ℓ_2 regularization, with $K = 1$ or 2 respectively, $\mathbf{X}_\mathrm{t}$ denotes the target group's training samples, $\mathbf{X}$ denotes all training samples that consist of both target and non-target group samples, and $\rho(\cdot, \cdot)$ is a statistical dependence measure. In this work, we employed *Hilbert-Schmidt Independence Criterion (HSIC)* [91], a convex and smooth dependence measure. Given two sets $\mathbf{X} = \{\mathbf{x}_1, \mathbf{x}_2, \ldots, \mathbf{x}_m\}$ and $\mathbf{Y} = \{\mathbf{y}_1, \mathbf{y}_2, \ldots, \mathbf{y}_m\}$, both with size m, HSIC compute the statistical dependence between tests whether $\mathbf{X}$ and $\mathbf{Y}$ via

$$\rho_h(\mathbf{X}, \mathbf{Y}) = \frac{1}{(m-1)^2} \mathrm{tr}(\mathbf{KHLH}), \tag{3}$$

where $\mathbf{K}, \mathbf{H}, \mathbf{L} \in \mathbb{R}^{m\times m}$, $\mathbf{K}_{i,j} := k_x(\mathbf{x}_i, \mathbf{x}_j)$, $\mathbf{L}_{i,j} := k_y(\mathbf{y}_i, \mathbf{y}_j)$, $k_x(\cdot, \cdot)$ and $k_y(\cdot, \cdot)$ are two kernel functions, such as linear, polynomial, or radial basis function (RBF), $\mathbf{H} = \mathbf{I} - \frac{1}{m}\mathbf{1}\mathbf{1}^\top$ is the centering matrix, $\mathbf{I}$ is an identity matrix, and $\mathrm{tr}(\cdot)$ is the trace function. HSIC $\rho(\mathbf{X}, \mathbf{Y}) \geq 0$, and it is zero if and only if the two sets of variables $\mathbf{X}$ and $\mathbf{Y}$ are independent, i.e., $\mathbb{P}(\mathbf{x}, \mathbf{y}) = \mathbb{P}(\mathbf{x})\mathbb{P}(\mathbf{y})$. A higher HSIC value suggests stronger statistical dependence.

GSDA with logistic loss and maximum likelihood estimation

To maximize the likelihood of the target-group labels and the grouping factor(s) dependence as specified in Eq. (1), we adopt maximum likelihood estimation for optimizing the model weights $\mathbf{w}$. Here, we develop a novel algorithm, Group-Specific Discriminant Analysis with logistic loss (GSDA-Logit), as a variant of logistic regression for group-dependent learning. Let $\mathbb{P}(\mathbf{y}_\mathrm{t}|\mathbf{X}_\mathrm{t}, \mathbf{w})$ denote the likelihood of target labels $\mathbf{y}_\mathrm{t}$ given the model and target-group data $\mathbf{X}_\mathrm{t}$, $\mathbb{P}(\mathbf{w})$ be the prior probability of weights, assumed to follow a normal distribution $\mathcal{N}(0, \sigma^2)$, and $\mathbb{P}(\rho(\mathbf{X}^\top \mathbf{w}, \mathbf{G}))$ be the likelihood of grouping factor dependence, the overall likelihood $\mathcal{L}(\mathbf{w})$ to be maximized is as follows:

$$\begin{aligned}\mathcal{L}(\mathbf{w}) &= \mathbb{P}(\mathbf{y}_\mathrm{t}|\mathbf{X}_\mathrm{t}, \mathbf{w})\mathbb{P}(\mathbf{w})\mathbb{P}(\rho(\mathbf{X}^\top \mathbf{w}, \mathbf{G})) \\ &= \left(\prod_{i=1}^{m_\mathrm{t}} S(\mathbf{w}^\top \mathbf{x}_i)^{y_i} \left(1 - S(\mathbf{w}^\top \mathbf{x}_i)\right)^{(1-y_i)}\right) \\ &\quad \times \frac{1}{\sqrt{2\pi}\sigma} \exp\left(-\frac{\mathbf{w}^\top \mathbf{w}}{2\sigma^2}\right) S\left(\rho_h(\mathbf{w}^\top \mathbf{X}, \mathbf{G})\right),\end{aligned} \tag{4}$$

where $S(\cdot)$ denotes the logistic (or sigmoid) function, and $\mathbb{P}(\mathbf{w})$ can be interpreted as the ℓ_2 regularization for $\mathbf{w}$. Given that $\mathbf{w}^\top \mathbf{X}$ produces a row vector, Equation (3) can be reformulated as simplified HSIC [92]:

$$\begin{aligned}\rho_{sh}(\mathbf{w}^\top \mathbf{X}, \mathbf{G}) &= \mathrm{tr}((\mathbf{w}^\top \mathbf{X})^\top (\mathbf{w}^\top \mathbf{X})\mathbf{HLH}) \\ &= \mathbf{w}^\top \mathbf{XHLHX}^\top \mathbf{w},\end{aligned} \tag{5}$$

where $\mathbf{L} = \mathbf{G}^\top \mathbf{G}$. By replacing $\rho_h(\mathbf{w}^\top \mathbf{X}, \mathbf{G})$ with the simplified HSIC $\rho_{sh}(\mathbf{w}^\top \mathbf{X}, \mathbf{G})$, the likelihood can be rewritten as

$$\begin{aligned}\mathcal{L}(\mathbf{w}) &= \left(\prod_{i=1}^{m_\mathrm{t}} S(\mathbf{w}^\top \mathbf{x}_i)^{y_i} \left(1 - S(\mathbf{w}^\top \mathbf{x}_i)\right)^{(1-y_i)}\right) \\ &\quad \times \frac{1}{\sqrt{2\pi}\sigma} \exp\left(-\frac{\mathbf{w}^\top \mathbf{w}}{2\sigma^2}\right) S\left(\rho_{sh}(\mathbf{w}^\top \mathbf{X}, \mathbf{G})\right).\end{aligned} \tag{6}$$

The likelihood in Eq. (6) can be maximized using the same optimization steps for a standard logistic regression, i.e., computing the gradient of the negative log-likelihood. Let $\alpha = \frac{1}{\sigma^2}$ and λ denote the two hyperparameters that control the importance of the ℓ_2 regularization and grouping factor dependence regularization, respectively. Let $\mathcal{J}(\mathbf{w})$ denote the negative logarithm of the likelihood. Taking the gradient of $\mathcal{J}(\mathbf{w})$ with respect to $\mathbf{w}$, we obtain

$$\begin{aligned}\nabla \mathcal{J}(\mathbf{w}) =& \mathbf{X}_\mathrm{t}(S(\mathbf{X}_\mathrm{t}^\top \mathbf{w}) - \mathbf{y}_\mathrm{t}) + \alpha \mathbf{w} \\ &+ \lambda(S(\rho_{sh}(\mathbf{w}^\top \mathbf{X}, \mathbf{G})) - 1)\mathbf{XHLHX}^\top \mathbf{w}.\end{aligned} \tag{7}$$

Finally, $\mathbf{w}$ can be optimized iteratively via

$$\mathbf{w}^{k+1} = \mathbf{w}^k - \eta \nabla \mathcal{J}(\mathbf{w}^k), \tag{8}$$

where k denotes the kth iteration, η is the learning rate (step size). Algorithm 1 is the pseudocode for GSDA-Logit. In addition to standard gradient descent optimization, we have implemented the LBFGS algorithm [93] for faster optimization.

Theoretical interpretation for diverged test accuracy between groups

Here, we provide a theoretical analysis to interpret the accuracy divergence in Fig. 2A of a GSDA model on target-group and non-target-group data. Let $h \in \mathcal{H}$ be a hypothesis for predicting label y, where $\mathcal{H}$ is the hypothesis space. In the context of linear models with logistic loss in this article, a hypothesis

Algorithm 1 Group-Specific Discriminant Analysis with logistic loss (GSDA-Logit)

Input: Input data matrix $\mathbf{X} \in \mathbb{R}^{p\times m}$, target-group label vector $\mathbf{y}_t \in \mathbb{R}^{m_t}$, grouping factor(s), and indices of samples from the target group (optional, if not given, first $m_t < m$ samples are assumed to be the labeled target samples).
hyperparameters: α for ℓ_2 regularization, λ for group dependence (HSIC) regularization, and η for learning rate.
Output: Coefficient vector $\mathbf{w} \in \mathbb{R}^{p+1}$.

1: Encode the grouping factor(s) into a matrix $\mathbf{G} \in \mathbb{R}^{q\times m}$ ($q = 1$ for a binary grouping factor) using one-hot encoding. Then construct the kernel matrix $\mathbf{L} \in \mathbb{R}^{m\times m} = \mathbf{G}^\top\mathbf{G}$ and the centering matrix $\mathbf{H} \in \mathbb{R}^{m\times m}$;
2: Add a row of 1s to $\mathbf{X}$;
3: Randomly initialize $\mathbf{w}^k$ ($k = 0$);
4: **while** Not converge **do**
5: Compute gradient $\nabla\mathcal{J}(\mathbf{w})$ by Eq. (7);
6: Update $\mathbf{w}^{k+1} = \mathbf{w}^k - \eta\nabla\mathcal{J}(\mathbf{w}^k)$;
7: **end while**
8: **return** GSDA-Logit coefficient vector $\mathbf{w}$.

$h(\mathbf{x})$ is defined as

$$h(\mathbf{x}) = \begin{cases} 1, \text{ if } S(\mathbf{w}^\top\mathbf{x}) \geq 0.5, \\ 0, \text{ otherwise.} \end{cases} \tag{9}$$

Let the target group data be drawn from the distribution $\mathcal{D}_t$, then by a standard application of Vapnik-Chervonenkis (VC) theory [51], the bound on the generalization error for target-group data is

$$\epsilon_t(h) \leq \hat{\epsilon}_t(h) + O\left(\sqrt{\frac{d}{m}\ln\frac{m}{d}}\right), \tag{10}$$

where $\epsilon_t(h)$ denotes the generalization error for h on the target-group data, $\hat{\epsilon}_t(h)$ is the empirical generalization error for h on the target-group training examples, d represents the VC-dimension [51] of the hypothesis space $\mathcal{H}$, and $O(\cdot)$ denotes computational complexity. According to the domain adaptation theory [94], the upper bound on the generalization error for samples from the non-target group(s) is

$$\epsilon_{\backslash t}(h) \leq \hat{\epsilon}_t(h) + O\left(\sqrt{\frac{d}{m}\ln\frac{m}{d}}\right) + d_{\mathcal{H}}(\mathcal{D}_t, \mathcal{D}_{\backslash t}) + \Omega, \tag{11}$$

where $\mathcal{D}_{\backslash t}$ represents the distribution for non-target-group data, and $\Omega = \epsilon_t(h^*) + \epsilon_{\backslash t}(h^*)$, with $h^* = \arg\min_{h\in\mathcal{H}} \epsilon_t(h) + \epsilon_{\backslash t}(h)$ being the *ideal joint hypothesis* for target-group and non-target-group data. Ω is a constant for fixed data, and can be zero if h^* can accurately predict any sample from both $\mathcal{D}_t$ and $\mathcal{D}_{\backslash t}$. $d_{\mathcal{H}}(\mathcal{D}_t, \mathcal{D}_{\backslash t})$ is the $\mathcal{H}$-divergence, which measures the divergence between the target and non-target data distributions by

$$d_{\mathcal{H}}(\mathcal{D}_t, \mathcal{D}_{\backslash t}) = 2\sup_{h\in\mathcal{H}} \left|\mathbb{P}_{\mathcal{D}_t}[I(h)] - \mathbb{P}_{\mathcal{D}_{\backslash t}}[I(h)]\right|, \tag{12}$$

where $I(h)$ is an indicator function that $\mathbf{x} \in I(h) \Leftrightarrow h(\mathbf{x}) = 1$. Assume $g_i = 1$ if $\mathbf{x}_i \in \mathcal{D}_t$, and $g_i = 0$ if $\mathbf{x}_i \in \mathcal{D}_{\backslash t}$, the right-hand side of Eq. (12) can be rewritten as

$$\begin{aligned} \left|\mathbb{P}_{\mathcal{D}_t}[I(h)] - \mathbb{P}_{\mathcal{D}_{\backslash t}}[I(h)]\right| &= \left|\mathbb{P}(h(\mathbf{x}) = 1 \mid g = 1) - \mathbb{P}(h(\mathbf{x}) = 1 \mid g = 0)\right| \\ &= \left|\mathbb{P}(S(\mathbf{X}_t^\top\mathbf{w}) \geq 0.5) - \mathbb{P}(S(\mathbf{X}_{\backslash t}^\top\mathbf{w}) \geq 0.5)\right|, \end{aligned} \tag{13}$$

which can be viewed as the separability of transformed data $\mathbf{X}_t^\top\mathbf{w}$ and $\mathbf{X}_{\backslash t}^\top\mathbf{w}$ in the linear model context.

If we view the objective of maximizing dependence between $\mathbf{g}$ and $\mathbf{X}^\top\mathbf{w}$ in Eq. (1) as maximizing the corresponding mutual information, GSDA minimizes the uncertainty about the group labels $\mathbf{g}$ given the transformed data $\mathbf{X}^\top\mathbf{w}$. This enhances the separability of transformed data $\mathbf{X}_t^\top\mathbf{w}$ and $\mathbf{X}_{\backslash t}^\top\mathbf{w}$. From this perspective, we interpret group dependence as a form of regularization on the hypothesis space $\mathcal{H}$, ensuring that for any $h \in \mathcal{H}$, the transformed target-group and non-target-group data are separable. This results in a maximized $\mathcal{H}$-divergence in the generalization error bound for non-target-group data in Eq. (11). We can see that the difference between the generalization error bounds for target-group (Eq. (10)) and non-target-group data (Eq. (11)) is the $\mathcal{H}$-divergence term plus a constant. This directly translates to a lower generalization error bound or higher accuracy for target-group data over non-target-group data in the GSDA framework. Consequently, a theoretical gap exists between the expected accuracy achieved by a GSDA model on target group and non-target group data, with the expected accuracy for target group data being higher, i.e., GSDA models are more target-specific.

Group specificity index (GSI) for evaluating model group specificity

To measure the group specificity of discriminative models, we set the following criteria for a metric:

- Its value lies within $[0, 1]$.
- Its value equals 0 if the test accuracy for the target and non-target groups are identical.
- When the test accuracy of the target and non-target groups differ, the value of this metric should be proportional to 1) the absolute accuracy for the target group, and 2) the closeness of accuracy for the non-target group to the random chance.
- Greater relative accuracy divergence between target and non-target groups will result in a higher value of this metric.

To satisfy the above conditions, we propose a Group Specificity Index (GSI) for binary classification problems as follows:

$$\mathrm{GSI} = 2\mathrm{BAT}(\mathrm{BAT} - 0.5 - |\mathrm{BANT} - 0.5|), \tag{14}$$

where $\mathrm{BAT} \in [0.5, 1]$ and $\mathrm{BANT} \in [0, 1]$ represent the balanced accuracy of the target group and non-target group data, respectively. Balanced accuracy is chosen to mitigate the impact of imbalanced samples. It is defined as $\mathrm{BA} = (\mathrm{TPR} + \mathrm{TNR})/2$, where the true positive rate $\mathrm{TPR} = \{\text{number of true positives}\}/\{\text{number of total positives}\}$ and the true negative rate $\mathrm{TNR} = \{\text{number of true negatives}\}/\{\text{number of total negatives}\}$. In the left vs. right brain hemisphere classification problem, the numbers of left and right training examples are equal, making balanced accuracy equivalent to accuracy. The expression $|\mathrm{BANT} - 0.5|$ measures how close the accuracy of the non-target group is to random chance (0.5), and $\mathrm{BAT} - 0.5 - |\mathrm{BANT} - 0.5|$ quantifies the relative accuracy divergence between the target and non-target groups. Since we are interested in generalized lateralization patterns for the target group, models that perform worse than random chance on target test sets are not considered.

Resting-State fMRI data and processing

We use resting-state fMRI data from the Human Connectome Project (HCP) [45] and the Genomics Superstruct Project (GSP) [46] for brain hemisphere classification to study lateralization. Table 2 summarizes the demographic information of the subjects involved in our experiments across both datasets.

Human Connectome Project (HCP)

Acquisition. All MRI data were collected using the same 3T Siemens Skyra magnetic resonance machines at Washington University in St. Louis with a 32-channel head coil [95]. Specifically, rs-fMRI was acquired using a gradient-echo echo-planar imaging (GE-EPI) sequence with the following parameters: repetition time (TR) = 720 ms, echo time (TE) = 33.1 ms, flip angle (FA) = 52°, bandwidth = 2290 Hz/pixel, field of view (FOV) = 208×180 mm^2, matrix = 104×90, voxel size = $2 \times 2 \times 2$ mm^3, multi-band acceleration factor = 8, slices = 72, and total scan time of 1200 frames = 14 min and 24 s [45]. During the scan, participants were asked to open their eyes and stare at a white cross on a screen with a black background. There were two rs-fMRI sessions (REST1 and REST2) acquired on two consecutive days, each including two runs with a left-to-right (LR) and a right-to-left (RL) phase encoding direction. The T1-weighted images were acquired by using a magnetized rapid gradient-echo

Table 2. Information of HCP and GSP dataset used for the experiments, where "M" denotes male and "F" denotes female for sex, "L" denotes left-handedness, "R" denotes right-handedness, and "A" denotes ambidexterity for handedness, and "SD" denotes standard deviation.

Dataset	Num. of Subjects	Sex (M/F)	Handedness (L/R/A)	Average Age (SD) Total	Male	Female	Num. of Sessions
HCP [45]	960	445/515	85/875/0	28.7 (3.71)	27.9 (3.69)	29.4 (3.59)	2
GSP [46]	1570	665/905	110/1449/11	21.5 (2.89)	21.6 (3.04)	21.5 (2.78)	1

imaging (MPRAGE) sequence with the following parameters: TR = 2,400 ms, TE = 2.14 ms, reversal time (TI) = 1,000 ms, FA = 8°, FOV = 224×224 mm^2, voxel size 0.7 mm isotropic, and total scan time = 7 min and 40 s.

Preprocessing. We follow the same steps in [96] for HCP data preprocessing. The HCP minimal preprocessing pipeline (version 2.0) was utilized, including magnetic gradient distortion correction, EPI distortion correction, non-brain tissue removal, Montreal Neurological Institute (MNI) standard space registration, and intensity normalization. The resultant data were denoised using independent component analysis (ICA) with the FIX tool [97], which identifies and eliminates spatiotemporal signal components from non-neuronal or structural noise, with an emphasis on head movement. Subsequently, five post-processing steps were applied to the minimally preprocessed data: 1) spatial smoothing with 4 mm FWHM kernel, twice the voxel resolution of HCP fMRI data; 2) linear detrending to minimize the effects of low-frequency drift; 3) regression of a suite of nuisance variables unrelated to neural signals, such as average signals from white matter (WM) and cerebrospinal fluid (CSF), as well as the whole brain (global signal, GS); 4) bandpass filtering (0.01Hz-0.1Hz); 5) scrubbing to control effects of transient movement across the time series frames.

Genomics Superstruct Project (GSP)

Acquisition. All imaging data were collected on matched 3T Tim Trio scanners (Siemens Healthcare, Erlangen, Germany) at Harvard University and Massachusetts General Hospital using the vendor-supplied 12-channel phased-array head coil [46]. Structural data included a high-resolution (1.2 mm isotropic) multi-echo T1-weighted magnetization-prepared gradient-echo image. Functional imaging data were acquired using a GE-EPI sequence sensitive to blood oxygenation level-dependent (BOLD) contrast with the following parameters: TR = 3,000 ms, TE = 30 ms, FA = 85°, voxel size = $3 \times 3 \times 3$ mm^3, slices = 47, and total scan time of 124 frames = 6 min and 12 s.

Preprocessing. Same as HCP data preprocessing, we follow [96] to preprocess GSP data. SPM preprocessed all fMRI data [98] and GRETNA [99] toolkit, including the following steps: 1) removing the first four volumes to assure that the magnetization is at steady state; 2) slice-timing correction; 3) realignment of all volumes to the first volume to reduce the effects of head motion; 4) co-registration of GE-EPI data to the native, cropped, high-resolution structural image and then normalizing them to the MNI space through Diffeomorphic Anatomical Registration Through Exponentiated Lie Algebra (DARTEL) algorithm; 5) spatial smoothing with a 6 mm FWHM kernel, twice the voxel resolution of GSP fMRI data; 6) linear detrending to minimize the effects of low-frequency drift; 7) six head motion parameters regression, as well as the WM, CSF and GS; 8) lowpass filtering (<0.08Hz) [100].

Extracting intrahemispheric brain network

We use intrahemispheric brain network connectivity as features to represent brain hemispheres. Figure 1A ①-③ illustrates the data processing workflow for obtaining intrahemispheric connections from resting-state time series. Time sequences were extracted using the Brainnetome atlas (BNA) [49], which divides the human brain into 246 regions (123 per hemisphere). Pearson correlation was computed to represent the connectivity between brain regions. Following Liang *et al.* [101], the correlation coefficients were transformed into z-scores using Fisher's z transform. For HCP data, we averaged z-scores across the RL and LR runs for each session. To extract half-brain features, we reordered the columns and rows of the connectivity matrix to produce two 123×123 matrices, representing the intrahemispheric networks for the two brain hemispheres of each subject. We then extracted the upper triangle of these matrices (illustrated as the red and blue areas in ③ of Fig. 1) to form two 7503-dimensional feature vectors by BNA for the two hemispheres for experiments.

Experimental setting

Multivariate classification algorithm setup

For all multivariate methods, the classification problem is binary: left brain hemispheres are labeled as 0, and right brain hemispheres are labeled as 1. For GSDA-Logit, sex is utilized as the grouping factor in the experiments, encoding males as 0 and females as 1. Given the binary nature of the grouping factor, the matrix $\mathbf{G}$ simplifies to a vector $\mathbf{g}$ in this experiment. For first-order classification with group-specific model training, we set the regularization parameter $\alpha = 0.1$ and varied $\lambda \in [0, 1.0, 2.0, 5.0, 8.0, 10.0]$ for GSDA-Logit, where α controls ℓ_2 regularization, and λ regulates statistical dependence on grouping factors. When $\lambda = 0$, GSDA-Logit degenerates to standard logistic regression, as it does not incorporate grouping factor dependence in optimizing model weights. To learn first-order multivariate control models and conduct second-order classification, we employed a logistic regression classifier implemented in scikit-learn [102] with default hyperparameters.

Cross-validation strategy

First-order classification setting. We implemented two cross-validation strategies for left vs. right brain hemisphere classification:

i. Within each dataset, subjects were randomly divided into two equal groups (50% each). The training set consisted of left hemispheres from the first group and right hemispheres from the second group, while the remaining hemispheres (right hemispheres of the first group and left hemispheres of the second group) were used for testing. This setup ensured that no subject contributed both hemispheres to the training set, minimizing potential biases from intra-subject correlations, as illustrated in ④ of Fig. 1A. The corresponding results are reported in Fig. 2, and Fig S. 2A and 2B.

ii. To further validate our findings, we employed an alternative strategy in which 20% of subjects were held out entirely as an additional unseen test set. The training examples were obtained by applying the same strategy above to the remaining 80% of subjects.

Each cross-validation strategy was repeated 1,000 times, generating 1,000 models per learning task. For the HCP dataset, which includes two scanning sessions per subject on different days, the session not used for training served as an additional test set.

Second-order classification setting. Using the first-order models learned for each task, we perform second-order classification through the following steps:

i. Define a classification problem of interest, for example, male-specific GSDA models trained on HCP with $\alpha = 0.1$, $\lambda = 5$ vs . female-specific GSDA models trained on HCP with $\alpha = 0.1$, $\lambda = 5$, with 1,000 models for each group.

ii. Split the 2,000 models into 80% training and 20% test sets by stratified random sampling.

iii. Train a standard logistic regression classifier using the scikit-learn [102] implementation with default setting on the training set and then evaluate the performance on the test set.

iv. Repeat steps 2 and 3 with different random seeds for 1,000 splits of training and test sets.

Data availability

All additional supporting data are available in the GigaScience repository, GigaDB [103].

Generalist Repository

There are additional data files hosted in Zenodo archives:

https://doi.org/10.5281/zenodo.10050233 [104]

https://doi.org/10.5281/zenodo.10050234 [105]

GitHub repository

The software code is available from GitHub repository: https://github.com/shuo-zhou/GSDA-Lateralization [106]

A version of record snapshot of the GitHub repository has been archived in the Software Heritage Library [107] with the PID swh:1:snp:495f818df0e3c6d9ac1898b1cc14ec0ea396d98a.

Declarations

List of abbreviations

BNA, Brainnetome atlas; BOLD, blood oxygenation level-dependent; CG, cingulate gyrus; DARTEL, Diffeomorphic Anatomical Registration Through Exponentiated Lie Algebra; FA, flip angle; FOV, field of view; GE-EPI, gradient-echo echo-planar imaging; GSDA, group-specific discriminant analysis; GSI, group specificity index; GSP, Brain Genomics Superstruct Project; HCP, Human Connectome Project; HSIC, Hilbert-Schmidt Independence Criterion; ICA, independent component analysis; IFG, inferior frontal gyrus; INS, insular gyrus; ITG, inferior temporal gyrus; IPL, inferior parietal lobe; OrG, orbital gyrus; PCL, paracentral lobule; Pcun, precuneus; PoG, postcentral gyrus; PrG, precentral gyrus; LOcC: lateral occipital cortex; MFG, middle frontal gyrus; MTG, middle temporal gyrus; MVOcC: medioVentral occipital cortex; SFG, superior frontal gyrus; STG, superior temporal gyrus; TR, repetition time; TE, echo time; WM, white matter.

Consent for publication

Not applicable

Competing Interests

The authors declare that they have no competing interests.

Funding

This work was supported by the STI 2030-Major Projects (2021ZD0200500, 2021ZD0201701), the National Natural Science Foundation of China (T2325006, 82021004), and the Fundamental Research Funds for the Central Universities (2233200020).

Author's Contributions

Conceptualization: S.Z., J.L., H.L., and G.G. Methodology: S.Z, J.L., H.W., H.L., and G.G. Investigation: S.Z., and J.L. Resources: Y.J. Visualization: S.Z., and J.L. Software: S.Z. Writing—original draft: S.Z. and J.L. Writing—review and editing: S.Z., J.L., Y.J., H.W., H.L., and G.G. Supervision: H.L., and G.G. Funding acquisition: G.G.

References

1. Ojemann G, Ojemann J, Lettich E, Berger M. Cortical language localization in left, dominant hemisphere: an electrical stimulation mapping investigation in 117 patients. Journal of Neurosurgery 1989;71(3):316–326.
2. Gazzaniga MS. Cerebral specialization and interhemispheric communication: does the corpus callosum enable the human condition? Brain 2000;123(7):1293–1326.
3. Levy J. Lateral specialization of the human brain, behavioral manifestations and possible evolutionary basis. The Biology of Behavior 1972;.
4. Hirad AA, Bazarian JJ, Merchant-Borna K, Garcea FE, Heilbronner S, Paul D, et al. A common neural signature of brain injury in concussion and subconcussion. Science advances 2019;5(8):eaau3460.
5. Neubauer S, Gunz P, Scott NA, Hublin JJ, Mitteroecker P. Evolution of brain lateralization: A shared hominid pattern of endocranial asymmetry is much more variable in humans than in great apes. Science Advances 2020;6(7):eaax9935.
6. He X, Caciagli L, Parkes L, Stiso J, Karrer TM, Kim JZ, et al. Uncovering the biological basis of control energy: Structural and metabolic correlates of energy inefficiency in temporal lobe epilepsy. Science Advances 2022;8(45):eabn2293.
7. Toga AW, Thompson PM. Mapping brain asymmetry. Nature Reviews Neuroscience 2003;4(1):37–48.
8. Clements A, Rimrodt S, Abel J, Blankner J, Mostofsky S, Pekar J, et al. Sex differences in cerebral laterality of language and visuospatial processing. Brain and Language 2006;98(2):150–158.
9. Sommer IE, Aleman A, Somers M, Boks MP, Kahn RS. Sex differences in handedness, asymmetry of the planum temporale and functional language lateralization. Brain Research 2008;1206:76–88.
10. Agcaoglu O, Miller R, Mayer AR, Hugdahl K, Calhoun VD. Lateralization of resting state networks and relationship to age and gender. NeuroImage 2015;104:310–325.
11. Reber J, Tranel D. Sex differences in the functional lateralization of emotion and decision making in the human brain. Journal of Neuroscience Research 2017;95(1-2):270–278.
12. Hirnstein M, Hugdahl K, Hausmann M. Cognitive sex differences and hemispheric asymmetry: A critical review of 40 years of research. Laterality: Asymmetries of Body, Brain and Cognition 2019;24(2):204–252.
13. Migeon BR. Why females are mosaics, X-chromosome inactivation, and sex differences in disease. Gender medicine 2007;4(2):97–105.
14. Chura LR, Lombardo MV, Ashwin E, Auyeung B, Chakrabarti B, Bullmore ET, et al. Organizational effects of fetal testosterone on human corpus callosum size and asymmetry. Psychoneuroendocrinology 2010;35(1):122–132.
15. Geschwind N, Galaburda AM. Cerebral lateralization: Biological mechanisms, associations, and pathology: III. A hypothesis and a program for research. Archives of neurology 1985;42(7):634–654.
16. Kim HJ, Casadesus G. Estrogen-mediated effects on cognition and synaptic plasticity: what do estrogen receptor knockout models tell us? Biochimica et Biophysica Acta (BBA)-General Subjects 2010;1800(10):1090–1093.
17. Bi R, Foy MR, Vouimba RM, Thompson RF, Baudry M. Cyclic changes in estradiol regulate synaptic plasticity through the MAP kinase pathway. Proceedings of the National Academy of Sciences 2001;98(23):13391–13395.
18. Flynn JM, Dimitrijevich SD, Younes M, Skliris G, Murphy LC, Cammarata PR. Role of wild-type estrogen receptor-β in mitochondrial cytoprotection of cultured normal male and female human lens epithelial cells. American Journal of Physiology-Endocrinology and Metabolism 2008;295(3):E637–E647.
19. McEwen B. Estrogen actions throughout the brain. Recent progress in hormone research 2002;57:357–384.
20. Woolley CS. Estrogen-mediated structural and functional synaptic plasticity in the female rat hippocampus. Hormones and behavior 1998;34(2):140–148.
21. Ruigrok AN, Salimi-Khorshidi G, Lai MC, Baron-Cohen S, Lombardo MV, Tait RJ, et al. A meta-analysis of sex differences in human brain structure. Neuroscience & Biobehavioral Reviews 2014;39:34–50.
22. Ingalhalikar M, Smith A, Parker D, Satterthwaite TD, Elliott MA, Ruparel K, et al. Sex differences in the structural connectome of the human brain. Proceedings of the National Academy of Sciences 2014;111(2):823–828.
23. Grabowska A. Sex on the brain: are gender-dependent structural and functional differences associated with behavior? Journal of neuroscience research 2017;95(1-2):200–212.
24. You W, Li Q, Chen L, He N, Li Y, Long F, et al. Common and distinct cortical thickness alterations in youth with autism spectrum disorder and attention-deficit/hyperactivity disorder. BMC medicine 2024;22(1):92.
25. Gui Y, Zhou X, Wang Z, Zhang Y, Wang Z, Zhou G, et al. Sex-specific genetic association between psychiatric disorders and cognition, behavior and brain imaging in children and adults. Translational Psychiatry 2022;12(1):347.

26. Grimm S, Beck J, Schuepbach D, Hell D, Boesiger P, Bermpohl F, et al. Imbalance between left and right dorsolateral prefrontal cortex in major depression is linked to negative emotional judgment: an fMRI study in severe major depressive disorder. Biological psychiatry 2008;63(4):369–376.
27. Okada N, Fukunaga M, Miura K, Nemoto K, Matsumoto J, Hashimoto N, et al. Subcortical volumetric alterations in four major psychiatric disorders: a mega-analysis study of 5604 subjects and a volumetric data-driven approach for classification. Molecular Psychiatry 2023;28(12):5206–5216.
28. Jansen A, Menke R, Sommer J, Förster AF, Bruchmann S, Hempleman J, et al. The assessment of hemispheric lateralization in functional MRI—robustness and reproducibility. NeuroImage 2006;33(1):204–217.
29. Kinsbourne M. Hemineglect and hemisphere rivalry. Advances in Neurology 1977;18:41–49.
30. Kolb B, Whishaw IQ. Fundamentals of Human Neuropsychology. Macmillan; 2009.
31. Fox MD, Raichle ME. Spontaneous fluctuations in brain activity observed with functional magnetic resonance imaging. Nature Reviews Neuroscience 2007;8(9):700–711.
32. Logothetis NK. What we can do and what we cannot do with fMRI. Nature 2008;453(7197):869–878.
33. Sporns O, Tononi G, Kötter R. The human connectome: a structural description of the human brain. PLoS Computational Biology 2005;1(4):e42.
34. Passingham RE, Stephan KE, Kötter R. The anatomical basis of functional localization in the cortex. Nature Reviews Neuroscience 2002;3(8):606–616.
35. Smith SM, Miller KL, Moeller S, Xu J, Auerbach EJ, Woolrich MW, et al. Temporally-independent functional modes of spontaneous brain activity. Proceedings of the National Academy of Sciences 2012;109(8):3131–3136.
36. Finn ES, Shen X, Scheinost D, Rosenberg MD, Huang J, Chun MM, et al. Functional connectome fingerprinting: identifying individuals using patterns of brain connectivity. Nature Neuroscience 2015;18(11):1664–1671.
37. Zuo XN, Kelly C, Di Martino A, Mennes M, Margulies DS, Bangaru S, et al. Growing together and growing apart: regional and sex differences in the lifespan developmental trajectories of functional homotopy. Journal of Neuroscience 2010;30(45):15034–15043.
38. Seghier ML. Laterality index in functional MRI: methodological issues. Magnetic Resonance Imaging 2008;26(5):594–601.
39. Ito KL, Liew SL. Calculating the laterality index using FSL for stroke neuroimaging data. GigaScience 2016;5(suppl_1):s13742–016.
40. Friston KJ. Functional and effective connectivity: a review. Brain Connectivity 2011;1(1):13–36.
41. Jacobsen LK, Slotkin TA, Mencl WE, Frost SJ, Pugh KR. Gender-specific effects of prenatal and adolescent exposure to tobacco smoke on auditory and visual attention. Neuropsychopharmacology 2007;32(12):2453–2464.
42. Schwarz E, Guest PC, Rahmoune H, Wang L, Levin Y, Ingudomnukul E, et al. Sex-specific serum biomarker patterns in adults with Asperger's syndrome. Molecular Psychiatry 2011;16(12):1213–1220.
43. Cui Z, Su M, Li L, Shu H, Gong G. Individualized prediction of reading comprehension ability using gray matter volume. Cerebral Cortex 2018;28(5):1656–1672.
44. Good CD, Johnsrude I, Ashburner J, Henson RN, Friston KJ, Frackowiak RS. Cerebral asymmetry and the effects of sex and handedness on brain structure: a voxel-based morphometric analysis of 465 normal adult human brains. NeuroImage 2001;14(3):685–700.
45. Smith SM, Beckmann CF, Andersson J, Auerbach EJ, Bijsterbosch J, Douaud G, et al. Resting-state fMRI in the human connectome project. NeuroImage 2013;80:144–168.
46. Holmes AJ, Hollinshead MO, O'keefe TM, Petrov VI, Fariello GR, Wald LL, et al. Brain Genomics Superstruct Project initial data release with structural, functional, and behavioral measures. Scientific Data 2015;2(1):1–16.
47. Sullivan GM, Feinn R. Using effect size—or why the P value is not enough. Journal of Graduate Medical Education 2012;4(3):279–282.
48. Serdar CC, Cihan M, Yücel D, Serdar MA. Sample size, power and effect size revisited: simplified and practical approaches in pre-clinical, clinical and laboratory studies. Biochemia Medica 2021;31(1):27–53.
49. Fan L, Li H, Zhuo J, Zhang Y, Wang J, Chen L, et al. The human brainnetome atlas: a new brain atlas based on connectional architecture. Cerebral Cortex 2016;26(8):3508–3526.
50. Diestel R. Graph Theory. New York: Springer; 2000.
51. Vapnik VN. Statistical Learning Theory. Wiley New York; 1998.
52. Smith DV, Utevsky AV, Bland AR, Clement N, Clithero JA, Harsch AE, et al. Characterizing individual differences in functional connectivity using dual-regression and seed-based approaches. NeuroImage 2014;95:1–12.
53. Zhang C, Dougherty CC, Baum SA, White T, Michael AM. Functional connectivity predicts gender: Evidence for gender differences in resting brain connectivity. Human Brain Mapping 2018;39(4):1765–1776.
54. Kong R, Li J, Orban C, Sabuncu MR, Liu H, Schaefer A, et al. Spatial topography of individual-specific cortical networks predicts human cognition, personality, and emotion. Cerebral Cortex 2019;29(6):2533–2551.
55. Onitsuka T, Shenton ME, Salisbury DF, Dickey CC, Kasai K, Toner SK, et al. Middle and inferior temporal gyrus gray matter volume abnormalities in chronic schizophrenia: an MRI study. American Journal of Psychiatry 2004;161(9):1603–1611.
56. Koyama MS, O'Connor D, Shehzad Z, Milham MP. Differential contributions of the middle frontal gyrus functional connectivity to literacy and numeracy. Scientific Reports 2017;7(1):17548.
57. Lynch JC. The functional organization of posterior parietal association cortex. Behavioral and Brain Sciences 1980;3(4):485–499.
58. Hyva J, et al. Regional distribution of functions in parietal association area 7 of the monkey. Brain Research 1981;206(2):287–303.
59. Greenlee JD, Oya H, Kawasaki H, Volkov IO, Severson III MA, Howard III MA, et al. Functional connections within the human inferior frontal gyrus. Journal of Comparative Neurology 2007;503(4):550–559.
60. Kortz MW, Lillehei KO. Insular Cortex. StatPearls 2021;.
61. Duncan J. The multiple-demand (MD) system of the primate brain: mental programs for intelligent behaviour. Trends in Cognitive Sciences 2010;14(4):172–179.
62. Kansaku K, Yamaura A, Kitazawa S. Sex differences in lateralization revealed in the posterior language areas. Cerebral Cortex 2000;10(9):866–872.
63. Cole MW, Ito T, Bassett DS, Schultz DH. Activity flow over resting-state networks shapes cognitive task activations. Nature Neuroscience 2016;19(12):1718–1726.
64. van Dokkum LE, Gasser SM, Deverdun J, Herbet G, Mura T, d'Agata B, et al. Resting state network plasticity related to picture naming in low-grade glioma patients before and after resection. NeuroImage: Clinical 2019;24:102010.
65. Laws KR. Sex differences in lexical size across semantic categories. Personality and Individual Differences 2004;36(1):23–32.
66. Ala-Salomäki H, Kujala J, Liljeström M, Salmelin R. Picture naming yields highly consistent cortical activation patterns: Test–retest reliability of magnetoencephalography recordings. NeuroImage 2021;227:117651.
67. Li S, Jiang X, Yu H, Zhou X. Cognitive empathy modulates the processing of pragmatic constraints during sentence comprehension. Social cognitive and affective neuroscience 2014;9(8):1166–1174.
68. Lotz A, Kinder A. Rapid communication: Sex differences in artificial grammar learning: Evidence for different strategies in men and women. Quarterly Journal of Experimental Psychology 2011;64(3):417–424.
69. Kepinska O, de Rover M, Caspers J, Schiller NO. Connectivity of the hippocampus and Broca's area during acquisition of a novel grammar. NeuroImage 2018;165:1–10.
70. Xu X, Yu H, Gao X, Shen B, Feng W, Zhou X. Understanding an implicated causality: The brain network for processing concessive relations. Brain and Language 2022;234:105177.
71. Nakae T, Matsumoto R, Kunieda T, Arakawa Y, Kobayashi K, Shimotake A, et al. Connectivity gradient in the human left inferior frontal gyrus: intraoperative cortico-cortical evoked potential study. Cerebral Cortex 2020;30(8):4633–4650.
72. Wu H, Song Y, Yang X, Chen S, Ge H, Yan Z, et al. Functional and structural alterations of dorsal attention network in preclinical and early-stage

Alzheimer's disease. CNS Neuroscience & Therapeutics 2023;29(6):1512–1524.
73. Bulut T. Functional connectivity of the inferior frontal gyrus: A meta-analytic connectivity modeling study. BioRxiv 2022;p. 2022–02.
74. Li F, Liu Y, Lu L, Li H, Xing C, Chen H, et al. Causal interactions with an insular-cortical network in mild traumatic brain injury. European Journal of Radiology 2022;157:110594.
75. Vingerhoets G, Acke F, Alderweireldt AS, Nys J, Vandemaele P, Achten E. Cerebral lateralization of praxis in right-and left-handedness: Same pattern, different strength. Human Brain Mapping 2012;33(4):763–777.
76. Linnman C, Beucke JC, Jensen KB, Gollub RL, Kong J. Sex similarities and differences in pain-related periaqueductal gray connectivity. Pain 2012;153(2):444–454.
77. Tuckute G, Paunov A, Kean H, Small H, Mineroff Z, Blank I, et al. Frontal language areas do not emerge in the absence of temporal language areas: A case study of an individual born without a left temporal lobe. Neuropsychologia 2022;169:108184.
78. Vallortigara G, Rogers L. Survival with an asymmetrical brain: advantages and disadvantages of cerebral lateralization. Behavioral and Brain Sciences 2005;.
79. Ringo JL, Doty RW, Demeter S, Simard PY. Time is of the essence: a conjecture that hemispheric specialization arises from interhemispheric conduction delay. Cerebral Cortex 1994;4(4):331–343.
80. Laughlin SB, Sejnowski TJ. Communication in neuronal networks. Science 2003;301(5641):1870–1874.
81. Lennie P. The cost of cortical computation. Current Biology 2003;13(6):493–497.
82. Levy J. Lateral differences in the human brain in cognition and behavioral control. In: Buser P, Rougeul-Buser A, editors. Cerebral Correlates of Conscious Experience North Holland New York; 1978.p. 285–298.
83. Hodgetts S, Hausmann M. Sex/gender differences in brain lateralisation and connectivity. In: Sex Differences in Brain Function and Dysfunction Springer; 2022.p. 71–99.
84. Alfano V, Cavaliere C, Di Cecca A, Ciccarelli G, Salvatore M, Aiello M, et al. Sex differences in functional brain networks involved in interoception: An fMRI study. Frontiers in Neuroscience 2023;17:1130025.
85. Hjelmervik H, Hausmann M, Osnes B, Westerhausen R, Specht K. Resting states are resting traits–an FMRI study of sex differences and menstrual cycle effects in resting state cognitive control networks. PloS one 2014;9(7):e103492.
86. Bonelli C, Mancuso L, Manuello J, Liloia D, Costa T, Cauda F. Sex differences in brain homotopic co-activations: a meta-analytic study. Brain Structure and Function 2022;227(8):2839–2855.
87. Shirzadi S, Dadgostar M, Einalou Z, Erdoğan SB, Akin A. Sex based differences in functional connectivity during a working memory task: an fNIRS study. Frontiers in Psychology 2024;15:1207202.
88. Chopra S, Dhamala E, Lawhead C, Ricard JA, Orchard ER, An L, et al. Generalizable and replicable brain-based predictions of cognitive functioning across common psychiatric illness. Science Advances 2024;10(45):eadn1862.
89. Long F, Chen Y, Zhang Q, Li Q, Wang Y, Wang Y, et al. Predicting treatment outcomes in major depressive disorder using brain magnetic resonance imaging: a meta-analysis. Molecular Psychiatry 2024;p. 1–13.
90. Luo L, You W, DelBello MP, Gong Q, Li F. Recent advances in psychoradiology. Physics in Medicine & Biology 2022;67(23):23TR01.
91. Gretton A, Bousquet O, Smola A, Schölkopf B. Measuring statistical dependence with Hilbert-Schmidt norms. In: International Conference on Algorithmic Learning Theory; 2005. p. 63–77.
92. Zhou S, Li W, Cox C, Lu H. Side information dependence as a regularizer for analyzing human brain conditions across cognitive experiments. In: AAAI Conference on Artificial Intelligence; 2020. p. 6957–6964.
93. Liu DC, Nocedal J. On the limited memory BFGS method for large scale optimization. Mathematical Programming 1989;45(1):503–528.
94. Ben-David S, Blitzer J, Crammer K, Pereira FC. Analysis of Representations for Domain Adaptation. In: Advances in Neural Information Processing Systems, vol. 19; 2006. p. 137–144.
95. Van Essen DC, Smith SM, Barch DM, Behrens TE, Yacoub E, Ugurbil K, et al. The WU-Minn human connectome project: an overview. NeuroImage 2013;80:62–79.
96. Peng S, Xu P, Jiang Y, Gong G. Activation network mapping for integration of heterogeneous fMRI findings. Nature Human Behaviour 2022;6(10):1417–1429.
97. Salimi-Khorshidi G, Douaud G, Beckmann CF, Glasser MF, Griffanti L, Smith SM. Automatic denoising of functional MRI data: combining independent component analysis and hierarchical fusion of classifiers. NeuroImage 2014;90:449–468.
98. Friston KJ. Statistical parametric mapping. Neuroscience Databases: A Practical Guide 2003;p. 237–250.
99. Wang J, Wang X, Xia M, Liao X, Evans A, He Y. GRETNA: a graph theoretical network analysis toolbox for imaging connectomics. Frontiers in Human Neuroscience 2015;9:386.
100. Yeo BT, Krienen FM, Sepulcre J, Sabuncu MR, Lashkari D, Hollinshead M, et al. The organization of the human cerebral cortex estimated by intrinsic functional connectivity. Journal of Neurophysiology 2011;.
101. Liang Z, King J, Zhang N. Neuroplasticity to a single-episode traumatic stress revealed by resting-state fMRI in awake rats. NeuroImage 2014;103:485–491.
102. Pedregosa F, Varoquaux G, Gramfort A, Michel V, Thirion B, Grisel O, et al. Scikit-learn: Machine Learning in Python. Journal of Machine Learning Research 2011;12:2825–2830.
103. Zhou S, Luo J, Jiang Y, Wang H, Lu H, Gong G, Supporting data for "Group-specific discriminant analysis enhances detection of sex differences in brain functional network lateralization". GigaScience Database; 2025. http://gigadb.org/dataset/102700.
104. Zhou S, Luo J, Jiang Y, Lu H, Gong G, Human Connectome Project (HCP) Intrahemispheric FC. Zenodo; 2023. https://doi.org/10.5281/zenodo.10050233.
105. Zhou S, Luo J, Jiang Y, Lu H, Gong G, Brain Genomics Superstruct Project (GSP) Intrahemispheric FC. Zenodo; 2023. https://doi.org/10.5281/zenodo.10050234.
106. Zhou S, Luo J, Jiang Y, GSDA-Lateralization. [Computer software]. GitHub;. Accessed April 20, 2025. https://github.com/shuo-zhou/GSDA-Lateralization.
107. Zhou S, Luo J, Jiang Y, Wang H, Lu H, Gong G, Supporting data for "Group-specific discriminant analysis enhances detection of sex differences in brain functional network lateralization" [Computer software]. Software Heritage;. Accessed April 20, 2025. https://archive.softwareheritage.org/swh:1:snp:495f818df0e3c6d9ac1898b1cc14ec0ea396d98a.

Additional Files

Supplementary Fig. S1. Left vs. right brain classification results using GSDA-Logit on HCP data [45], employing two cross-validation strategies different from the one in Fig. 2. (A) Average test accuracy on the held-out session; for example, training was conducted on the 50% hemispheres same as in Fig. 2A from the REST1 session, and the test was performed on the data from the REST2 session. (B) GSI calculated from the test results shown in Supplementary Fig. S1A. (C) Average test accuracy on the held-out subjects' data; for example, training was conducted on the 80% subjects' data sampled from the REST1 session, and test was performed on the remaining 20% subjects' data from REST1 and REST2. (D) GSI calculated from the test results shown in Supplementary Fig. S1C. The remaining detailed descriptions of the figures, along with the main observations, are the same as those in the caption of Fig. 2.

Supplementary Fig. S2. Experimental results of left vs. right brain classification on male and female sets from the Brain Genomics Superstruct Project (GSP) [46] using GSDA-Logit with respect to the hyperparameter λ. (A) Average test accuracy on the held-out hemispheres, with a cross-validation strategy consistent with the one in Fig. 2A. (B) GSI calculated from the test results shown in Supplementary Fig. S2A. (C) Average test accuracy on the held-out subjects' data, with a cross-validation strategy consistent with the one in Fig. S1C. (D) GSI calculated from the test results shown in Supplementary Fig. S2C. The remaining detailed descriptions of the figures, along with the main observations, are the same as those in the caption of Fig. 2.

Supplementary Fig. S3. Pearson correlation coefficients between model weights learned from GSP data [46]. (A) Correlation between male-specific and multivariate control models. (B) Correlation between female-specific and multivariate control models. (C) and (D) Average pairwise correlation for (C) male-specific and (D) female-specific GSDA models. The main observations are consistent with those in Fig. 3.

Supplementary Fig. S4. Sex-specific lateralized connections identified by (A) male-specific models for HCP (Fig. 6A + Fig. 6E), (B) female-specific models for HCP (Fig. 6B + Fig. 6F), (C) male-specific models for GSP (Fig. 6C + Fig. 6G), and (D) female-specific models for GSP (Fig. 6D + Fig. 6G).

Supplementary Fig. S5. Count of the group “exclusive” lateralized connections for HCP and GSP (Fig. 6, E to H) categorized by associated lobes, and inter- or intra-lobe. The connections are identified by (A) male-specific models for HCP, (B) female-specific models for HCP, (C) male-specific models for GSP, (D) female-specific models for GSP. (E) Sum of Supplementary Fig. S4, A and C. (F) Sum of Supplementary Fig. S4, B and D.

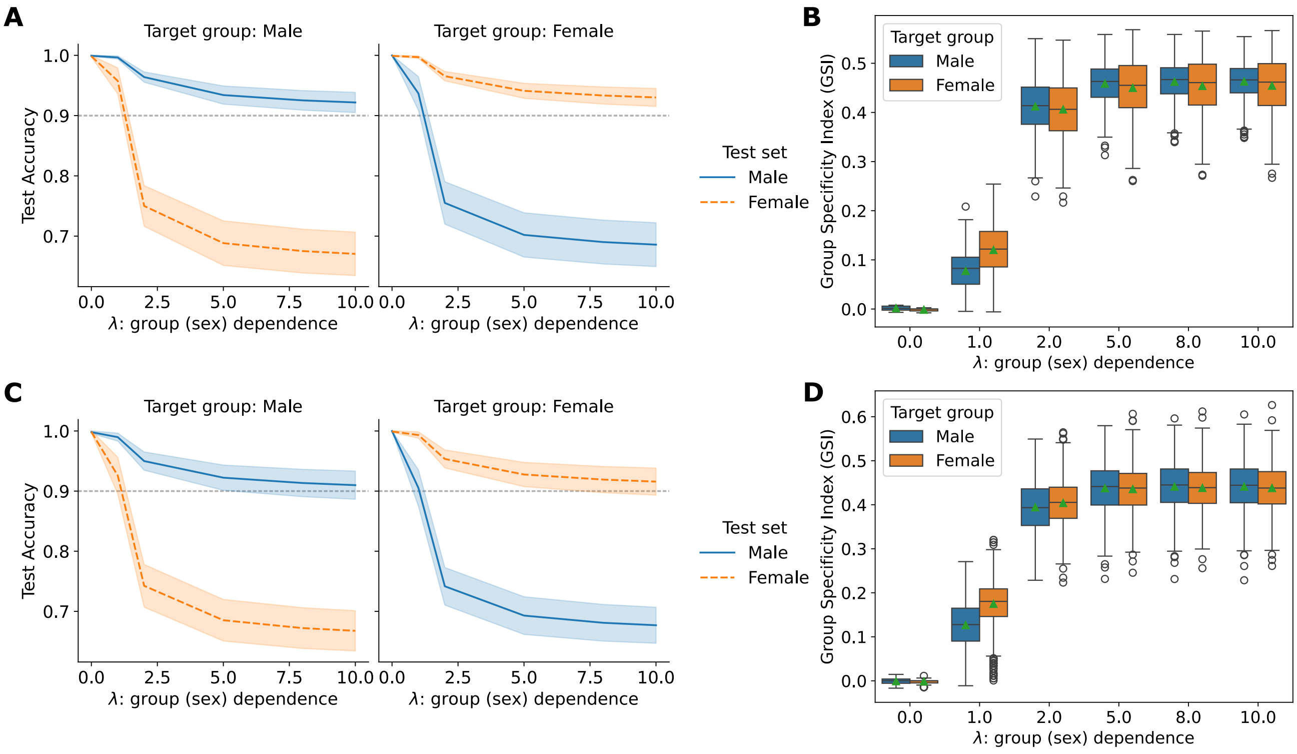

Supplementary Fig. S 1. Left vs. right brain classification results using GSDA-Logit on HCP data [45], employing two cross-validation strategies different from the one in Fig. 2. **(A)** Average test accuracy on the held-out session; for example, training was conducted on the 50% hemispheres same as in Fig. 2A from the REST1 session, and the test was performed on the data from the REST2 session. **(B)** GSI calculated from the test results shown in Supplementary Fig. S1A. **(C)** Average test accuracy on the held-out subjects' data; for example, training was conducted on the 80% subjects' data sampled from the REST1 session, and test was performed on the remaining 20% subjects' data from REST1 and REST2. **(D)** GSI calculated from the test results shown in Supplementary Fig. S1C. The remaining detailed descriptions of the figures, along with the main observations, are the same as those in the caption of Fig. 2.

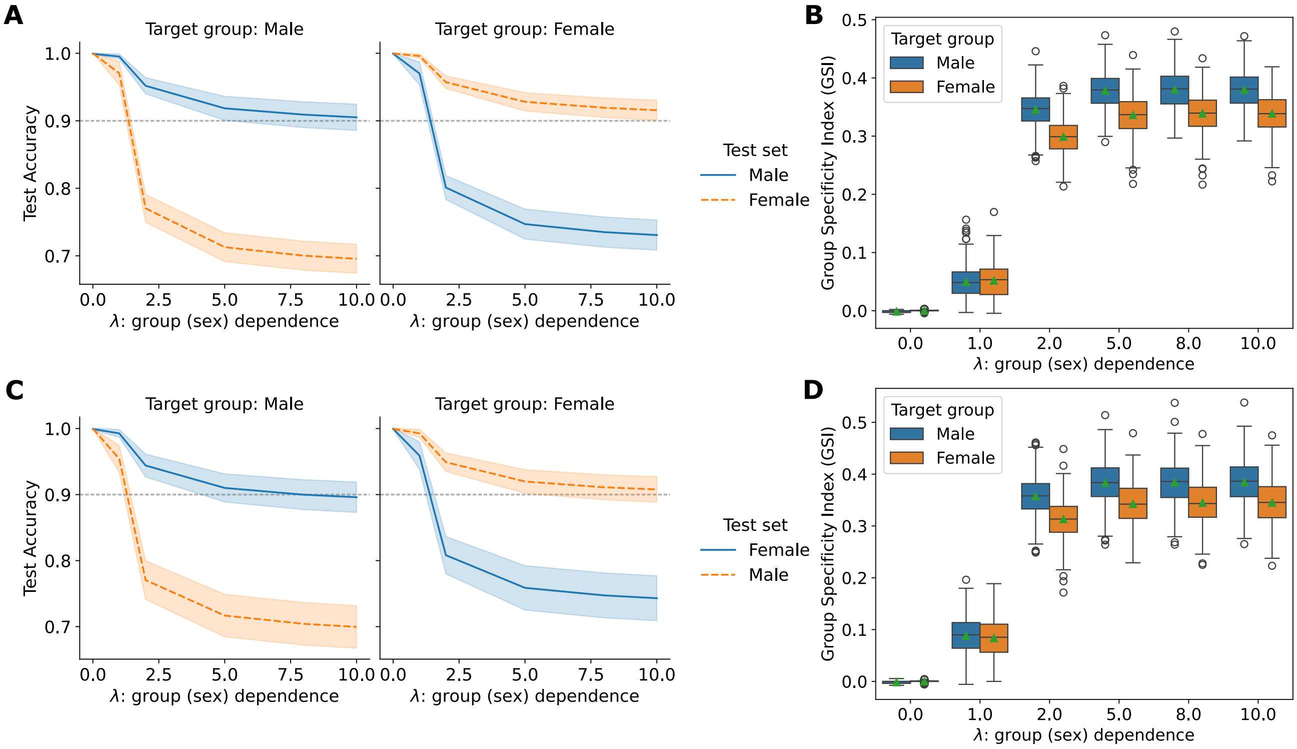

Supplementary Fig. S 2. Experimental results of left vs. right brain classification on male and female sets from the Brain Genomics Superstruct Project (GSP) [46] using GSDA-Logit with respect to the hyperparameter λ. **(A)** Average test accuracy on the held-out hemispheres, with a cross-validation strategy consistent with the one in Fig. 2A. **(B)** GSI calculated from the test results shown in Supplementary Fig. S2A. **(C)** Average test accuracy on the held-out subjects' data, with a cross-validation strategy consistent with the one in Supplementary Fig. S1C. **(D)** GSI calculated from the test results shown in Supplementary Fig. S2C. The remaining detailed descriptions of the figures, along with the main observations, are the same as those in the caption of Fig. 2.

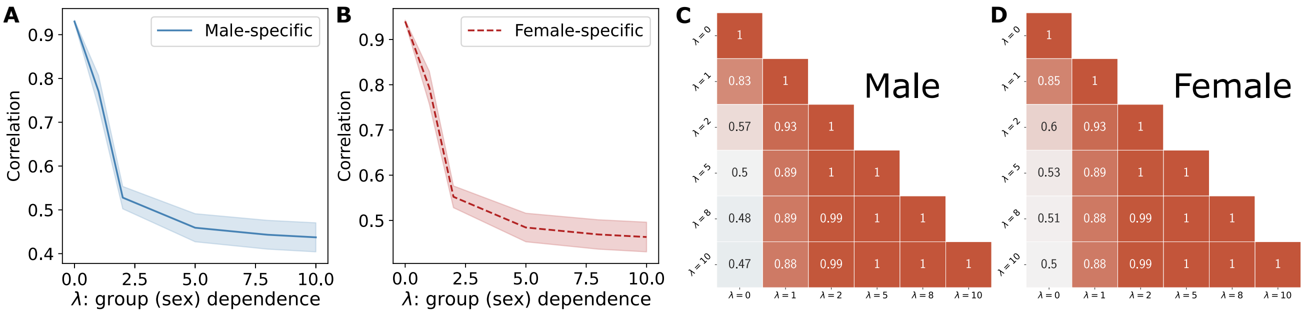

Supplementary Fig. S 3. Pearson correlation coefficients between model weights learned from GSP data [46]. (**A**) Correlation between male-specific and multivariate control models. (**B**) Correlation between female-specific and multivariate control models. (**C**) and (**D**) Average pairwise correlation for (**C**) male-specific and (**D**) female-specific GSDA models. The main observations are consistent with those in Fig. 3.

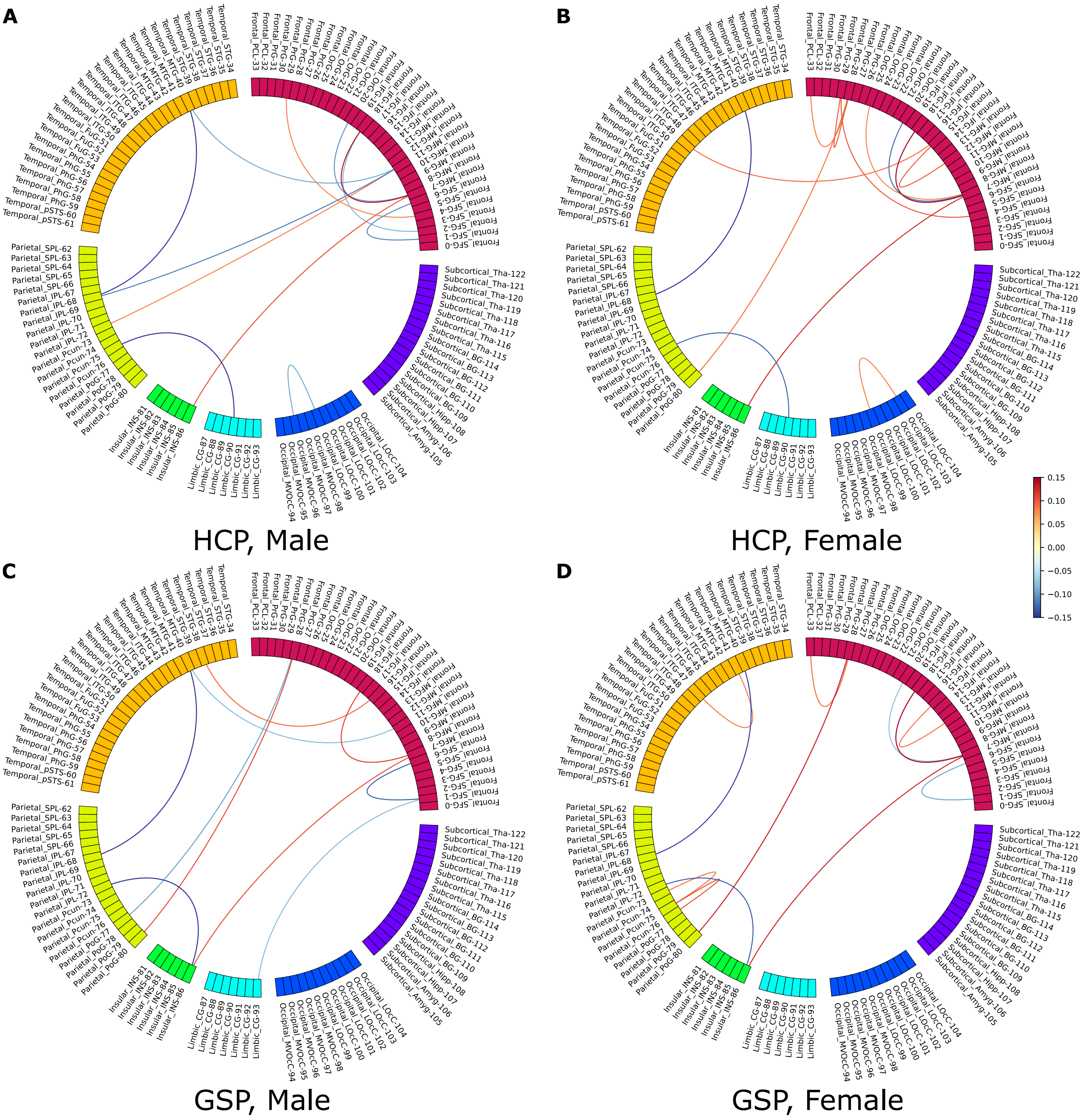

Supplementary Fig. S 4. Sex-specific lateralized connections identified by (**A**) male-specific models for HCP (Fig. 6A + Fig. 6E), (**B**) female-specific models for HCP (Fig. 6B + Fig. 6F), (**C**) male-specific models for GSP (Fig. 6C + Fig. 6G), and (**D**) female-specific models for GSP (Fig. 6D + Fig. 6G).

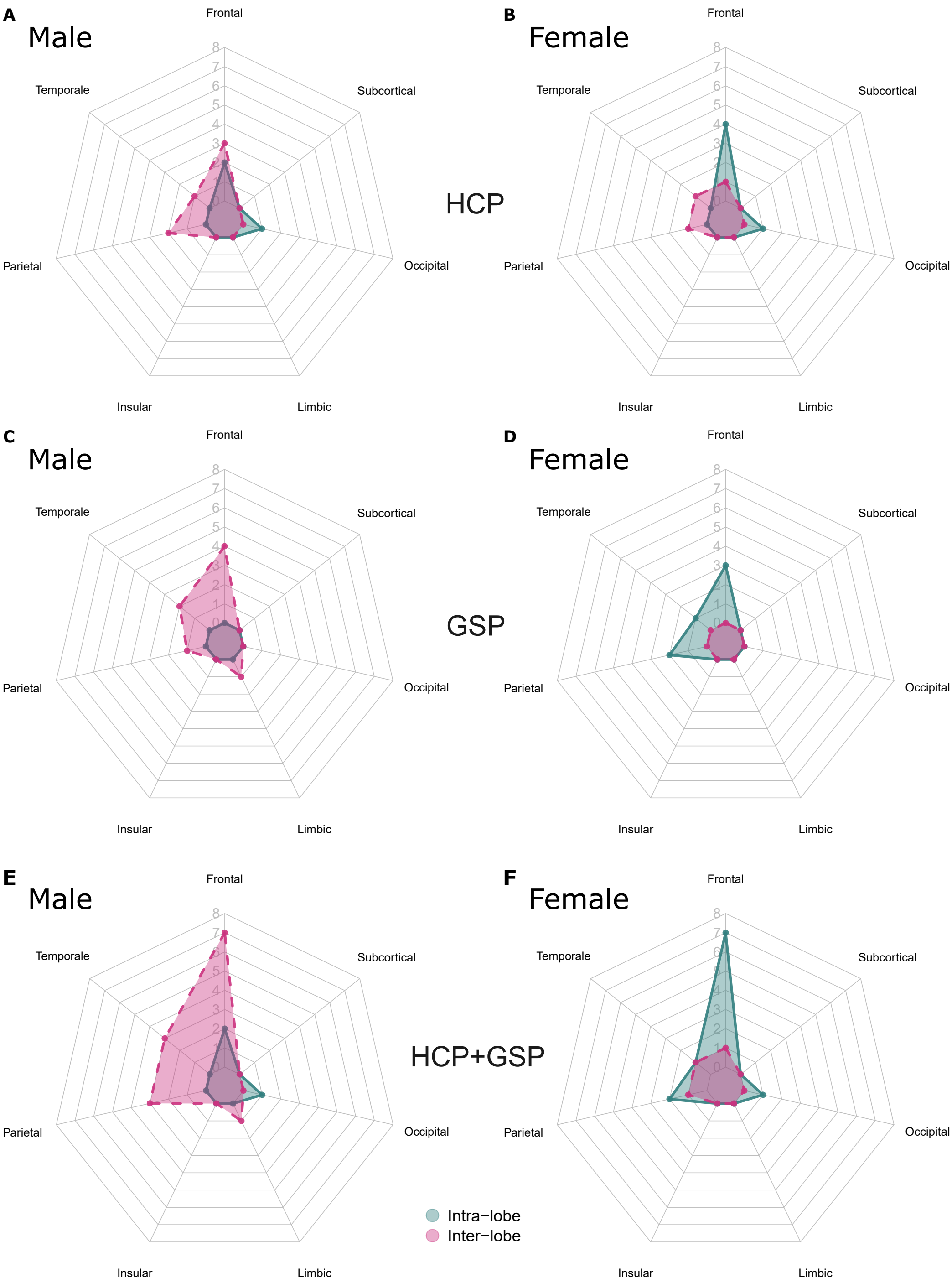

Supplementary Fig. S 5. Count of the group "exclusive" lateralized connections for HCP and GSP (Fig. 6, E to H) categorized by associated lobes, and inter- or intra-lobe. The connections are identified by **(A)** male-specific models for HCP, **(B)** female-specific models for HCP, **(C)** male-specific models for GSP, **(D)** female-specific models for GSP. **(E)** Sum of Supplementary Fig. S4, A and C. **(F)** Sum of Supplementary Fig. S4, B and D.

Response to reviewers

Click here to access/download
Supplementary Material
GIGA-D-24-00330 response to reviewers.pdf
