# Supplementary material for: Group-specific discriminant analysis enhances detection of sex differences in brain functional network lateralization
Source: Gigascience. 2025 Aug 30;14:giaf082. doi: 10.1093/gigascience/giaf082 (PMC12398281; doi:10.1093/gigascience/giaf082)
Supplement: giaf082_Reviewer_1_Report_Original_Submission — Fei Li -- 12/13/2024 [file giaf082_reviewer_1_report_original_submission.pdf]

Reviewer Report

Title: Group-specific discriminant analysis enhances detection of sex differences in brain functional network lateralization

Version: Original Submission **Date:** 12/13/2024

Reviewer name: Fei Li

Reviewer Comments to Author:

Here Zhou et al. introduced a new dual-classification model to explore the brain lateralization of brain functional connectivity in men and women. The rationale for this paper is well described and justified given the obstacles in previous brain lateralization analysis. The paper is well-written, comprehensive and detailed in methods. The analysis is complex, and the codes and procedure have been deposited publicly to ensure reproducibility. The sample size is big enough to have a validation and the results are promising making this new method possible to explore the lateralization alterations in brain disorders. I appreciate the opportunity to review the manuscript and would like to suggest the publication of this work, if the authors can clarify some points that I am concerned about.

1. A brief introduction to brain MRI applied to explore lateralization and sex-specific alterations in brain disorders, especially for some psychiatric disorders having an uneven incidence rate in male and female patients, would benefit the readers in understanding the importance and clinical significance of MRI application to explore brain lateralization and sex-specific neurobiology (DOI: 10.1088/1361-6560/ac9d1e; DOI: 10.1186/s12916-024-03313-2).
2. Recommend demonstrating the mean age and range of male and female participants respectively. Did the authors consider the mean age of men and women as a covariate in your classification model?
3. Did the authors use any method, such as Combat, to harmonize the multi-site imaging data before conducting your dual classification?
4. Neuroimaging biomarkers hold significant promise for predicting clinical and cognitive prognosis in brain disorders, as highlighted in recent studies (DOI: 10.1126/sciadv.adn1862; DOI: 10.1038/s41380-024-02710-6). To make the present paper more relevant to clinical significance, I recommend the authors add the above-mentioned references to talk about the future direction of brain MRI and dual classification in predicting the dynamic changes of clinical and cognitive ratings in brain disorders in the Potential implicationssection.

Methods

Are the methods appropriate to the aims of the study, are they well described, and are necessary controls included? Choose an item.

Conclusions

Are the conclusions adequately supported by the data shown? Choose an item.

Reporting Standards

Does the manuscript adhere to the journal's guidelines on [minimum standards of reporting?]() Choose an item.

Choose an item.

Statistics

Are you able to assess all statistics in the manuscript, including the appropriateness of statistical tests used? Choose an item.

Quality of Written English

Please indicate the quality of language in the manuscript: Choose an item.

Declaration of Competing Interests

Please complete a declaration of competing interests, considering the following questions:

- Have you in the past five years received reimbursements, fees, funding, or salary from an organisation that may in any way gain or lose financially from the publication of this manuscript, either now or in the future?
- Do you hold any stocks or shares in an organisation that may in any way gain or lose financially from the publication of this manuscript, either now or in the future?
- Do you hold or are you currently applying for any patents relating to the content of the manuscript?
- Have you received reimbursements, fees, funding, or salary from an organization that holds or has applied for patents relating to the content of the manuscript?
- Do you have any other financial competing interests?
- Do you have any non-financial competing interests in relation to this paper?

If you can answer no to all of the above, write 'I declare that I have no competing interests' below. If your reply is yes to any, please give details below.

I declare that I have no competing interests.

I agree to the open peer review policy of the journal. I understand that my name will be included on my report to the authors and, if the manuscript is accepted for publication, my named report including any attachments I upload will be posted on the website along with the authors' responses. I agree for my report to be made available under an Open Access Creative Commons CC-BY license (http://creativecommons.org/licenses/by/4.0/). I understand that any comments which I do not wish to be included in my named report can be included as confidential comments to the editors, which will not be published.

Choose an item.

To further support our reviewers, we have joined with Publons, where you can gain additional credit to further highlight your hard work (see: https://publons.com/journal/530/gigascience). On publication of this paper, your review will be automatically added to Publons, you can then choose whether or not to claim your Publons credit. I understand this statement.

Yes Choose an item.
