# Supplementary material for: Group-specific discriminant analysis enhances detection of sex differences in brain functional network lateralization
Source: Gigascience. 2025 Aug 30;14:giaf082. doi: 10.1093/gigascience/giaf082 (PMC12398281; doi:10.1093/gigascience/giaf082)
Supplement: giaf082_Reviewer_2_Report_Original_Submission — Guihua Jiang -- 12/27/2024 [file giaf082_reviewer_2_report_original_submission.pdf]

Reviewer Report

Title: Group-specific discriminant analysis enhances detection of sex differences in brain functional network lateralization

Version: Original Submission **Date:** 12/27/2024

Reviewer name: Guihua Jiang

Reviewer Comments to Author:

The study investigates gender differences in brain functional lateralization, particularly focusing on gender-specific patterns in brain networks during resting-state fMRI data. By introducing a novel Group-Specific Discriminant Analysis (GSDA) algorithm, along with cross-validation and a Group-Specific Index (GSI), the findings suggest that male brains exhibit greater left lateralization, while female brains demonstrate stronger right lateralization, especially in regions such as the prefrontal cortex. These results may provide insights into cognitive differences between genders, particularly in spatial and language abilities. This study provides a new perspective on the manifestation of gender differences in brain function lateralization and reveals the uniqueness of male and female brain networks through population-specific discriminant analysis methods. The following points need further improvements.
1)While the impact of gender differences on brain function is acknowledged, a deeper exploration of the neurobiological mechanisms underlying these differences and their specific effects on cognitive functions is warranted. A more comprehensive discussion, supported by relevant literature, would enhance the background.
2)The study mentions hypotheses regarding gender differences but lacks a clear articulation of the specific hypothesis and its theoretical support. Clearly stating the research hypothesis, along with its scientific background and justification, is essential.
3)The literature review is somewhat basic and lacks critical evaluation of existing studies. Expanding this section to highlight the innovative contributions of the current research and discussing the limitations of previous work would strengthen the study's foundation.
4)While gender-specific lateralized connections are described, a more detailed explanation of how these findings relate to cognitive functions is needed. Discussing potential mechanisms behind these differences would provide valuable insights.
5)The study summarizes its findings but lacks an in-depth exploration of the biological mechanisms behind these results. Expanding this discussion to integrate findings with existing literature would provide a more comprehensive biological explanation.
6)Limitations such as potential biases due to data source homogeneity are not adequately addressed. A detailed discussion of these limitations and suggestions for future studies would enhance the study's credibility.
7)While machine learning methods are mentioned, specific models used, hyperparameter adjustments, and cross-validation strategies are not detailed. Clearly listing these aspects would enhance the methodological rigor.

Methods

Are the methods appropriate to the aims of the study, are they well described, and are necessary controls included? Choose an item.

Conclusions

Are the conclusions adequately supported by the data shown? Choose an item.

Reporting Standards

Does the manuscript adhere to the journal's guidelines on minimum standards of reporting? Choose an item.

Choose an item.

Statistics

Are you able to assess all statistics in the manuscript, including the appropriateness of statistical tests used? Choose an item.

Quality of Written English

Please indicate the quality of language in the manuscript: Choose an item.

Declaration of Competing Interests

Please complete a declaration of competing interests, considering the following questions:

- Have you in the past five years received reimbursements, fees, funding, or salary from an organisation that may in any way gain or lose financially from the publication of this manuscript, either now or in the future?
- Do you hold any stocks or shares in an organisation that may in any way gain or lose financially from the publication of this manuscript, either now or in the future?
- Do you hold or are you currently applying for any patents relating to the content of the manuscript?
- Have you received reimbursements, fees, funding, or salary from an organization that holds or has applied for patents relating to the content of the manuscript?
- Do you have any other financial competing interests?
- Do you have any non-financial competing interests in relation to this paper?

If you can answer no to all of the above, write 'I declare that I have no competing interests' below. If your reply is yes to any, please give details below.

I declare that I have no competing interests.

I agree to the open peer review policy of the journal. I understand that my name will be included on my report to the authors and, if the manuscript is accepted for publication, my named report including any attachments I upload will be posted on the website along with the authors' responses. I agree for my report to be made available under an Open Access Creative Commons CC-BY license (http://creativecommons.org/licenses/by/4.0/). I understand that any comments which I do not wish to be included in my named report can be included as confidential comments to the editors, which will not be published.

Choose an item.

To further support our reviewers, we have joined with Publons, where you can gain additional credit to further highlight your hard work (see: https://publons.com/journal/530/gigascience). On publication of this paper, your review will be automatically added to Publons, you can then choose whether or not to claim your Publons credit. I understand this statement.

Yes Choose an item.
